# Selective agonists of KIR and NKG2A to evade missing self response of natural killer cells

**DOI:** 10.1038/s41598-025-18394-z

**Published:** 2025-09-29

**Authors:** Satoshi Hiura, Yuto Kuwasaki, Yosuke Nishikawa, Takako Kimura, Sayaka Yoshida, Makiko Nakayama, Tomohiro Makino, Suguru Ueno

**Affiliations:** 1 Discovery Research Laboratories V, Daiichi Sankyo Co., Ltd., Tokyo, Japan; 2 Modality Research Laboratories I, Daiichi Sankyo Co., Ltd., Tokyo, Japan; 3 Modality Research Laboratories II, Daiichi Sankyo Co., Ltd., Tokyo, Japan; 4 Bioprocess Technology Research Laboratories I, Daiichi Sankyo Co., Ltd., Tokyo, Japan; 5 Bioprocess Technology Research Laboratories II, Daiichi Sankyo Co., Ltd., Tokyo, Japan

**Keywords:** Allotransplantation, NK cells

## Abstract

Immune rejection is one of the most serious challenges in allogeneic transplantation, including allogeneic induced pluripotent stem cell (allo-iPSC)-derived cell therapy. Beta-2-Microglobulin gene-knockout, human leukocyte antigen (HLA) class I-deficient iPSCs can evade immune rejection by host T cells, which occurs due to HLA mismatches. However, natural killer (NK) cells recognize HLA class Ⅰ-deficient cells and reject them, which is known as the missing-self response. Introducing chimeric HLA-E protein to HLA class Ⅰ-deficient iPSCs suppresses the missing-self response of NK cells expressing the inhibitory receptor NKG2A; however, technology to suppress NKG2A-negative NK cells is still required. Here, we developed novel agonists for the other inhibitory receptor, killer immunoglobulin receptor (KIR), on NK cells. We found that antibodies that bind to activating KIR enhance NK cell activation and developed selective agonists for inhibitory KIRs (KIR2DL1, KIR2DL2/3, and KIR3DL1). Introducing these selective inhibitory KIR agonists on T cells and HLA class Ⅰ-deficient iPSCs allowed them to evade immune rejection by NK cells. Additionally, we identified an NKG2A-selective agonist as an alternative to chimeric HLA-E, which stimulates the activating receptor NKG2C. This technology enhances immune tolerance in allo-iPSCs and facilitates the development of various iPSC-derived regenerative medicines.

**Supplementary Information:**

The online version contains supplementary material available at 10.1038/s41598-025-18394-z.

## Introduction

Cell therapy is expected to provide promising solutions for many diseases. To date, autologous cells have primarily been utilized for cell therapy, but limitations such as high costs, time-consuming processes, and inconsistent quality hinder widespread application. In contrast to autologous cells, allogeneic cells are considered an ideal cell source because allogeneic cell therapy is cost-effective, reduces treatment waiting times, and ensures consistent product quality. However, immune rejection has prevented the widespread use of allogeneic cell therapy. To advance cell therapy, it is essential to overcome immune rejection of allogeneic cells and create a universally adaptable cell line.

T cells and natural killer (NK) cells are the main players of immune rejection^1–3^. Members of the highly polymorphic human leukocyte antigen (HLA) class Ⅰa (HLA-A, B, C) present a peptide fragment of proteins expressed in each cell, which are recognized by T-cell receptors (TCRs). TCRs identify HLA-peptide complexes and eliminate abnormal cells expressing mutated or exogenous proteins^4,5^. TCRs also recognize different HLA subtypes as abnormal proteins, leading T-cell activation and immune rejection. To prevent immune rejection by T cells due to HLA class Ⅰa mismatches, HLA class Ⅰa-deficient cells were developed by knocking out beta-2-microglobulin (B2M), an essential subunit of HLA class Ⅰa. Although these cells evade immune rejection by T cells^6–8^NK cells recognize HLA class Ⅰ-deficient cells and initiate rejection, known as the missing-self response^8–10^. NKG2A is an inhibitory receptor on NK cells, for which the non-polymorphic HLA class Ⅰb protein HLA-E serves as a ligand^11^. HLA-E expression suppresses NK cells without activating T cells via TCR recognition^12^. In addition to NKG2A, several other inhibitory receptors are also expressed on NK cells^13^. The killer immunoglobulin receptor (KIR) family, which recognizes HLA class Ⅰa, is another group of inhibitory receptors on NK cells^14^. Since NK cells exhibit diverse surface receptor expression profiles, they consist of a mixture of NKG2A^+^KIR^+^, NKG2A^+^KIR, NKG2A KIR^+^, and NKG2A KIR populations^15,16^. Thus, evading immune rejection by NK cells requires both NKG2A and KIR agonists for HLA class Ⅰa-deficient cells.

The KIR family consists of single-pass transmembrane receptors, including inhibitory receptors with long intracellular ITIM domains (designated as “L”) and activating receptors with short intracellular domains (designated as “S”)^14^. Among the inhibitory KIRs, KIR2DL1 recognizes the HLA-C2 group, while KIR2DL2 and KIR2DL3 recognize the HLA-C1 group^17^ classified based on polymorphisms at the 77th and 80th amino acids. KIR3DL1 also recognizes the Bw4 group of HLA-A and HLA-B, which contain specific amino acids at the 77th, 80th, and 83rd positions^18,19^. Autologous cells suppress KIR-expressing NK cells through HLA interactions, and KIR-expressing NK cells that genetically lack the corresponding HLA group become unresponsive to prevent autoimmune reactions. This mechanism, known as education or licensing, allows NK cells to target abnormal HLA-deficient cells or allogeneic cells expressing different HLA groups^19,20^. Meanwhile, activating KIRs have extracellular domain sequences that are almost identical to those of inhibitory KIRs but bind the same ligands with lower affinity^21^. Activating KIRs form complexes with DAP12, which contains an ITAM in its intracellular domain, activating NK cells upon ligand binding^14,22^.

The role of KIRs in immune rejection has been reported in organ transplantation, particularly kidney transplants, where donor-recipient matching considering KIR education reduces rejection risk^2^. Conversely, in hematopoietic stem cell transplantation for patients with hematological cancers, mismatched KIR education between donor and recipient can lead to immune rejection of cancer cells and a decreased risk of relapse^23^.

To overcome immune rejection of HLA class Ⅰa-deficient cells by NK cells, we aimed to obtain selective agonist binders for inhibitory KIRs that do not stimulate activating KIRs through phage display screening. Through the assessment of binding selectivity and agonistic activity, we developed selective agonist binders for inhibitory KIRs (KIR2DL1, KIR2DL2/3, KIR3DL1). Chimeric antigen receptor-T (CAR-T) cells or iPSCs expressing these binders as a membrane-bound single-chain Fv (scFv) successfully suppressed NK cell cytotoxicity. Additionally, we confirmed that the introduction of HLA-E stimulates the activating receptor NKG2C, as previously reported^24,25^. We also identified a selective NKG2A agonist binder that does not stimulate NKG2C. These selective agonist binders could provide solutions for immune rejection and open the way for creating a universal donor cell line.

## Result

### HLA-E expression was insufficient to suppress NKG2A-negative KIR-positive NK cell population observed among PBMC-derived NK cells

**Fig. 1:**
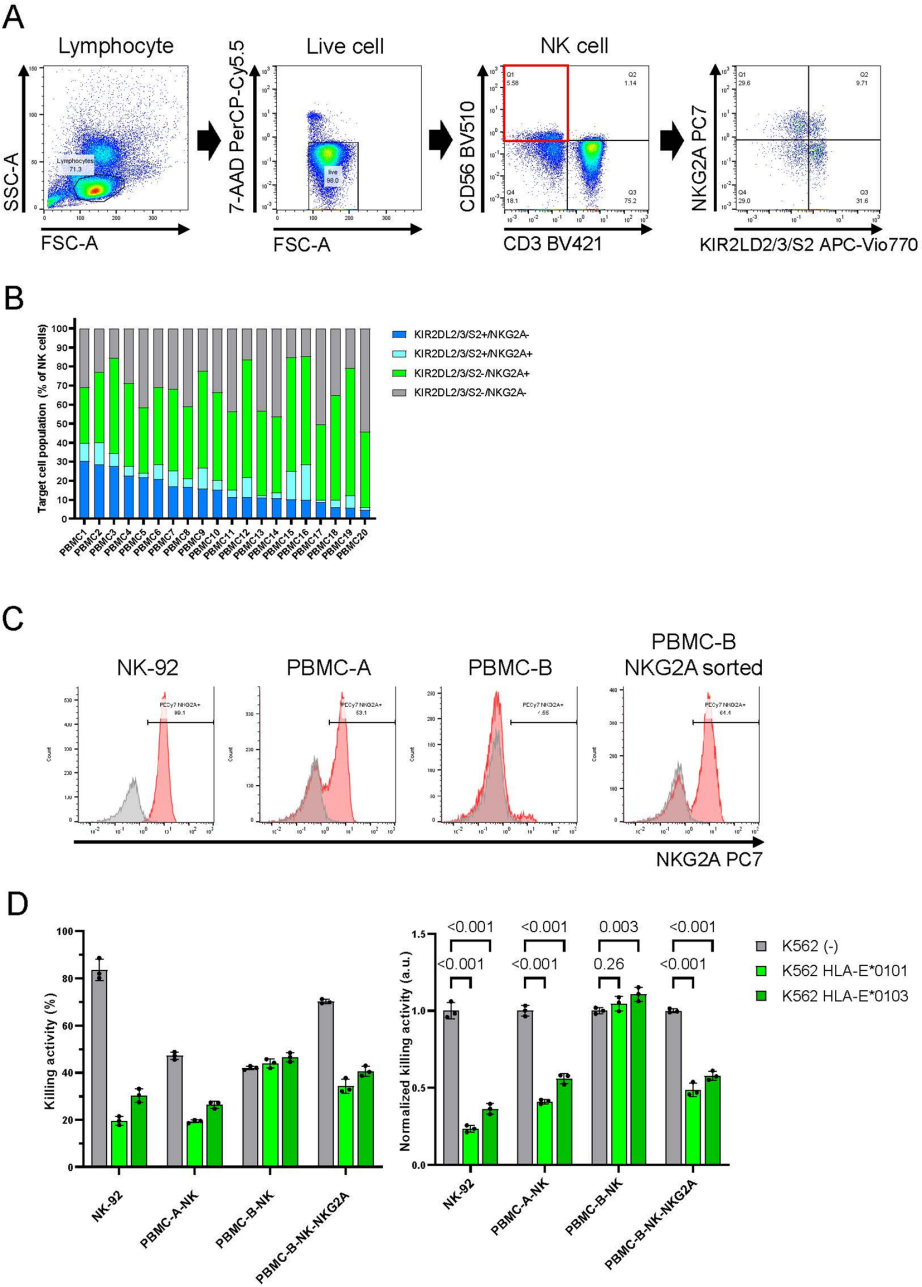
Overexpression of HLA-E failed to suppress cytotoxicity of NKG2A-negative NK cells. (**A**) Gating strategy for NK cell isolation from PBMCs used in this study. Lymphocytes were distinguished by SSC-FSC. 7AAD-negative cells were defined as live cells. CD3^−^/CD56^+^ cells were treated as NK cells (red box). (**B**) The expression of NKG2A and KIR2DL2/3/S2 on PBMC-derived NK cells was detected by flow cytometry from 20 PBMCs. The ratio of each pattern of NKG2A- and KIR-expressing NK cell populations in each PBMC is presented. (**C**) The expression of NKG2A on NK-92 and PBMC-derived NK cells was detected by flow cytometry and compared to NKG2A negative sorted PBMC-derived NK cells (gray). PBMC-derived NK cells were expanded using anti-CD16 antibody. CD3^−^/CD56^+^, NKG2A^+^ cells were sorted from PBMC-B using MACSQuant Tyto cell sorter. (**D**) NK-92 cells or PBMC-derived NK cells and K562 cells were co-cultured for 6 h (E: T = 1:1). Means and SDs of the dead K562 cell ratio (left) and the ratio normalized against the control (right) are shown (*n* = 3). The HLA-E alleles are indicated. *P*-values obtained by two-way ANOVA with Tukey’s multiple testing correction are presented. Source data are provided as a Source Data file.

To investigate the expression patterns of KIR and NKG2A receptors expressed on the NK cell surface, we performed flow cytometry analysis of peripheral blood mononuclear cells (PBMC)-derived NK cells from 20 donors. The CD3-negative/CD56-positive lymphocyte fraction was treated as NK cells (Fig. 1A). Consistent with a previous report^15^ we observed heterogeneous expression patterns of the inhibitory KIR and NKG2A receptors on NK cells, with the ratio of NK cells expressing each receptor varying among individuals (Fig. 1B). This analysis suggests that the suppression of cell lysis from NKG2A-positive NK cells alone might be insufficient for evading immune rejection by all NK cells. To evaluate whether the expression of HLA-E, a ligand for NKG2A, can prevent lysis by NK cells, we conducted a cytotoxicity assay using NK-92 cells (an NKG2A-expressing NK cell line) or PBMC-derived NK cells expressing varying levels of NKG2A against K562 cells, which lack endogenous HLA expression and are susceptible to NK cell-mediate killing. We prepared K562 cells expressing the tandem linked chimeric protein consisting of the HLA-G signal peptide, B2M, and HLA-E (chimeric HLA-E), consistent with a previous report (Supplementary Fig. 1)^12^. The NKG2A expression on NK cells was analyzed by flow cytometry (Fig. 1C). NKG2A-positive NK cells, including NK-92, PBMC-A-NK, and sorted PBMC-B-NK cells, exhibited reduced cytotoxicity against K562 cells expressing chimeric HLA-E compared with those without chimeric HLA-E (Fig. 1D). Conversely, NKG2A-negative PBMC-B-NK cells showed comparable cytotoxicity against both K562 cells with and without chimeric HLA-E (Fig. 1D). These results suggest that, to evade lysis by all NK cell types and escape immune rejection by NK cells, agonists targeting not only NKG2A but also other inhibitory receptors, such as KIRs, are required.

### Conventional anti-KIR antibodies suppressed NK cell cytotoxicity by agonizing KIR2DL2/3, while enhancing cytotoxicity by agonizing activating KIRs

**Fig. 2:**
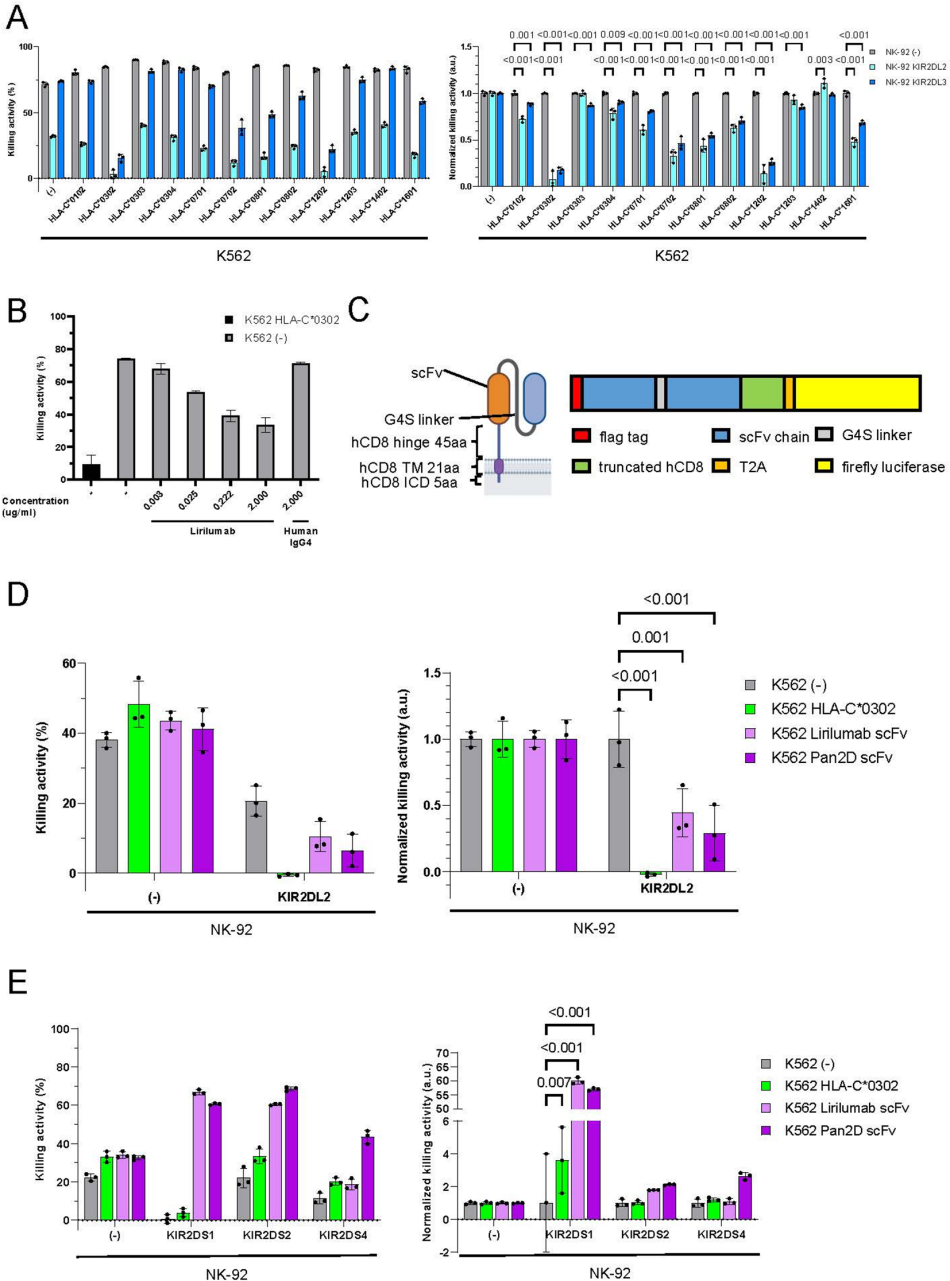
HLA-C and conventional anti-KIR antibody suppressed cytotoxicity of NK-92 cells expressing KIR2DL2, but activated NK-92 cells expressing KIR2DS1/2/4. (**A**) NK-92 cells and K562 cells were co-cultured for 6 h (E: T = 1:1). The KIR alleles KIR2DL2*001 and KIR2DL3*003 were used. Means and SDs of the dead K562 cell ratio (left) and the ratio normalized against the control (right) are shown (*n* = 3). *P*-values obtained by two-way ANOVA with Tukey’s multiple testing correction are presented. (**B**) NK-92 cells and K562 cells were co-cultured for 3 h (E: T = 3:1). The KIR allele KIR2DL2*003 was used. Means and SDs of the dead K562 cell ratio are shown (*n* = 2). (**C**) Schematic overview of membrane-bound anti-KIR antibody scFv with firefly luciferase for detection in cytotoxicity assay. (**D**) NK-92 cells and K562 cells were co-cultured for 8 h (E: T = 1:4). The KIR allele KIR2DL2*001 was used. K562 cells expressing membrane-bound anti-KIR antibody scFv with CD8 long hinge were used. Means and SDs of the dead K562 cell ratio (left) and the ratio normalized against the control (right) are shown (*n* = 3). *P*-values obtained by two-way ANOVA with Šídák’s multiple comparison test are presented. (**E**) NK-92 cells and K562 cells were co-cultured for 3 h (E: T = 1:3). The KIR alleles KIR2DS1*002, KIR2DS2*001, and KIR2DS4*001 were used. K562 cells expressing membrane-bound anti-KIR antibody scFv with CD8 long hinge were used. Means and SDs of the dead K562 cell ratio (left) and the ratio normalized against the control (right) are shown (*n* = 3). *P*-values obtained by two-way ANOVA with Tukey’s multiple testing correction are indicated. Source data are provided as a Source Data file.

We established an in vitro NK cell cytotoxicity assay to evaluate agonistic activity against KIR using NK-92 cells, a KIR-deficient NK cell line, and the K562 cell line. Inhibitory and activating KIRs were introduced to NK-92 cells (Supplementary Fig. 2), while HLA-C1 and C2 subtypes were introduced to K562 cells (Supplementary Fig. 3A, B). The findings on the cytotoxicity of KIR-expressing NK-92 cells against HLA-C1-expressing K562 cells revealed that some HLA-C1 subtypes, particularly C*0302, efficiently suppressed the cytotoxicity of NK-92 cells expressing KIR2DL2 and KIR2DL3 (Fig. 2A). Certain HLA-C2 subtypes also suppressed the cytotoxicity of NK-92 cells expressing KIR2DL1 (Supplementary Fig. 3C). Since HLA molecules can trigger TCR-mediated allogeneic activation of T cells, HLA-C expression does not serve evasion of immune rejection by both NK and T cells. Consequently, we investigated whether anti-KIR antibodies exhibit agonistic activity to KIRs. Lirilumab (BMS-986015, human IgG4) and Pan2D (NKVFS1, mouse IgG1) are known as antibodies against KIR2DL1/2/3^26,27^. These antibodies bind to KIR2DL1/2/3, blocking their association with HLA and enhancing the cytotoxicity of NK cells against cancer cells^28,29^. However, their agonistic activity against inhibitory KIRs has not been reported. To investigate the agonistic activity of anti-inhibitory KIR antibodies, soluble lirilumab IgG was added to a cytotoxicity assay of KIR2DL2-expressing NK-92 cells against K562 cells. Lirilumab demonstrated dose-dependent attenuation of the cytotoxicity of KIR2DL2 NK-92 cells (Fig. 2B). Soluble Pan2D IgG also exhibited similar agonistic activity against KIR2DL2 NK-92 cells (data not shown). These results indicate that the conventional anti-KIR antibodies lirilumab and Pan2D possess agonistic activity against inhibitory KIRs. Next, we tested whether the expression of single-chain Fv (scFv) derived from lirilumab or Pan2D on the cell surface can suppress the cytotoxic activity of NK-92 cells expressing KIRs. The scFv of each antibody was linked to truncated human CD8 protein for membrane expression (Fig. 2C, Supplementary Fig. 3D). The cytotoxicity assay showed that both lirilumab and Pan2D scFvs maintained agonistic activity against the KIRs and suppressed the cytotoxicity of KIR2DL2 NK-92 cells (Fig. 2D). However, lirilumab and Pan2D scFv also exhibited agonistic activity against activating KIR2DS1/2 and KIR2DS1/2/4, respectively, enhancing the cytotoxic activities of NK-92 cells (Fig. 2E). Lirilumab is known to bind to KIR2DL1/2/3 and KIR2DS1/2, while Pan2D binds to KIR2DL1/2/3 and KIR2DS1/2/4^26^. These results suggest that, to evade immune rejection by NK cells expressing KIRs, selective binding to inhibitory KIRs rather than activating KIRs is essential for KIR agonist antibodies.

### The KIR2DL2/3-selective agonist binder acquired by phage panning suppressed KIR2DL2/3-expressing NK cells without activating KIR2DS1/2/4-expressing ones

**Fig. 3:**
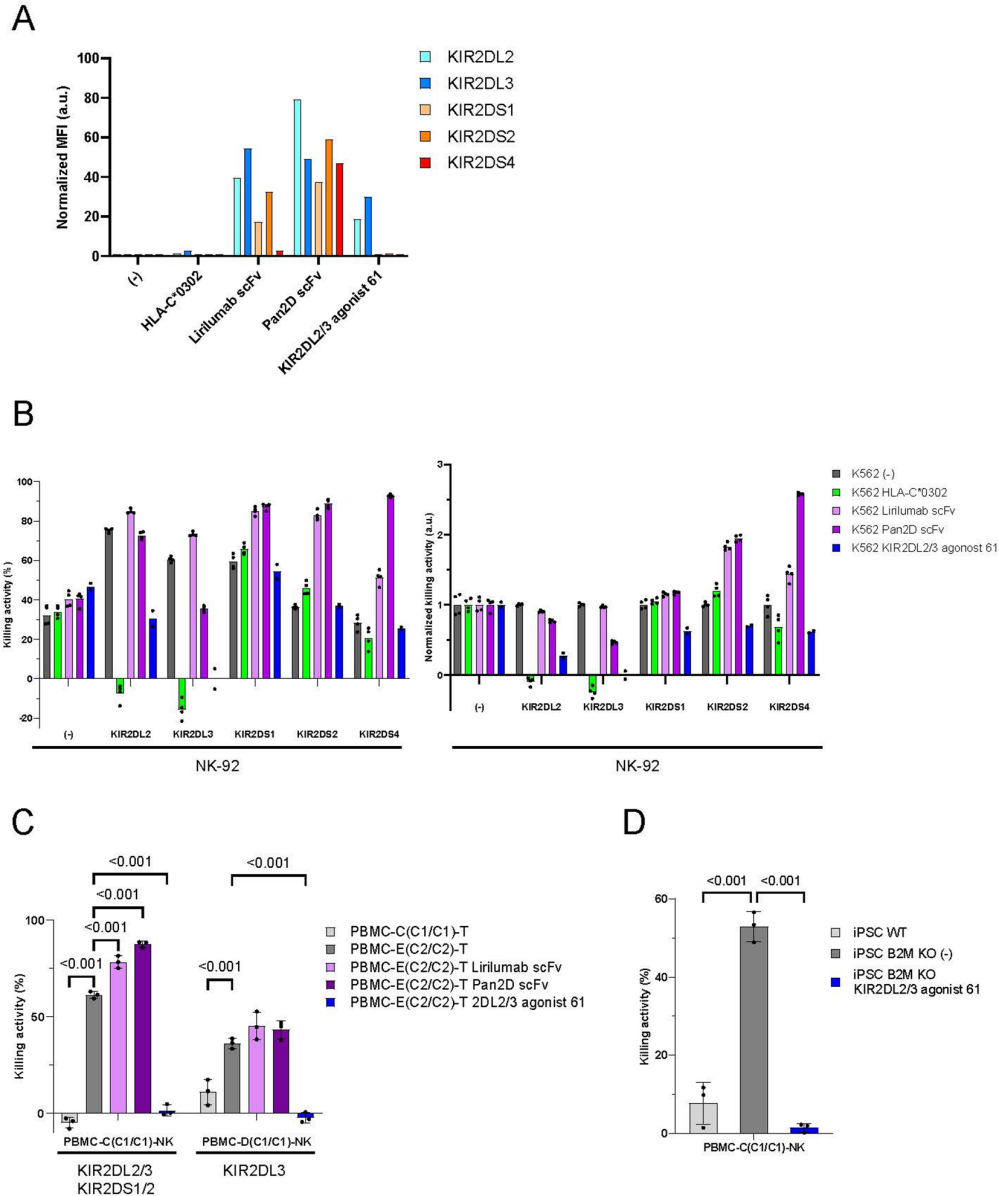
The KIR2DL2/3-specific scFv suppressed cytotoxicity of NK-92 cells expressing KIR2DL2/3 without activating KIR2DS1/2/4 and protected T cells and iPSCs from NK cells. (**A**) The binding affinity of KIR2DL2, KIR2DL3, KIR2DS1, KIR2DS2, and KIR2DS4 to HLA-C*0302 and membrane-bound anti-KIR antibody (lirilumab, Pan2D, KIR2DL2/3 agonist 61) scFvs expressed on K562 cells was assessed by flow cytometry (*n* = 1). The mean fluorescent intensity of each protein was normalized against that of control K562 cells. Information on the recombinant proteins is provided in Supplementary Table 9. (**B**) NK-92 cells and K562 cells were co-cultured for 6 h (E: T = 1:1). The KIR alleles KIR2DL2*001, KIR2DL3*001, KIR2DS1*002, KIR2DS2*001, and KIR2DS4*001 were used. Means and individual values of the dead K562 cell ratio (left) and the ratio normalized against the control (right) are shown (*n* = 4, *n* = 2 for KIR2DL2/3 agonist 61). (**C**) PBMC-derived NK cells and PBMC-derived T cells were co-cultured for 4 h (E: T = 10:1). Means and SDs of the dead T-cell ratio are shown (*n* = 3). *P*-values obtained by two-way ANOVA with Tukey’s multiple testing correction are indicated. Genetic background of PBMCs regarding KIRs is indicated. (**D**) PBMC-derived NK cells and iPSCs were co-cultured for 2 h (E: T = 1:1). Means and SDs of the dead iPSC ratio are shown (*n* = 3). P-values obtained by one-way ANOVA with Tukey’s multiple testing correction are indicated. PBMC-derived NK cells were expanded using anti-CD16 antibody. PBMC-C/D carries HLA-C1/C1/Bw4^−^ and PBMC-E carries HLA-C2/C2/Bw4^−^. Source data are provided as a Source Data file.

The sequence homology between inhibitory KIRs and activating KIRs, particularly between KIR2DL2 and KIR2DS2, is remarkably high, with only a few amino acid substitutions observed in their extracellular domains (Supplementary Fig. 4A). Since an antibody that selectively distinguishes KIR2DL2 and KIR2DS2 has rarely been reported and binds to KIR2DS2^30^, we performed phage display to develop a selective anti-inhibitory KIR antibody. To achieve the enrichment of highly selective antibodies, we repeated deselection and depletion of low-selective antibodies (Supplementary Fig. 4B). As a result of the phage display, we identified scFv 61, which specifically binds to inhibitory KIRs, demonstrating low binding affinity to activating KIR proteins in ELISA (data not shown). Flow cytometry using recombinant inhibitory KIR proteins confirmed that scFv 61 expressed on K562 cells (Supplementary Fig. 5A) selectively bound to inhibitory KIRs (Fig. 3A). We also assessed the cross-reactivity of scFv 61 against known inhibitory and activating receptors on NK cells. This scFv bound specifically to KIR2DL2/3 but not to other receptors on NK cells (Supplementary Fig. 5B). The cytotoxicity assay revealed that scFv 61 exhibits agonistic activity against KIR2DL2/3, but not against KIR2DS1/2/4 (Fig. 3B). We also evaluated NK-92 cell death in a cytotoxicity assay against K562 cells by detecting the leakage of labeled chelate from dead NK-92 cells, and found no NK-92 cell death due to KIR2DL2/3 agonist 61 (Supplementary Fig. 5C). This result rules out the possibility that the induction of cell death in NK-92 cells expressing activating KIRs caused protection from cell lysis mediated by KIR2DL2/3 agonist 61.

We next investigated whether KIR2DL2/3 agonist 61 expressed on PBMC-derived T cells can suppress PBMC-derived NK cells. T cells from HLA-C2/C2/Bw4-negative (C2/C2/Bw4^−^) donors cannot stimulate KIR2DL2/3 on NK cells from C1/C1/Bw4^−^ donors and are susceptible to attack from them, while T cells from (C1/C1/Bw4^−^) donors can, as endogenous HLA-C1 stimulates them. We expanded NK cells from the PBMCs of a C1/C1/Bw4 donor and T cells from the PBMCs of a C2/C2/Bw4 donor or a C1/C1/Bw4 donor. Lirilumab, Pan2D, and KIR2DL2/3 agonist 61 scFv were introduced into T cells from the C2/C2/Bw4 donor to compare their susceptibility against KIR2DL2/3-expressing NK cells from the C1/C1/Bw4 donor (Supplementary Fig. 6A). The T cells expressing KIR2DL2/3 agonist 61 effectively suppressed NK cells from the C1/C1/Bw4 donor, similar to the findings for C1/C1/Bw4 donor-derived T cells or autologous T cells (Fig. 3C). In contrast, control T cells and T cells expressing lirilumab or Pan2D scFv were killed by NK cells from the C1/C1/Bw4 donor. Since C1/C1/Bw4 donor-derived NK cells expressed a low level of KIR2DS1, and the KIR2DS2 gene was confirmed by PCR, we postulated that lirilumab and Pan2D scFv activate these activating KIRs, negating their agonistic activity against inhibitory KIRs. This indicates that KIR2DL2/3 agonist 61 can suppress primary NK cell cytotoxicity and evade immune rejection. We also examined the activity of KIR2DL2/3 agonist 61 expressed on iPSCs. KIR2DL2/3 agonist 61 was introduced into iPSCs in which B2M was genetically knocked out, which lack all HLA class I expression (Supplementary Fig. 6B), and these were co-cultured with KIR2DL2/3-expressing NK cells from the C1/C1/Bw4^−^ donor. While B2M-KO iPSCs were killed by NK cells from the C1/C1/Bw4^−^ donor, the expression of KIR2DL2/3 agonist 61 suppressed NK cell cytotoxicity to levels comparable to that of WT, HLA class I-intact iPSCs (Fig. 3D). Collectively, these results suggest that the agonistic activity of KIR2DL2/3 agonist 61 is selective and sufficient to evade immune rejection by NK cells.

### X-ray crystal structure analysis revealed the epitope of KIR2DL2/3 agonist 61 and predicted steric hindrance that contributes to binding selectivity

**Fig. 4:**
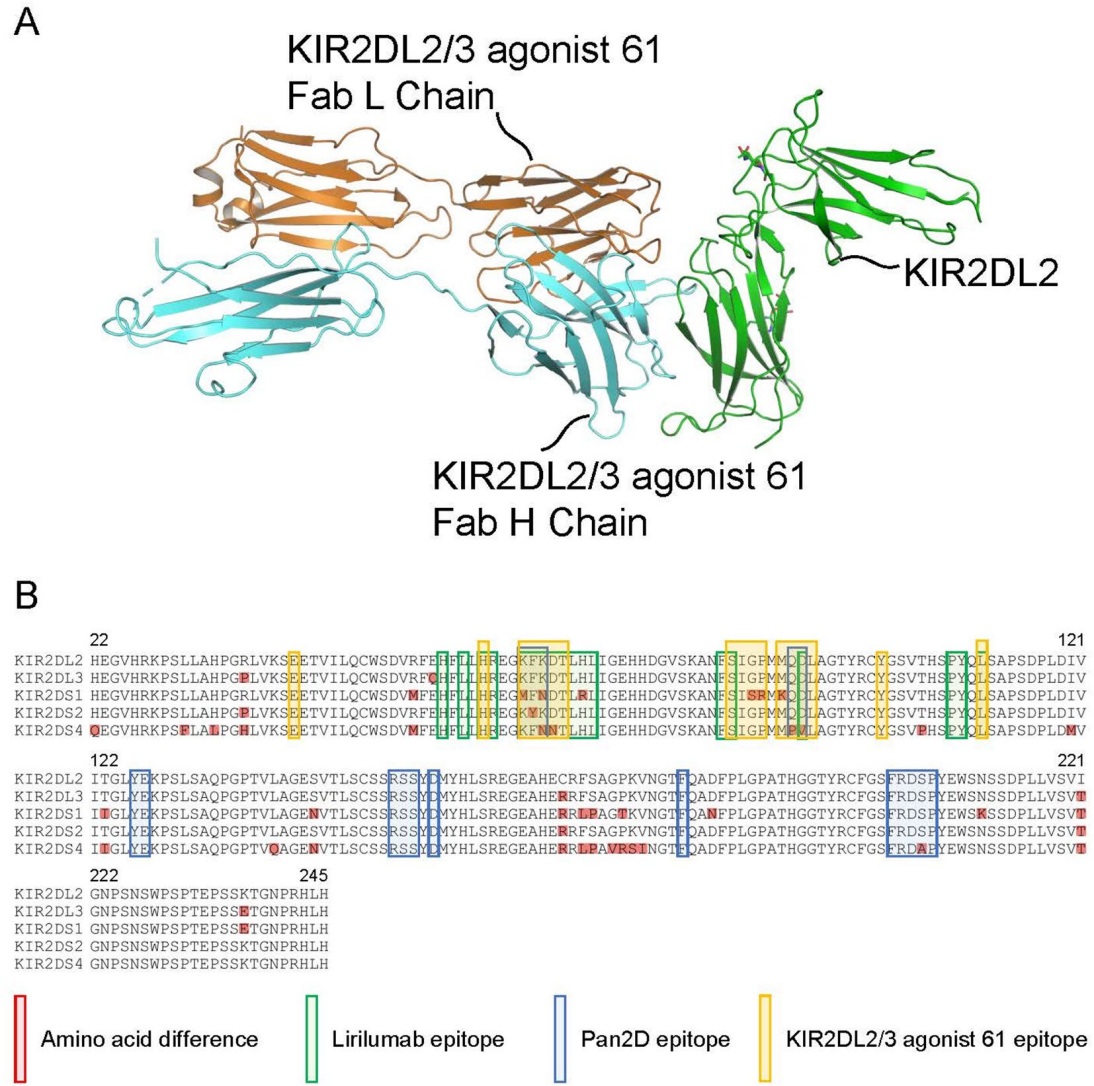
The complex of KIR2DL2/3 agonist 61 and KIR2DL2 obtained by X-ray crystal structure analysis predicted the epitope of KIR2DL2/3 agonist 61. (**A**) The complex of ribbon diagrams representing KIR2DL2/3 agonist 61 Fab and KIR2DL2 determined using the X-ray crystal structure analysis is shown. The green ribbon indicates KIR2DL2. The orange and cyan ribbons indicate KIR2DL2/3 agonist 61 Fab L chain and H chain, respectively. The KIR allele KIR2DL2*001 was used for X-ray crystal structure analysis. (**B**) Amino acid sequences of KIR family members were aligned. Amino acids differing from those of KIR2DL2 in other KIR family members are highlighted in red. The KIR alleles KIR2DL2*001, KIR2DL3*001, KIR2DS1*002, KIR2DS2*001, and KIR2DS4*001 were used. The epitopes of lirilumab (green boxes), Pan2D (blue boxes), and KIR2DL2/3 agonist 61 (yellow boxes) against KIR2DL2 determined by X-ray crystal structure analysis are highlighted in the sequence alignment.

To identify the epitope of KIR2DL2/3 agonist 61 and explain its selectivity, we performed X-ray crystal structure analyses of the KIR2DL2 complex with KIR2DL2/3 agonist 61 and Pan2D. Fab fragments and scFvs of each antibody were used for crystal structure determination. Unlike HLA-C or Pan2D, KIR2DL2/3 agonist 61 Fab bound only to domain 1 (Fig. 4A, Supplementary Fig. 7A)^31^. The epitopes of each antibody determined from the crystal structures were highlighted in the sequence alignment of inhibitory and activating KIRs (Fig. 4B). KIR2DL2/3 agonist 61 scFv formed similar interactions with KIR2DL2 compared to the Fab (Supplementary Fig. 7B). Additionally, a hydrogen-deuterium exchange mass spectrometry (HDX-MS) experiment was performed using KIR2DL2/3 agonist 61 scFv and KIR2DL2. The KIR2DL2 fragments containing the epitope of KIR2DL2/3 agonist 61 were protected from heavy hydrogen exchange (Supplementary Fig. 7C). Therefore, the identified epitope of KIR2DL2/3 agonist 61 shown in Fig. 4B is reliable and it covers different amino acids in activating/inhibitory KIRs compared with the epitope of lirilumab or Pan2D (Supplementary Table 1), suggesting its selectivity towards inhibitory KIRs.

To explain the selectivity of KIR2DL2/3 agonist 61 in detail, we prepared homology models of KIR2DL3 and KIR2DS1/2/4 and evaluated the differences in the epitope region. A major difference in amino acids between KIR2DL2 and KIR2DL3 was not expected to affect the binding ability of KIR2DL2/3 agonist 61, lirilumab, and Pan2D. Meanwhile, some amino acid differences from KIR2DL2 to KIR2DS1/2/4 that could cause steric hindrance or loss of hydrogen bonds may impair the binding of KIR2DL2/3 agonist 61 to KIR2DS1/2/4 (Supplementary Table 2, Supplementary Fig. 8A). These differences were expected to have little or no effect on the binding ability of Pan2D (Supplementary Table 2, Fig. 8B). Regarding lirilumab, these differences were expected to confer the loss of binding to KIR2DS4 but not to KIR2DS1/2 (Supplementary Table 2, Supplementary Fig. 8C). The results of homology modeling are consistent with the results of recombinant protein binding assay (Fig. 3A).

### Suppression of NK cells by KIR2DL2/3 agonist 61 helped survival of T cells in vitro and in vivo and sustained anti-CD19 chimeric antigen receptor (CD19 CAR) activity in vitro

**Fig. 5:**
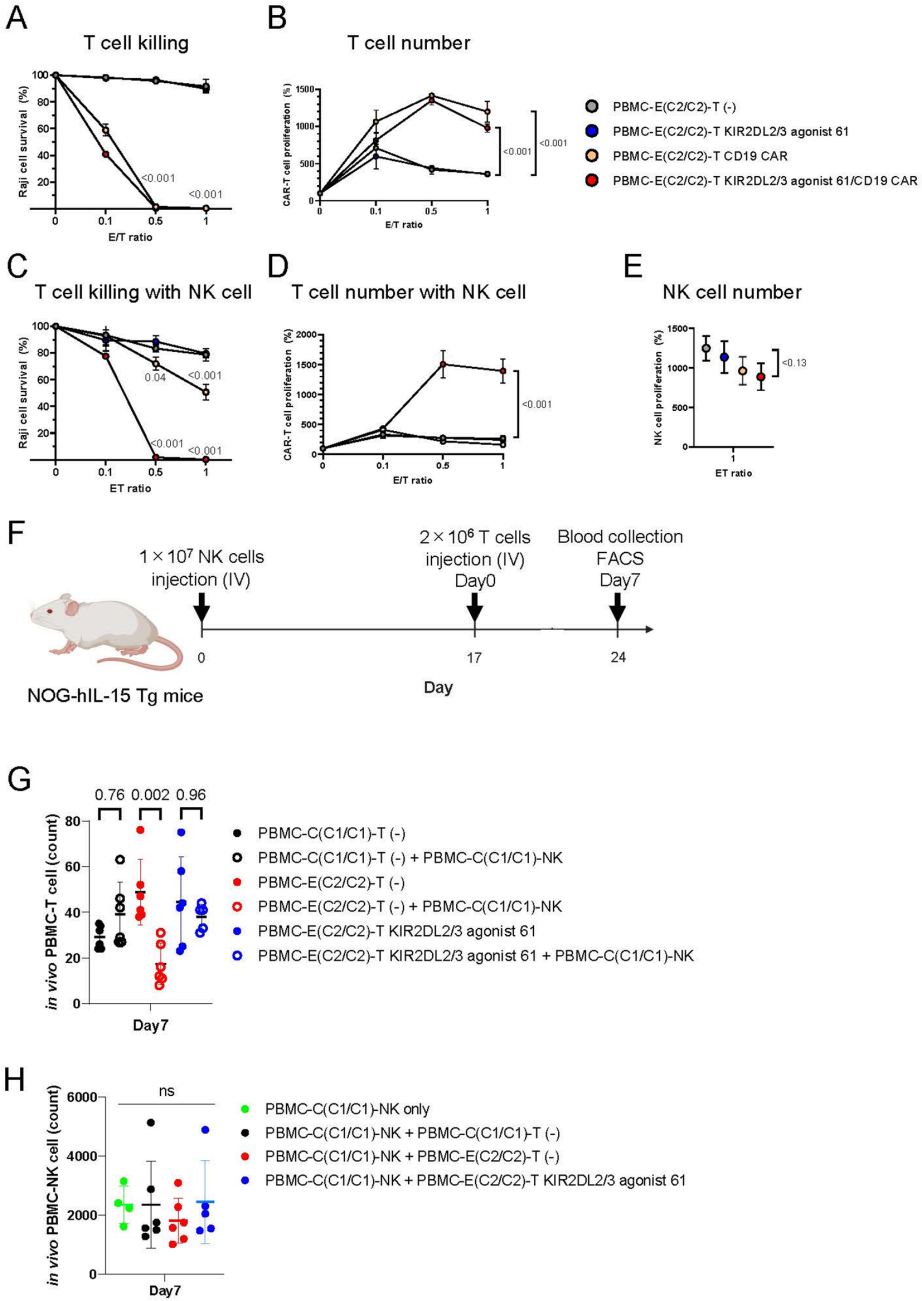
T cells expressing KIR2DL2/3 agonist 61 evade immune rejection by HLA-mismatched NK cells in vitro and in vivo, which contributes to the maintenance of killing activity of CAR-T cells in vitro. (**A**–**E**) PBMC-derived NK cells, PBMC-derived T cells, and Raji cells were co-cultured for 4 days [E (T cells): T (Raji cells) = 0.1:1, 0.5:1, and 1:1]. The number of NK cells was fixed at 1 × 10^4^ cells. Means and SDs of the dead Raji cell ratio are shown (**A**, **C**) (*n* = 3). Means and SDs of the proliferation ratio of T cells co-cultured with Raji cells compared with T cells without co-culture are shown (**B**, **D**) (*n* = 3). Means and SDs of the proliferation ratio of NK cells co-cultured with T cells compared with NK cells without co-culture are shown $\euro ({\text{n}}\,=\,{\text{3}})$. *p*-values obtained by two-way ANOVA with Tukey’s multiple testing correction (**A**–**D**) or one-way ANOVA with Tukey’s multiple testing correcti€(E) are presented. PBMC-derived NK cells were expanded using anti-CD16 antibody. PBMC-C carries HLA-C1/C1/Bw4^−^ and PBMC-E carries HLA-C2/C2/Bw4^−^. F) Timeline of PBMC-derived NK and T-cell transplantation in vivo. G) The number of T cells expressing control truncated NGFR or KIR2DL2/3 agonist 61 in peripheral blood at day 7 was detected by flow cytometry. Means and SDs of the number of T cells are shown (*n* = 6, *n* = 5 for PBMC-E-T KIR2DL2/3 agonist 61 + PBMC-C-NK). p-values obtained by one-way ANOVA with Tukey’s multiple testing correction are indicated. H) The number of NK cells expressing human CD56 in peripheral blood at day 7 was detected by flow cytometry. Means and SDs of the number of NK cells are shown (*n* = 6, *n* = 5 for PBMC-E-T KIR2DL2/3 agonist 61 + PBMC-C-NK). Source data are provided as a Source Data file.

Next, to investigate the applicability of KIR agonists in CAR-T therapy, we examined whether CAR-T cells introduced with KIR agonists could maintain their proliferation and cytotoxic activity against target cells in the presence of HLA-mismatched NK cells during co-culture. We introduced KIR2DL2/3 agonist 61 and anti-CD19 CAR into T cells derived from a C2/C2/Bw4^−^ donor (Supplementary Fig. 9A). A truncated NGFR served as a tag for the control PBMC-derived T cells. We evaluated the killing activity of CAR-T cells targeting CD19-positive Raji cells co-cultured with KIR2DL2/3-expressing NK cells from a C1/C1/Bw4^−^ donor (Supplementary Fig. 6A). In the absence of NK cells, both T cells expressing only anti-CD19 CAR and T cells expressing both anti-CD19 CAR and KIR2DL2/3 agonist 61 effectively killed Raji cells (Fig. 5A). T-cell proliferation was observed in both cases (Fig. 5B). However, in the presence of NK cells, T cells expressing only anti-CD19 CAR showed reduced killing activity against Raji cells, while the dual-expressing T cells maintained their killing activity (Fig. 5C). The dual-expressing T cells proliferated, whereas the T cells expressing only anti-CD19 CAR did not (Fig. 5D). The expression of KIR2DL2/3 agonist 61 did not affect NK cell proliferation (Fig. 5E). Thus, KIR2DL2/3 agonist 61 protected T cells from allogeneic NK cells, allowing them to sustain killing activity.

We next assessed the in vivo activity of KIR2DL2/3 agonist 61 on T cells using human IL-15 transgenic mice transplanted with human PBMC-derived NK cells^32^. NK cells from a C1/C1/Bw4 donor and T cells from either the same or a C2/C2/Bw4 donor, with or without KIR2DL2/3 agonist 61, were administered (Fig. 5F, Supplementary Fig. 9B). Then, blood samples were analyzed by flow cytometry. To evaluate immune rejection by NK cells, we measured the number of human PBMC-derived CD3-positive T cells. We found that T cells from the C2/C2/Bw4 donor were reduced in the presence of NK cells from the C1/C1/Bw4 donor compared to those without NK cells, whereas autologous T cells from the C1/C1/Bw4 donor and T cells expressing KIR2DL2/3 agonist 61 from the C2/C2/Bw4 donor did not show a reduction in cell numbers with NK cells (Fig. 5G). The presence of T cells or expression of KIR2DL2/3 agonist 61 did not affect the number of human CD56^+^ NK cells in blood samples (Fig. 5H). Surface expression of KIR and NKG2A on NK cells remained unchanged post-transplantation, regardless of the allogeneic reaction against T cells (Supplementary Fig. 9C). These results indicate that KIR2DL2/3 agonist 61 is effective in evading immune rejection by NK cells both in vitro and in vivo.

### Combination of KIR2DL1, KIR2DL2/3, and KIR3DL1 agonists was effective at suppressing NK cells expressing inhibitory KIRs without stimulating activating KIRs

**Fig. 6:**
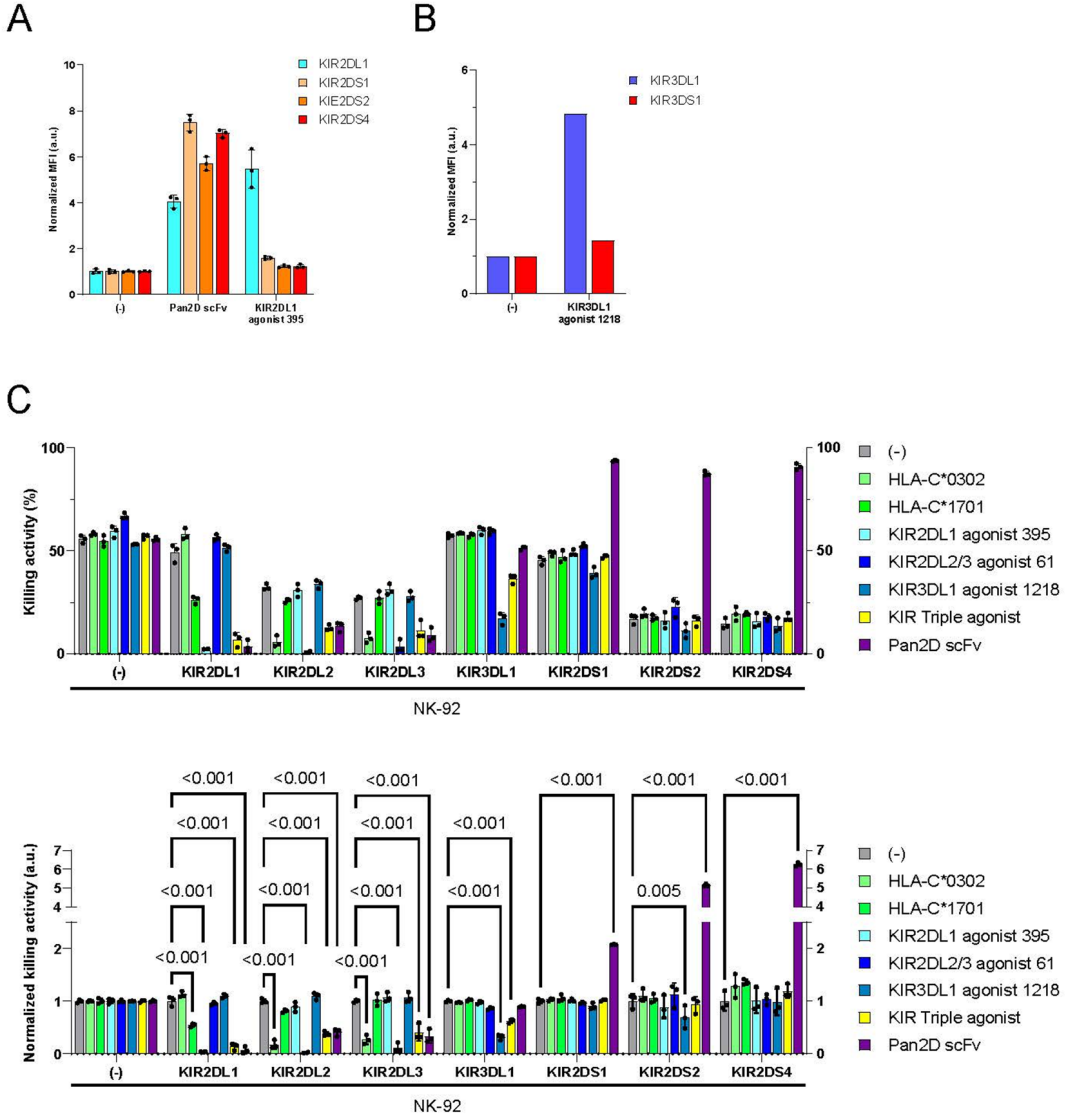
Combination of the KIR2DL1, KIR2DL2/3, and KIR3DL1 agonists efficiently suppressed cytotoxicity of NK-92 cells expressing inhibitory KIRs. (**A**) The binding affinity of KIR2DL1, KIR2DS1, KIR2DS2, and KIR2DS4 to membrane-bound anti-KIR antibody (Pan2D, KIR2DL1 agonist 395) scFvs expressed on K562 cells was assessed by flow cytometry (*n* = 3). The mean fluorescent intensity of each protein was normalized against that of control K562 cells. (**B**) The binding affinity of KIR3DL1 and KIR3DS1 to membrane-bound anti-KIR3DL1 antibody 1218 scFvs expressed on K562 cells was assessed by flow cytometry (*n* = 1). The mean fluorescent intensity of each protein was normalized against that of control K562 cells. Information on the recombinant proteins is provided in Supplementary Table 9. (**C**) NK-92 cells and K562 cells were co-cultured for 3 h (E: T = 1:1). The KIR alleles KIR2DL1*002, KIR2DL2*001, KIR2DL3*001, KIR2DS1*002, KIR2DS2*001, and KIR2DS4*001 were used. Means and SDs of the dead K562 cell ratio (top) and the ratio normalized against the control (bottom) are shown (*n* = 3). *p*-values obtained by two-way ANOVA with Tukey’s multiple testing correction are indicated. Source data are provided as a Source Data file.

To evade immune rejection by NK cells, HLA class Ⅰa-deficient cells require an additional inhibitory KIR agonist along with the KIR2DL2/3 agonist. KIR2DL1, which binds to the HLA-C2 subtype, and KIR3DL1, which binds to HLA-Bw4 subtype, also play crucial roles in NK cell-mediated immune rejection. The sequence homology between KIR2DL1 and KIR2DS1/2/4 is remarkably high, as is the homology between KIR3DL1 and KIR3DS1 (Supplementary Fig. 10). To obtain selective agonist binders for KIR2DL1 and KIR3DL1, we performed phage display, similar to our previous approach for acquiring the KIR2DL2/3 agonist. The selective scFvs identified through ELISA were expressed on the cell surface, and their binding to recombinant KIR proteins was assessed. We identified the selective scFv 395 and 1218 that bound to KIR2DL1 or KIR3DL1 but not to KIR2DS1/2/4 or KIR3DS1 (Fig. 6A, B). These selective scFvs against KIR2DL1 and KIR3DL1 were introduced into K562 cells along with KIR2DL2/3 agonist 61 for a cytotoxicity assay (Supplementary Fig. 11). To prevent undesirable clustering of each scFv, cysteine residues in the CD8 hinge domain were substituted with serine to eliminate disulfide bond formation. The cytotoxicity assay showed that the selective KIR2DL1 395 and KIR3DL1 1218 scFvs efficiently suppressed NK-92 cells expressing inhibitory KIRs without stimulating activating KIRs (Fig. 6C). The triple expression of KIR2DL1, KIR2DL2/3, and KIR3DL1 agonists on K562 cells suppressed all NK-92 cells expressing the target inhibitory KIRs. Because we could not create NK-92 cells expressing KIR3DS1, we used KIR3DS1-positive sorted PBMC-derived NK cells to assess whether KIR3DL1 agonist 1218 stimulates KIR3DS1 and activates NK cells (Supplementary Fig. 12A). K562 cells expressing KIR3DL1 agonist 1218 did not activate PBMC-derived NK cells expressing KIR3DS1 (Supplementary Fig. 12B).

Regarding each KIR receptor, various polymorphisms have been reported worldwide^33^. We referenced the Allele Frequency Net Database (AFND) and selected relatively common subtypes among different ethnic groups (Supplementary Table 3). These selected subtypes were expressed on NK-92 cells (Supplementary Fig. 13) and assessed for cytotoxicity against K562 cells expressing KIR agonists. The results showed that each KIR agonist efficiently suppressed the cytotoxicity of NK-92 cells expressing KIR subtypes (Supplementary Fig. 14). This indicates that we successfully obtained selective major inhibitory KIR agonists suitable for broad populations.

### The NKG2—selective agonist binder outperformed chimeric HLA-E in preventing the activation of NKG2C and suppressed NK cell cytotoxicity

**Fig. 7:**
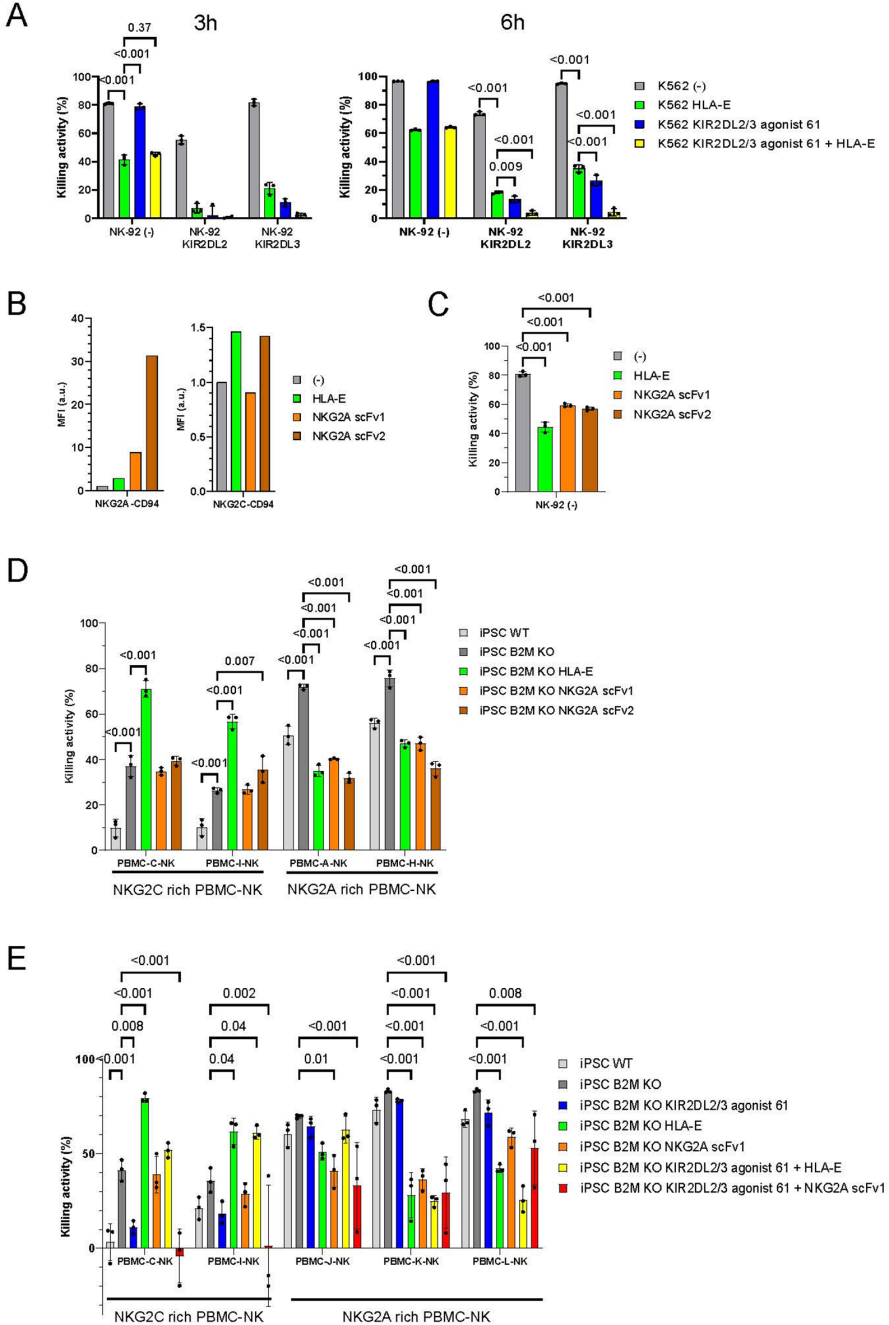
The NKG2A selective agonist suppressed cytotoxicity of NKG2A-expressing NK cells without activating NKG2C-expressing NK cells. (**A**) NK-92 cells and K562 cells were co-cultured for 3 h (left) and 6 h (right) (E: T = 1:1). Means and SDs of the dead K562 cell ratio is shown (*n* = 3). *p*-values obtained by two-way ANOVA with Sidak’s multiple testing correction are indicated. The KIR alleles KIR2DL2*001 and KIR2DL3*001 were used. (**B**) The binding affinity of NKG2A-CD94 complex (left) or NKG2C-CD94 complex (right) to HLA-E or membrane-bound anti-NKG2A antibody scFvs was assessed by flow cytometry (*n* = 1). The mean fluorescent intensity was normalized against that of control K562 cells. (**C**) NK-92 cells and K562 cells were co-cultured for 3 h (E: T = 1:1). The dead K562 cell ratio is shown (*n* = 3). p-values obtained by one-way ANOVA with Tukey’s multiple testing correction are presented. D) PBMC-derived NK cells and iPSCs were co-cultured for 2 h (E: T = 1:1). Means and SDs of the dead iPSC ratio are shown (*n* = 3). p-values obtained by two-way ANOVA with Tukey’s multiple testing correction are presented. E) PBMC-derived NK cells and iPSCs were co-cultured for 3 h (E: T = 1:1). Means and SDs of the dead iPSC ratio are shown (*n* = 3). *p*-values obtained by two-way ANOVA with Tukey’s multiple testing correction are indicated. PBMC-derived NK cells were expanded using anti-CD16 antibody. The PBMCs used for this figure carry HLA-C1/C1/Bw4^−^. The HLA-E*0101 allele is used for this figure. Source data are provided as a Source Data file.

To evaluate the combined effect of KIR agonists and NKG2A agonists, we assessed cytotoxicity using K562 cells that co-express KIR2DL2/3 agonist 61 and chimeric HLA-E protein (Supplementary Fig. 15A) with KIR2DL2/3-expressing NK-92 cells. Since NK-92 cells endogenously express NKG2A, the chimeric HLA-E suppressed cytotoxicity of NK-92 cells, and simultaneous expression of KIR2DL2/3 agonist 61 enhanced this suppression in NK-92 cells expressing KIR2DL2/3 (Fig. 7A). The combination of KIR and the NKG2A agonists demonstrated an additive effect in suppressing NK cell activity.

HLA-E is also known to be a ligand for the activating receptor NKG2C, and the expression of chimeric HLA-E enhances the cytotoxicity of NK cells expressing NKG2C^24,25^. To selectively stimulate NKG2A, we investigated known NKG2A antibodies^34^. We expressed some of scFvs from these antibodies, scFv1, 2 and chimeric HLA-E on K562 cells (Supplementary Fig. 15B), assessing binding selectivity using recombinant heterodimer proteins of CD94 with NKG2A or NKG2C by flow cytometry. Both chimeric HLA-E and NKG2A scFvs bound to the recombinant NKG2A. While chimeric HLA-E and NKG2A scFv2 bound to NKG2C, NKG2A scFv1 did not (Fig. 7B). The cytotoxicity assay demonstrated that chimeric HLA-E, NKG2A scFv1, and scFv2 suppressed the cytotoxicity of NK-92 cells expressing NKG2A (Fig. 7C). These NKG2A agonists were then introduced into B2M-KO iPSCs (Supplementary Fig. 15C). For the cytotoxicity assay, we expanded NKG2A-rich PBMC-derived NK cells or NKG2C-rich PBMC-derived NK cells (Supplementary Fig. 16A). Chimeric HLA-E, scFv1, and scFv2 suppressed the cytotoxicity of NKG2A-rich NK cells compared with control B2M-KO iPSCs (Fig. 7D). In contrast, chimeric HLA-E enhanced the cytotoxicity of NKG2C-rich NK cells, while NKG2A scFv1 and did not. To evaluate the additive effect of NKG2A agonists and KIR agonists, chimeric HLA-E or NKG2A scFv1 was co-expressed with KIR2DL2/3 agonist 61 on B2M-KO iPSCs (Supplementary Fig. 15D), followed by a cytotoxicity assay using PBMC-NK cells from a C1/C1/Bw4^−^ donor (Supplementary Fig. 16B). While HLA-E weakened the suppressive activity of KIR2DL2/3 agonist 61 against NKG2C-rich NK cells when co-expressed on iPSCs, NKG2A scFv1 did not affect KIR2DL2/3 agonist 61 activity against NKG2C-rich NK cells and exhibited additive suppression of the cytotoxicity against NKG2A-rich NK cells (Fig. 7E). Although NKG2A scFv1 showed comparable or slightly weaker suppression of the cytotoxicity against NKG2A-expressing NK cells compared with HLA-E (Fig. 7C and D against PBMC-A-NK, Fig. 7E against PBMC-K-NK and PBMC-L-NK), iPSCs expressing NKG2A scFv1 were less susceptible to NK cell-mediated cytotoxicity than wild-type iPSCs. These findings suggest that the agonistic activity of NKG2A scFv1 is sufficient to evade immune rejection by NK cells expressing NKG2A. We concluded that NKG2A scFv1 is a selective NKG2A agonist suitable for combination with KIR agonists to suppress NK cells and prevent NKG2C activation, rather than chimeric HLA-E.

#### The KIR agonists effectively suppressed KIR educated PBMC-derived NK cells compared with other inhibitory receptor ligands

**Fig. 8:**
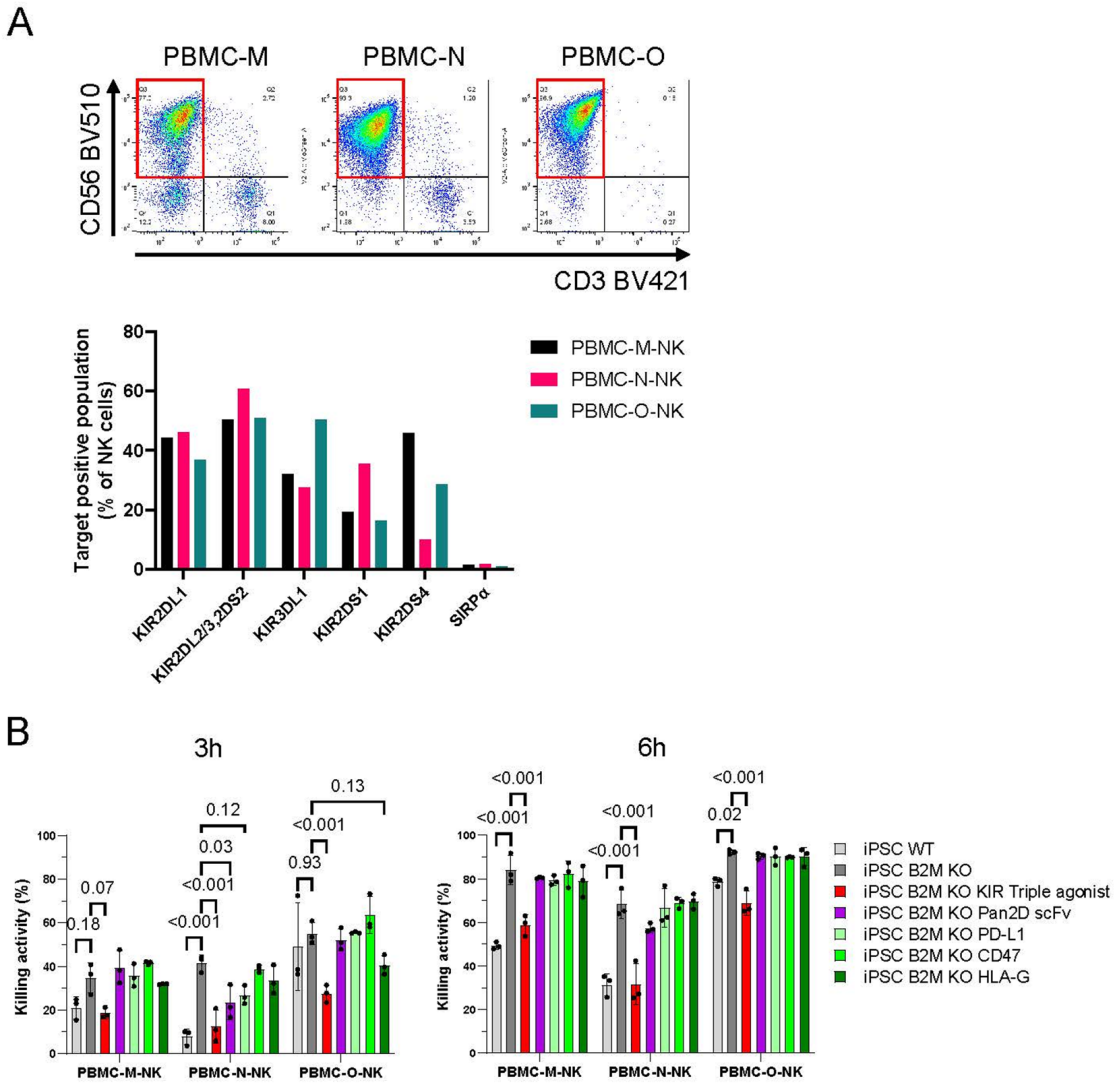
The iPSCs expressing triple KIR agonists suppressed NK cells. (**A**) The expression of KIR family members and SIRPa on PBMC-derived NK cells was detected by flow cytometry. CD3^−^/CD56^+^ cells were treated as NK cells (top, red frame). The expression ratio of each KIR family member and SIRPa on NK cells is presented (bottom) (*n* = 1). (**B**) PBMC-derived NK cells and iPSCs were co-cultured for 3 h (left) and 6 h (right) (E: T = 1:3). Means and SDs of the dead iPSC ratio are shown (*n* = 3). *p*-values obtained by two-way ANOVA with Tukey’s multiple testing correction are presented. PBMC-derived NK cells were expanded using feeder K562 cells expressing membrane-bound IL-21 and HLA-E. The PBMC-M, N, O carry HLA-C1/C2/Bw4^+^. Source data are provided as a Source Data file.

Finally, we evaluated current immune evasion technologies commomly used in the non-clinical stage: CD47, PD-L1 and HLA-G^35–37^. We introduced these ligands or KIR2DL1, KIR2DL2/3, and KIR3DL1 triple agonists into B2M-KO iPSCs (Supplementary Fig. 17). NK cells from a C1/C2/Bw4^+^ donor were expanded, confirming the expression of all relevant KIRs by flow cytometry (Fig. 8A). Unlike in the previous study, SIRPa was not expressed on the expanded NK cells^38^. We also analyzed SIRPa expression on PBMC-derived NK cells before the expanding culture, finding that SIRPa expression was limited to the monocyte fraction and there was only slight expression on lymphocyte, CD3-negative, CD56-positive NK cells (Supplementary Fig. 18). PD-1 and KIR2DL4 were also not detected on NK cells. In the cytotoxicity assay, triple KIR agonists effectively suppressed the cytotoxicity of NK cells from a C1/C2/Bw4^+^ donor expressing both educated inhibitory and activating KIRs (Fig. 8B). In contrast, the other ligands did not exhibit inhibitory activity against the expanded NK cells. While the immune environment of each organ of the body must be considered, these results suggest that selective agonist binders for inhibitory KIRs may be advantageous, at least under specific conditions, compared to existing immune evasion technologies.

## Discussion

Currently, the introduction of artificial HLA-E with B2M chimeric protein is being used in preclinical studies of allogeneic donor cell transplantation^39^. However, this approach faces two challenges. First, NK cells exhibit diverse surface receptor expression profiles, and chimeric HLA-E suppresses certain NK cell populations expressing NKG2A. Second, chimeric HLA-E stimulates the inhibitory receptor NKG2A as well as activating receptor NKG2C, enhancing cytotoxicity of NKG2C-expressing NK cells. In this study, we focused on another group of inhibitory receptors, KIRs, expressed on NK cells. To address the limitations of chimeric HLA-E, we established selective agonists for inhibitory KIRs (KIR2DL1, KIR2DL2/3, and KIR3DL1) and identified a selective agonist for NKG2A. Despite the high homology between inhibitory and activating KIRs, stringent phage display allowed us to obtain selective agonist binders. Epitope analysis by X-ray crystallography and homology modeling confirmed the high selectivity of KIR2DL2/3 agonist 61. Unlike conventional non-selective anti-KIR antibodies, our selective KIR agonists did not stimulate activating KIRs. They successfully suppressed KIR subtypes and protected CAR-T cells and iPSCs from NK cell-mediated cell lysis. These binders did not interact with other activating receptors on NK cells and demonstrated additive efficacy for NK cells expressing both inhibitory KIRs and NKG2A, allowing more precise control of immune rejection. To our knowledge, they are the first selective agonist binders for inhibitory KIRs and NKG2A. We also compared our selective KIR agonists to other allogeneic technologies, such as PD-L1, CD47, and HLA-G introduction. We found minimal or no expression of PD-1, SIRPa, or KIR2DL4, receptors for such technologies on NK cells; only the selective KIR agonists effectively suppressed NK cell cytotoxicity.

Regarding other inhibitory KIRs, some HLA-A subtypes have been identified to bind KIR3DL2^40^, but correlation of the HLA-A with allogeneic reactions mediated by KIR3DL2 is unclear^2,41^. KIR2DL4, known to induce both inhibitory and activating signals, binds HLA-G, though its correlation with allogeneic reactions has not yet been reported^42,43^. LILRB1/2, inhibitory receptors of the LILR family, are expressed on NK cells and recognize all members of HLA class Ⅰa^44^. While the role of LILRB1/2 in immune rejection is not fully understood, the expression of HLA-E or HLA-G may suppress NK cells expressing LILRB1/2^45,46^.

Genetically edited iPSCs with HLA-A and -B knockout and HLA-C monoallelic expression have been reported to evade immune rejection^47^. A cell bank limited to HLA-C requires fewer cell lines than a fully HLA-matched iPSC bank and is expected to suppress NK cells expressing KIR2DL1 or KIR2DL2/3. However, immune rejection by KIR3DL1-expressing NK cells remains a concern. Moreover, since classical HLA class Ⅰa presents minor antigen peptides, these cells may still face immune rejection by T cells, as reported in mice and primates^48,49^. Additionally, genetically modified iPSCs aimed at enhancing therapeutic efficacy will require the creation of new cell bank for each modified iPSC line. Recently, genetic ablation of adhesion molecules CD54 and CD58 for evading immune rejection was reported^50^. Combining such approaches with KIR agonist technology could further mitigate immune rejection by NK cells.

Transplantation of HLA class Ⅰa-deficient cells carries a risk of malignancy or infection, as HLA class Ⅰa alerts immune cells to abnormal conditions. Although knockout of HLA class Ⅰa, HLA-A, B, and C is essential to prevent immune rejection by T cells, preserving endogenous non-polymorphic HLA class Ⅰb, such as HLA-E and HLA-G, may support protective immunity. Expressing selective KIR agonists on HLA-A-, B-, and C-knockout cells, along with NKG2A agonist if the endogenous HLA-E expression is inadequate, would be optimal for a universal donor cell line. Meanwhile, developing a stable mechanism, such as a suicide gene switch, is necessary to eliminate transplanted cells with an undesirable profile^51^.

Our KIR agonist technology is expected to improve the likelihood of successful engraftment in iPSC-derived products currently undergoing clinical trials^52^. In addition to cell transplantation, allogeneic technologies can be applied to xenotransplantation, such as of porcine heart or kidney for patients seeking organ transplants^2,53^. So far, the introduction of HLA-E and matching HLA-C subtype has been applied to improve the graft survival of xenotransplants^54^. Furthermore, introducing HLA-E into CAR-T cells improved the efficacy of CAR-T-cell therapy by suppressing NK cell missing-self response^55^. As we indicated, selective KIR agonists and NKG2A agonist could be further utilized in this field.

In conclusion, we have developed selective KIR agonists and an NKG2A agonist that suppress immune rejection by NK cells. Our technology could lead to the creation of a universal donor cell line adaptable for any individual, which is essential for performing cell therapy across populations.

### Data availability

All data supporting the results are available in the main text or the supplementary materials. The source data for the main figures and extended data figures are provided as Source Data files. The DNA sequences of the vectors and plasmids used in this study are available upon corresponding author reasonable request. The crystal structures of KIR agonists in complex with KIR2DL2 have been deposited to the Protein data Bank (www.pdb.org) under the accession number (9LRF: 10.2210/pdb9LRF/pdb, 9LRH: 10.2210/pdb9LRH/pdb, 9LRA: 10.2210/pdb9LRA/pdb, Supplementary Table 11).

## Materials and methods

### Cell culture

K562 (CCL-243), Raji (CCL-86), and NK92 (CRL-2407) cell lines were purchased from ATCC. A Lenti-X HEK293T cell line (632180) was purchased from Takara Bio. An induced pluripotent stem cell (iPSC) line (773-3G) was purchased from REPEOCEL. Human peripheral blood mononuclear cells (PBMCs) were purchased from Cellular Technology Limited.

K562 and Raji cells were cultured in RPMI 1640 Medium, GlutaMAX (Life Technologies, 61870036), supplemented with 10% (v/v) FBS (Gibco, 10270-106) and 1% (v/v) Streptomycin/Penicillin (Thermo Fisher Scientific, 15240-062). NK92 cells were cultured in MyeloCult H5100 medium (STEMCELL Technologies, ST-05150) supplemented with 500 IU/mL Recombinant Human IL-2 (Nipro, 87–890; or Clinigen, Proleukin) and 1% (v/v) Streptomycin/Penicillin.

PBMC-derived NK (PBMC-NK) cells were cultured in MyeloCult H5100 medium supplemented with 1000 IU/mL Recombinant Human IL-2, 100 ng/mL Recombinant Human IL-18 (R&D Systems, 9124-IL) and 1% (v/v) Streptomycin/Penicillin. PBMC-derived T (PBMC-T) cells were cultured in AIM V Medium (Thermo Fisher Scientific, 12055083) supplemented with 10% (v/v) FBS and 100 IU/mL Recombinant Human IL-2. Lenti-X HEK293T cells were cultured in Dulbecco’s Modified Eagle’s Medium (DMEM) (Thermo Fisher Scientific, 10566-016) supplemented with 10% (v/v) FBS and 1% (v/v) Streptomycin/Penicillin. iPSCs were cultured in mTeSR Plus cGMP (Veritas, 100–0276) medium. A total of 4.8 µl/9 cm^2^ of iMatrix-511 silk (Nippi, 892021) and 1 µM CultureSureR Y-27,632 (Wako, 034-24024) were added when iPSCs were replated into new wells. These reagents were removed from media after the clustering of iPSCs. Accutase (ICT) (Funakoshi, AT104) was used to detach the iPSCs. All cell lines were cultured at 37 °C under 5% CO_2_. For cryopreservation, CellBanker1 (Takara Bio, CB011) was used. Stem-CellBanker GMP grade (Zenogen Pharma, 11924) was used for iPSC cryopreservation.

### CRISPR-Cas9 genome editing

Genome editing was conducted using the CRISPR-Cas9 scarless method^56^. B2M-knockout donor vector comprised *E. coli* Ori and an ampicillin resistance sequence from pcDNA3.1, left and right arm sequences amplified from the iPSC genome extracted using QuickExtract DNA extraction solution (Lucigen, QE09050), truncated human CD19 (tCD19), and EGFP protein (Supplementary Table 4) with a poly(A) sequence following the hUbC promoter inserted between the left and right arms. Plasmid px330 was used to express spCas9 protein and single guide RNA (sgRNA), similarly to the method in a previous report. The sequences of primers and sgRNAs are listed in Supplementary Table 5. b-Actin (ACTB) gene trap modification first donor vector comprised firefly luciferase, mCherry, and truncated human CD8 (Supplementary Table 4), instead of tCD19 and EGFP. Left and right arms for the ACTB donor vector were designed with the aim that inserted proteins were translated downstream of endogenous ACTB gene translation. Plasmids were constructed by Gibson assembly using NEBuilder HiFi DNA Assembly Master Mix [New England Biolabs (NEB), E2621L]. One microgram of each donor plasmid and specific sgRNA containing px330 were mixed into 20 µl of Opti-MEM I Reduced Serum Medium (Thermo Fisher Scientific, 31985070). A total of 2.5 × 10^5^ iPSCs or 2 × 10^5^ K562 cells were suspended with prepared Opti-MEM. P3 Cell Line 4D-Nucleofector X Kit S (Lonza, V4XP-3032) and 4D-Nucleofector (Lonza, AAF-1003B, AAF-1003X) were used for electroporation. Magnetic cell sorting was conducted around 4 days after electroporation using anti-CD19 Microbeads (Miltenyi Biotec, 130-050-301) or anti-CD8 Microbeads (Miltenyi Biotec, 130-045-201). LS columns (Miltenyi Biotec, 130-042-401) and autoMACS Running Buffer – MACS Separation Buffer (Miltenyi Biotec, 130-091-221) were also applied for magnetic cell sorting. Single-cell cloning by limiting dilution was applied if necessary.

### Vector construction

The lentiviral vectors used in this study were all constructed from a parental lentivector pLVSIN-EF1a IRES-ZsGreen1 (Takara Bio, 6191). The piggyBac donor vectors used in this study were all constructed from a parental vector: pPB[Exp]-EF1A > EGFP (VectorBuilder, VB900088-2258kkg). Each protein’s amino acid sequence was obtained from a database (Supplementary Table 4). Codon-optimized protein cDNA sequences were inserted between the promoter and WPRE or rBG poly(A) sequence, instead of a preexisting fluorescent marker, using Gibson assembly with NEBuilder HiFi DNA Assembly Master Mix.

Regarding HLA, B2M-(G4S)4 linker-flag tag (DYKDDDDK)-GSG linker was connected to the N-terminal of HLA without a signal sequence and firefly luciferase was placed downstream of HLA across a T2A self-cleavage peptide. The HLA-E tetramer complex was identical to that in a previous report^12^. The firefly luciferase placed downstream of truncated CD19 or NGFR (nerve growth factor receptor) across the T2A peptide was used as a control protein. HLA-C1 and HLA-C2 alleles tested in this paper were selected based on the allele frequencies obtained from 10 populations (Supplementary Table 6).

Regarding KIRs, the flag tag-GSG linker was inserted between the signal peptide and the N-terminal of KIR. In the case of KIRs without a flag tag, they were placed before truncated NGFR across T2A peptide in order to sort KIR-expressing cells.

Regarding the membrane-bound KIR agonist scFv, CD8 signal peptide and flag tag-GSG linker were connected to the N-terminal of scFv and a CD8 hinge was connected to the C-terminal of scFv. Each scFv chain was connected with a (G4S)3 linker. The firefly luciferase was placed downstream of membrane-bound scFv across the T2A peptide. The sequences of lirilumab and Pan2D (NKVSF1) were obtained from the published patents^26,27^. The sequences of specific agonists of KIR2DL2/3, KIR2DL1, and KIR3DL1 were obtained by phage panning. The sequence of anti-NKG2A antibody was obtained from the patent^34^. Anti-CD19 CAR construct was identical to that in a previous report^57^. Briefly, it contained the following, in order from the N-terminal: CD8-derived signal peptide, CD19CAR VL, (G4S)3 linker, CD19CAR VH, CD8-derived hinge region sequence, CD8-derived cell membrane domain for CAR, intracellular costimulatory molecule (4-1BB, CD3ζ), linker (GSG), T2A peptide, and blue fluorescent protein (BFP). Vector sequences are available upon request.

### Lentivirus preparation and infection

Lenti-X HEK293T cells were seeded at a density of 5 × 10^6^ cells per 10 cm dish 18–24 h prior to transfection. The prepared cells were co-transfected with Lentiviral High Titer Packaging Mix (Takara Bio, 6194) and lentiviral plasmids containing proteins of interest as explained above using the TransIT-293 Transfection Reagent (Takara Bio, MIR2704) mixed into Opti-MEM. The medium was replaced with a fresh one 24 h after transfection. The viral supernatant was collected 48 h after transfection, passed through a 0.45 μm filter (Cytiva, SLHVR33RB), supplemented with a one-third volume of Lenti-X concentrator (Takara Bio, 631232), incubated at 4 °C for 1 h, centrifuged to precipitate the virus particles, and then the viruses were resuspended in appropriate medium and stored at − 80 °C or − 150 °C.

For infection, a RetroNectin (T100B, Takara Bio)-coated plate was prepared. RetroNectin was diluted with phosphate-buffered saline (PBS) (Nacalai Tesque, 14249-24) to a concentration of 20–100 µg/mL and added to an untreated plate. After incubation for 2 h at 37 °C, the RetroNectin was removed and the plate was washed with PBS. Target cells suspended with each culture medium were mixed with the lentivirus in the RetroNectin-coated plate. Then, the plate was centrifuged at 1000 × g and 32 °C for 1 h and cultured at 37 °C under 5% CO_2_.

### Gene transduction using piggybac transposon system

The constructed piggyBac donor vector containing target proteins was mixed with Pbase-expressing vector pRP[Exp]-mCherry-CAG > hyPBase [VectorBuilder, pDNA(VB010000-9365tax)-P] at a ratio of 3:1. Four hundred nanograms of total plasmid was supplemented with 20 µl of Opti-MEM. A total of 2.5 × 10^5^ iPSCs or 2 × 10^5^ K562 cells were suspended with prepared Opti-MEM. P3 Cell Line 4D-Nucleofector X Kit S and 4D-Nucleofector were used for electroporation.

### Cell production

K562 cells expressing target proteins with firefly luciferase were generated using CRISPR-Cas9 genome editing, lentiviral transduction, or the piggyBac transposon system. Four days after each transduction, cells were magnetically sorted using APC anti-DYKDDDDK Tag Antibody (Biolegend, 637308) or APC anti-human HLA-A, B, C Antibody (Biolegend, 311410) as the primary antibody and anti-APC Microbeads (Miltenyi Biotec, 130-090-855).

NK92 cells expressing KIR were generated by lentiviral transduction. Four days later, the cells were magnetically sorted using LNGFR (CD271) Microbeads kit (Miltenyi Biotec, 130-099-023) or APC anti-DYKDDDDK Tag Antibody and anti-APC Microbeads.

CRISPR-Cas9 genome-edited B2M-KO iPSCs were transduced with the lentivirus or the piggyBac transposon system to express target proteins and firefly luciferase. Four days later, the cells were magnetically sorted using APC anti-DYKDDDDK Tag Antibody and anti-APC Microbeads.

For all cell lines, single-cell cloning by limiting dilution was applied if necessary. Expression of each protein was confirmed by flow cytometry.

### Preparation of PBMC-derived T cells

To obtain PBMC-derived T cells (PBMC-T) expressing the target proteins, 5 µg/mL anti-CD3 antibody (Biolegend, 317347) and 20 µg/mL RetroNectin were added to a 24-well untreated plate, followed by incubation at 37 °C for 2–4 h. The coated plate was washed with PBS after incubation. PBMCs were suspended at 1 × 10^6^ cells/2 ml in PBMC-T culture medium and added to a CD3, RetroNectin-coated plate with lentiviruses. The plate was centrifuged at 1000 × g and 32 °C for 60 min. The entire medium was replaced 4 days and 1 week after transduction, and cells were sorted using anti-LNGFR Microbeads or APC anti-DYKDDDDK Tag Antibody and anti-APC Microbeads. Then, the sorted cells were expanded for another 5–7 days. To enrich anti-CD19 CAR-expressing cells, 87.5 µg/mL recombinant human CD19 protein (OriGene, TP302922) was added to the 24-well untreated plate and incubated at 37 °C for 2–4 h to make an hCD19-coated plate. Anti-CD19 CAR-expressing lentivirus-infected PBMC-T cells were seeded in an hCD19-coated plate on day 8, followed by magnetic sorting on day 9 and harvesting on day 12. The HLA genotypes in PBMCs are summarized in Supplementary Table 12.

### Preparation of PBMC-derived NK cells

Anti-CD16 antibody (Biolegend, 302050) was diluted with PBS at 5 µg/mL and added to a 24-well untreated plate, followed by incubation at 37 °C for 2–4 h. PBMCs were suspended at 1 × 10^6^ cells/2 ml in PBMC-NK culture medium and added to a CD16-coated plate after washing it out. After 7–10 days, PBMC-NK cells were sorted using an NK cell isolation kit (Miltenyi Biotec, 130-092-657). These sorted cells were expanded for another 7–14 days.

To obtain more PBMC-NK cells, K562 cells expressing membrane-type IL-21 with chimeric HLA-E (feeder K562 cells) were used^58^. A total of 1 × 10^7^ of feeder K562 cells were suspended in RPMI medium supplemented with Mitomycin C Solution (Nacalai Tesque, 20898-21) at 10 µg/ml and incubated at 37 °C for 3 h. Feeder K562 cells were washed with RPMI medium, suspended in MyeloCult H5100 medium, and co-cultured with 1 × 10^7^ PBMCs suspended in MyeloCult H5100 medium supplemented with IL-2 at 200 U/mL cultured in a G-Rex 24Well Plate (Wilson Wolf Manufacturing Corporation, 80192 M). Mitomycin C-treated feeder K562 cells were added every 7 days. After 14–21 days, PBMC-NK cells were harvested and sorted using MACSQuant Tyto Cell Sorter, if necessary. The HLA genotypes, as well as the expression profiles of KIR and NKG family receptors in PBMCs, are summarized in Supplementary Table 12.

### Flow cytometry

The antibodies used in the flow cytometry are listed in Supplementary Table 7. An appropriate number of cells (1 × 10^4^ to 5 × 10^5^) were collected in a 96-well plate. Antibody was diluted with autoMACS Running Buffer–MACS Separation Buffer at 1–10 µg/mL, followed by adding it to the cells and incubating it at 4 °C for 30–60 min. Before analysis, 10 µg/mL 7-aminoactinomycin D (7-AAD) (Wako, 016-25241) was added. Stained cells were analyzed using MACSQuant Analyzer 10 (Miltenyi Biotec, 130-096-343) following the manufacturer’s instructions. FlowJo software (FlowJo LLC, Ver.10.7.1) was used for data analysis.

### Fluorescent cell sorting

Cells were stained similarly to the standard procedure in flow cytometry, after which 5 × 10^7^ cells/ml or less were loaded into MACSQuant Tyto Cartridges (130-106-088). Specific cell populations expressing proteins of interest were sorted using MACSQuant Tyto Cell Sorter (130-103-931) following the manufacturer’s instructions.

### NK cell cytotoxicity assay

Regarding effector NK cells, NK92 cells were stimulated with 500 IU/ml IL-2 from 24 h prior to the cytotoxicity assay. PBMC-NK cells were stimulated with 1000 IU/ml IL-2 from 48 h prior to the cytotoxicity assay. Regarding target cells, 500 µg/ml Recombinant Human IFN-g (Peprotech, 300-02) was added to the culture medium of iPSCs from 72 h prior to the cytotoxicity assay. The culture medium was changed to fresh medium containing 500 µg/ml IFN-g every 24 h. PBMC-T cells were stimulated with 100 IU/ml IL-2 from 24 h prior to the cytotoxicity assay.

Effector NK cells and target iPSCs or K562 cells were harvested and resuspended in fresh culture medium without cytokines. A total of 0.5–1 × 10^4^ iPSCs or K562 cells in 100 µl of culture medium were applied to a round-bottomed 96-well plate. The number of effector NK cells per well was calculated according to the effector target (ET) ratio. An appropriate number of NK cells in 100 µl of culture medium were applied to a 96-well plate and co-cultured with target cells at 37 °C under 5% CO_2_. Lirilumab IgG4 (Creative Biolabs, TAB-757) was added to the medium in soluble form for an activity assay. After incubation for 2.5–8 h, luciferase activity of each well was detected using a Luciferase Assay System (Promega, E6120), in accordance with the manufacturer’s instructions. The percentage of specific lysis was calculated as follows.

Specific lysis (%) = 100 × [(Basal luciferase activity of target cells only) − (Luciferase activity of target cells plus NK cells)] / (Basal luciferase activity of target cells only).

### CAR-T-cell cytotoxicity assay

PBMC-T cells expressing anti-human CD19 CAR scFv and/or KIR agonist scFv were obtained by lentiviral transduction and cell sorting. PBMC-NK cells were stimulated for an NK cell cytotoxicity assay. Human CD19 and GFP were introduced into Raji cells by lentiviral transduction to generate target cells for PBMC-T cells expressing anti-human CD19 CAR scFv. Effector PBMC-T cells and target Raji cells and PBMC-NK cells were harvested and resuspended in fresh culture medium without cytokines. A total of 1 × 10^4^ target Raji cells in 50 µl of culture medium were applied to a round-bottomed 96-well plate. The number of effector PBMC-T cells per well was calculated according to the ET ratio (1:1, 0.5:1, and 0.1:1). An appropriate number of PBMC-T cells in 50 µl of culture medium and 1 × 10^4^ PBMC-NK cells were applied to a 96-well plate and co-cultured with target cells at 37 °C under 5% CO_2_. After 4 days, the number of cells of each type in the wells was analyzed by flow cytometry. APC/Fire 750 anti-human CD3 antibody (Biolegend, 300470) was used to detect PBMC-T cells, and PE anti-human CD56 (Biolegend, 318306) was used to detect PBMC-NK cells. Raji cells were detected by GFP.

### Recombinant protein production

The recombinant proteins shown in Supplementary Table 8 were inserted into MCS of the pcDNA3.3 plasmid (Thermo Fisher Scientific, Cat# K830001), in accordance with the manufacturer’s instructions. The plasmids were transfected into FreeStyle 293-F (Thermo Fisher Scientific, Cat# R79007) using PEI-MAX (Polysciences, Cat#24765). To obtain biotinylated proteins, pcDNA plasmid containing the gene for biotin ligase A was co-expressed in 293-F cells or proteins were biotinylated using biotin ligase A expressed in *E. coli*. After purification, recombinant KIR proteins were purified from the culture supernatant by protein A affinity chromatography and desalting using HiTrap MabSelect SuRe (Cytiva, Cat# 29049104) and HiPrep 26/10 Desalting (Cytiva, Cat# 17-5087-01). Recombinant His-tagged scFv proteins were purified by Ni affinity chromatography and SEC chromatography using His Trap Excel (Cytiva, Cat# 17371205) and HiLoad 26/600 Superdex200pg (Cytiva, Cat# 28-9893-36). Recombinant Fab proteins were purified by affinity chromatography, desalting, and SEC chromatography using Kaneka KanCap G Prepacked Column (Kaneka, Cat# 111–01123), HiPrep 26/10 desalting, and HiLoad 26/600 Superdex200pg. The protein concentrations were detected using a Nanodrop spectrometer. Purity of the proteins was analyzed by HPLC-SEC and SDS-PAGE.

### Binding assay

Binding selectivity of KIR agonist, lirilumab, and PAN2D was analyzed using K562 cells expressing each scFv. A total of 1 × 10^4^ K562 cells were collected in a round-bottomed 96-well plate. Recombinant NK cell surface receptor-related proteins produced or obtained commercially (Supplementary Table 9) were diluted to 0.1 µM in autoMACS Running Buffer and applied to K562 cells. After incubation at 4 °C for 90 min, the cells were washed and APC-labeled anti-human IgG Fc antibody (Biolegend, 409306) diluted 1/50 in autoMACS Running Buffer was added. After incubation at 4 °C for 30 min, the cells were washed and analyzed by flow cytometry. Binding affinity to each protein was evaluated using median APC-derived fluorescence intensity. The intensity was normalized by that of K562 control cells.

### Phage panning

Phage display selection was performed using a human scFv phage library at Fair Journey Biologics (Iontas subsidiary, Cambridge, UK). To perform phage display selection, the panel of recombinant KIR proteins shown in Supplementary Table 10 was sourced (proteins were either purchased or prepared in house). Phage display selections were performed in solution phase using biotinylated recombinant proteins or non-biotinylated recombinant proteins, as shown in Supplementary Table 10. The solution phase phage display selection procedure was performed as described previously^59–62^. Briefly, the scFv-expressing phages that bind to the biotinylated KIR protein in solution were captured using streptavidin or neutravidin beads (positive selection). Subtractive selection to minimize binding to activating KIRs and enrich antibody clones specific for the targeted inhibitory KIR was performed using two methods. First, a molar excess of non-biotinylated activating KIR proteins was added to the selection mixture (competitive deselection). Second, the scFv-expressing phages that bind to the biotinylated activating KIR proteins were depleted prior to positive selection using streptavidin or neutravidin beads (depletion). In that manner, the scFv-expressing phages that specifically bind to the recombinant inhibitory KIR proteins are enriched between selection rounds. Following each round of phage display selection, the output titer was determined and binding activity against KIR proteins was assessed by polyclonal ELISA to evaluate the performance of the various selection conditions. After two rounds of selection, polyclonal phage populations enriched for inhibitory KIR-specific binders were obtained. Selected scFv sequences were sub-cloned from the phagemid to a recombinant protein expression vector^63^ and colonies were picked for monoclonal ELISA screening.

### ELISA

ELISA wells were coated with recombinant KIR proteins produced or commercially obtained (Supplementary Table 10). Flag-tagged scFv proteins selected from phage panning were applied to wells and detected by anti-flag antibody.

### Crystallization and structure determination

The purified KIR2DL2/3 agonist 61-Fab, KIR2DL2/3 agonist 61-scFv, or Pan2D-Fab was mixed with KIR2DL2 at a molar ratio of 1:1.2, and the complex was isolated by size exclusion chromatography using HiLoad 16/600 Superdex 200 pg (Cytiva, 28-9893-35).

KIR2DL2/3 agonist 61-Fab/KIR2DL2 complex was concentrated to 19 mg/mL and crystallized by the vapor diffusion method. The complex was mixed with crystallization solution containing 10% (w/v) polyethylene glycol 3,350 (Hampton Research, HR2-527), 0.2 M calcium acetate, and 0.1 M imidazole-hydrochloric acid pH 7.5 at a 1:1 ratio and incubated for about 3 days at 20 °C. The obtained crystals were cryoprotected by increasing the concentration of polyethylene glycol 3350 to 35%, and then frozen in liquid nitrogen. The diffraction images were collected at 95 K using beamline BL-17 A at the Photon Factory.

KIR2DL2/3 agonist 61-scFv/KIR2DL2 was concentrated to 14 mg/mL, crystallized by mixing with crystallization solution containing 0.1 M Bis-Tris propane pH 7.0 and 29.0% Tacsimate pH 7.0 (Hampton Research, HR2-755) at a 1:1 ratio, and incubated for about 3 days at 20 °C. The diffraction images were collected at 100 K using beamline X06SA at Swiss Light Source.

Pan2D-Fab/KIR2DL2 was concentrated to 9.6 mg/mL and crystallized by mixing with crystallization solution containing 19.0% (w/v) polyethylene glycol 3350, 0.2 M sodium malonate pH 5.0 at a 1:1 ratio, and incubated for about 20 days at 20 °C. The diffraction images were collected at 95 K using beamline BL-17 A at the Photon Factory.

The diffraction images were indexed, integrated with XDS^64^and scaled with Aimless^65^. The software package PHENIX^66^ was used for structural analysis calculations. The initial phases were determined by molecular replacement using Phaser^67^ with the KIR2DL2 structure (PDB code: 2DL2) and the IgG Fab structure (PDB code: 1MIM) as search models. The atomic models were modified manually by Coot^68^ and refined by phenix.refine^69^. This procedure was repeated until the calculations converged. The data collection and refinement statistics for the final models are shown in Supplementary Table 11.

#### Molecular modeling

Homology modeling of KIR2DL2/DL3/2DS1/2DS2/2DS4 was performed using Prime Version 6.3 software. Crystal structures of KIR2DL2/DL3 in the Fab-bound state were used as the template structures. The structure of KIR2DL3 bound to lirilumab-Fab (PDB code: 8TUI) was used in this process. Using the homology model that we constructed, we focused on amino acids that differed from the template structure and conducted an analysis using Maestro Version 12.7 software to determine whether the interactions could be maintained.

### In vivo reactivity with allogeneic anti-CD19 CAR-T and PBMC-NK cells

All animal experiments were approved by Daiichi Sankyo Animal Care and Use Committee, in accordance with the standards of the Association for Assessment and Accreditation of Laboratory Animal Care (AAALAC). We also confirmed that our study met the criteria of ARRIVE2.0 guideline. Human PBMC-NK cell transplantation to mice was based on a previous report^70^. PBMC-T cells and PBMC-NK cells were prepared as described for the CAR-T-cell cytotoxicity assay. A total of 1 × 10^7^ PBMC-NK cells expanded in feeder-free conditions for 14 days were transplanted intravenously into 9-week-old male human IL-15-transgenic NOG mice purchased from In-Vivo Science Inc. Seventeen days later, 2 × 10^6^ PBMC-T cells were transplanted intravenously and blood samples obtained from the tail vein were analyzed by flow cytometry 4 and 7 days later.

#### Statistics

All data are expressed as mean ± standard deviation (s.d.). All statistical analyses were performed in Prism 9.1.0 for Windows (GraphPad Software, San Diego, CA, USA; www.graphpad.com). One-way analysis of variance (ANOVA) with Tukey’s post hoc test was used for the comparison of three or more groups in a single condition. Two-way ANOVA with Sidak’s or Tukey’s multiple testing correction was used for the comparison of three or more groups in multiple tests. Technical replicate numbers are indicated in each in vitro study and biological replicate number is indicated in in vivo study.

## Supplementary Information

Below is the link to the electronic supplementary material.

Supplementary Material 1

Supplementary Material 2

Supplementary Material 3

Supplementary Material 4

## Data Availability

All data supporting the results are available in the main text or the supplementary materials. The source data for the main figures and extended data figures are provided as Source Data files. The DNA sequences of the vectors and plasmids used in this study are available upon corresponding author reasonable request. The crystal structures of KIR agonists in complex with KIR2DL2 have been deposited to the Protein data Bank (www.pdb.org) under the accession number (9LRF: https://doi.org/10.2210/pdb9LRF/pdb, 9LRH: https://doi.org/10.2210/pdb9LRH/pdb, 9LRA: https://doi.org/10.2210/pdb9LRA/pdb, Supplementary Table 11).
