# Supplementary material for: Selective agonists of KIR and NKG2A to evade missing self response of natural killer cells
Source: Sci Rep. 2025 Sep 29;15:33550. doi: 10.1038/s41598-025-18394-z (PMC12480839; doi:10.1038/s41598-025-18394-z)
Supplement: Supplementary file 1 — Supplementary Material 1 [file 41598_2025_18394_MOESM1_ESM.docx]

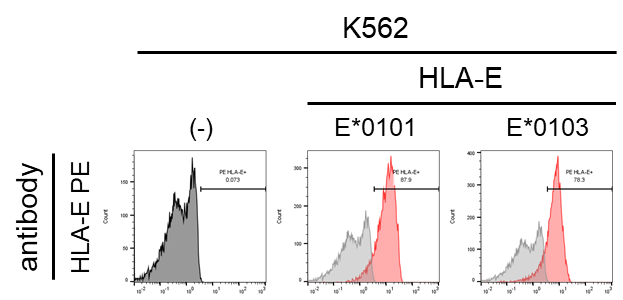


Supplementary Figure 1. HLA-E chimera protein expression on K562 cells.

The expression of HLA-G peptide, B2M, HLA-E chimera protein on K562 cells was detected by flow cytometry and compared to control K562 (−) cells (gray). The HLA-E alleles are indicated above each sample.


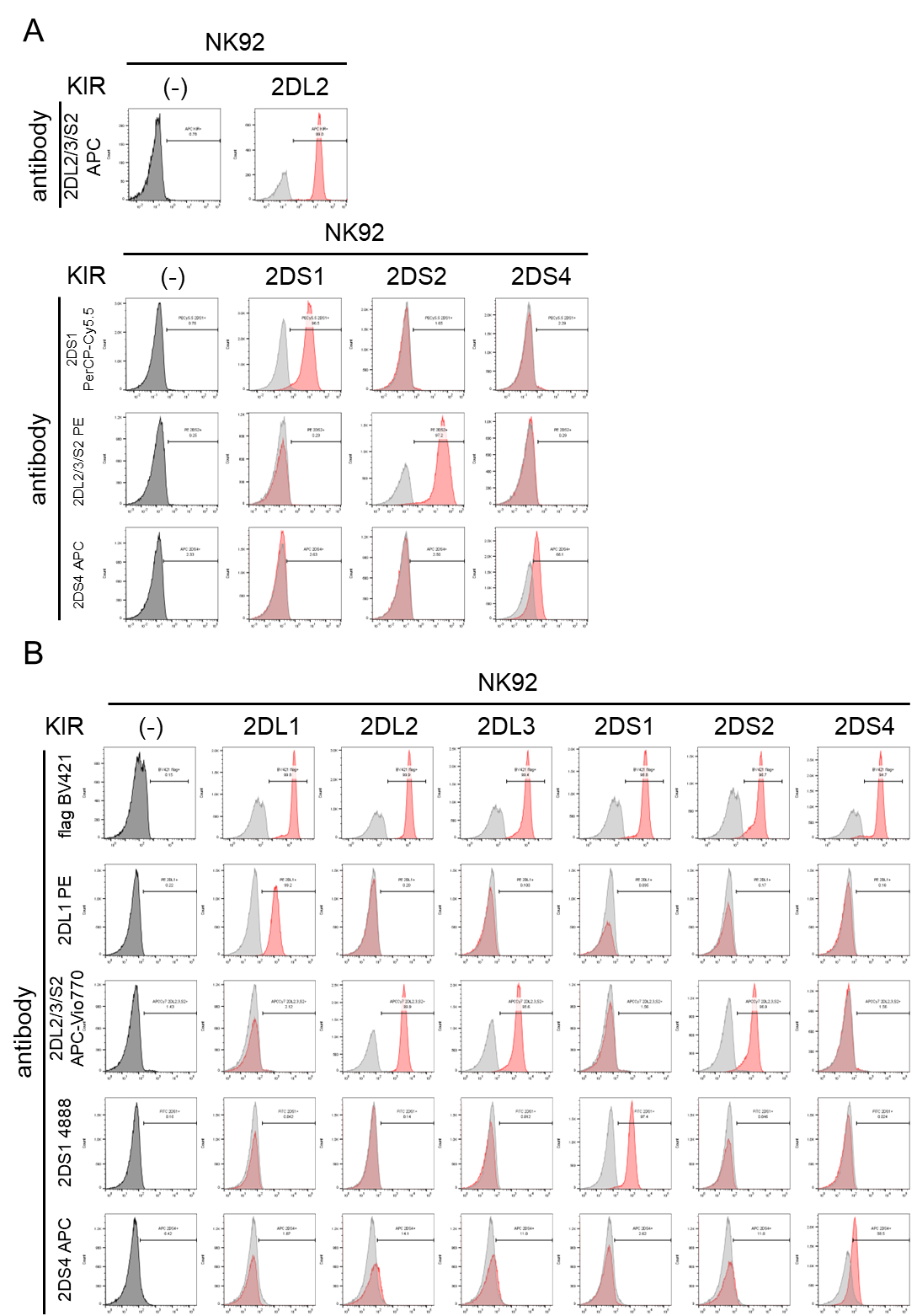


Supplementary Figure 2. KIR expression on NK-92 cells.

The expression of KIR family members on NK-92 cells was detected by flow cytometry and compared to control NK-92 (−) cells (gray). A) KIRs without tag are shown. The KIR alleles KIR2DL2*003, KIR2DS1*002, KIR2DS2*001, and KIR2DS4*001 were used. B) KIRs with flag tag are shown. The KIR alleles KIR2DL1*002, KIR2DL2*001, KIR2DL3*001, KIR2DS1*002, KIR2DS2*001, and KIR2DS4*001 were used.


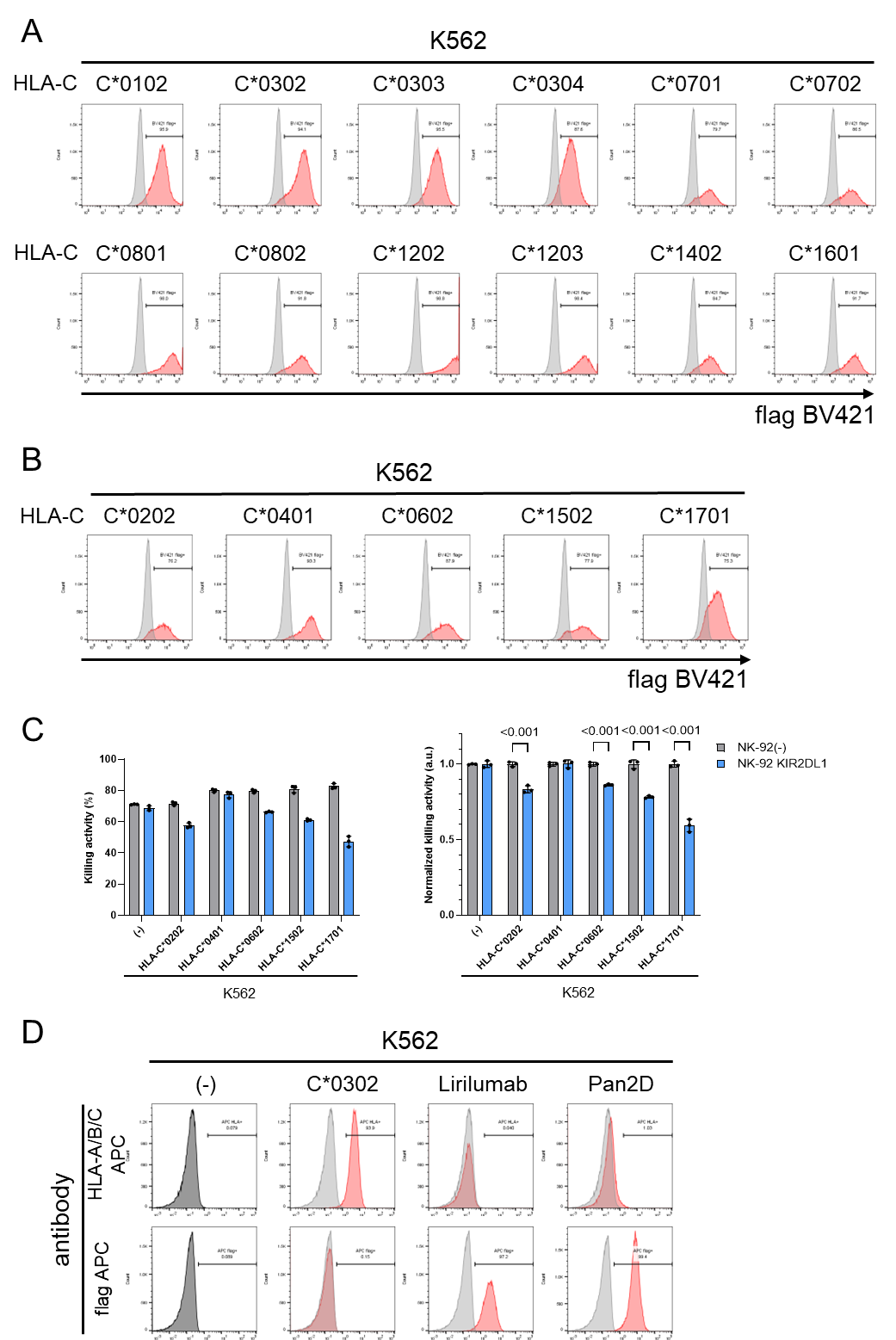


Supplementary Figure 3. B2M, HLA-C chimera protein expression on K562 cells and suppression of KIR2DL1 expressing NK-92 cells by HLA-C2.

The expression of flag, B2M, HLA-C1 chimera protein (A) and flag, B2M, HLA-C2 chimera protein (B) on K562 cells was detected by flow cytometry and compared to K562 WT cells (gray). The HLA-C alleles are indicated above each sample. A target of antibody was presented at the tips of the arrows. C) NK-92 cells and K562 cells were co-cultured for 3 h (E:T = 1:1). The KIR allele KIR2DL1*002 was used. Means and SDs of the dead K562 cell ratio (left) and the ratio normalized against the control (right) are shown (n=3). p-values obtained by two-way ANOVA with Šídák’s multiple comparison test are presented. Source data are provided as a Source Data file. D) The expression of the sole HLA-C*0302 or membrane bound anti-KIR antibody (Lirilumab, Pan2D) scFv consists of CD8 long hinge was detected by flow cytometry and compared to control K562 (−) cells (gray).


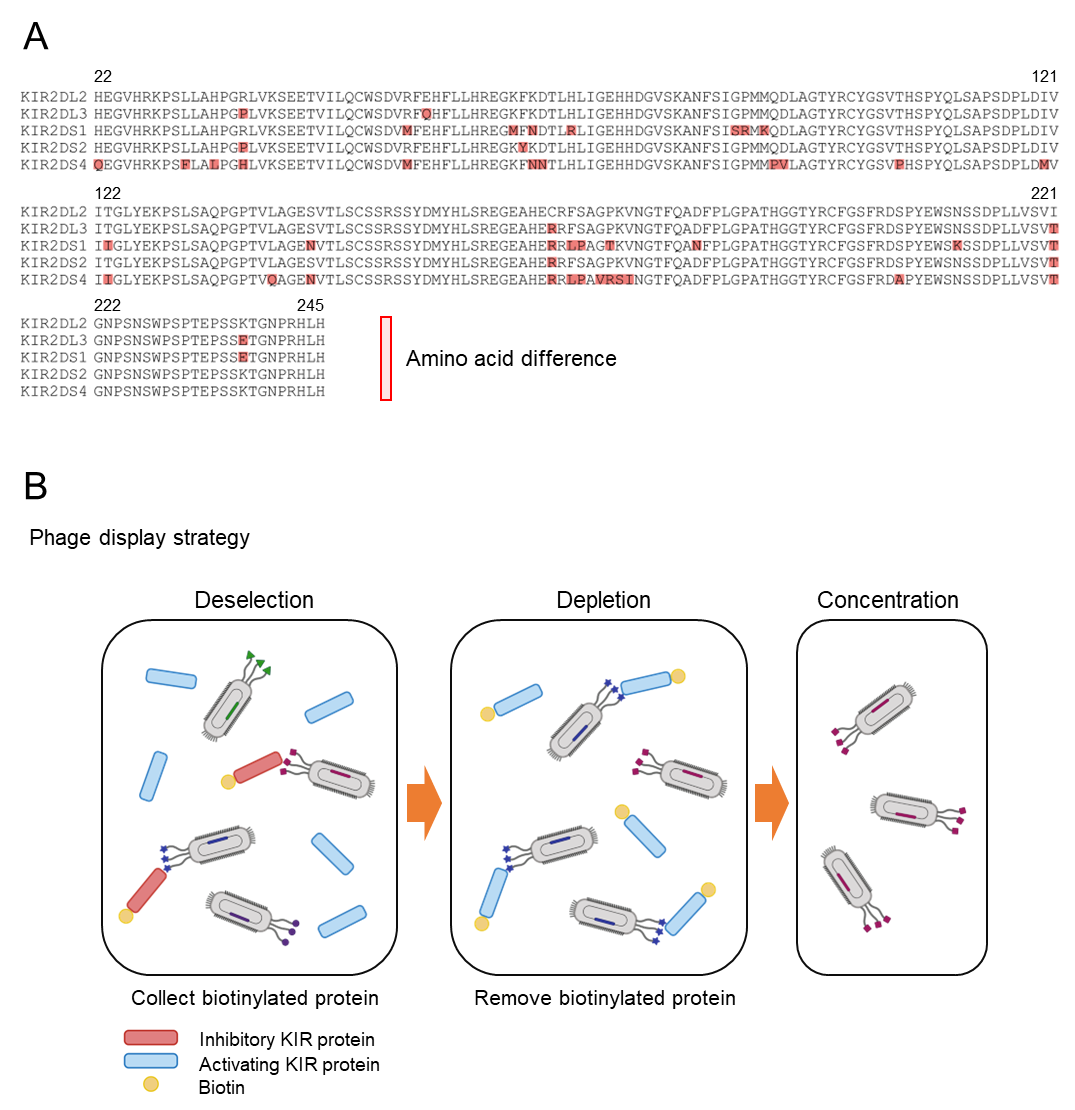


Supplementary Figure 4. Alignment of amino acid sequences of KIR family members based on KIR2DL2 and the strategy of phage display

A) Amino acid sequences of KIR family members were aligned. Amino acids differing from those of KIR2DL2 in other KIR family members are highlighted in red. The KIR alleles KIR2DL2*001, KIR2DL3*001, KIR2DS1*002, KIR2DS2*001, and KIR2DS4*001 were used. B) Schematic overview of phage display was illustrated. Red blocks indicate inhibitory KIR proteins and blue blocks indicate activating KIR proteins. Yellow circles indicate biotins. During the deselection phase, biotinylated inhibitory KIR proteins and the phages that bound to them (red phages and blue phages) were collected. During the depletion phase, biotinylated activating KIR proteins and the phages that bound to them (blue phages) were removed.


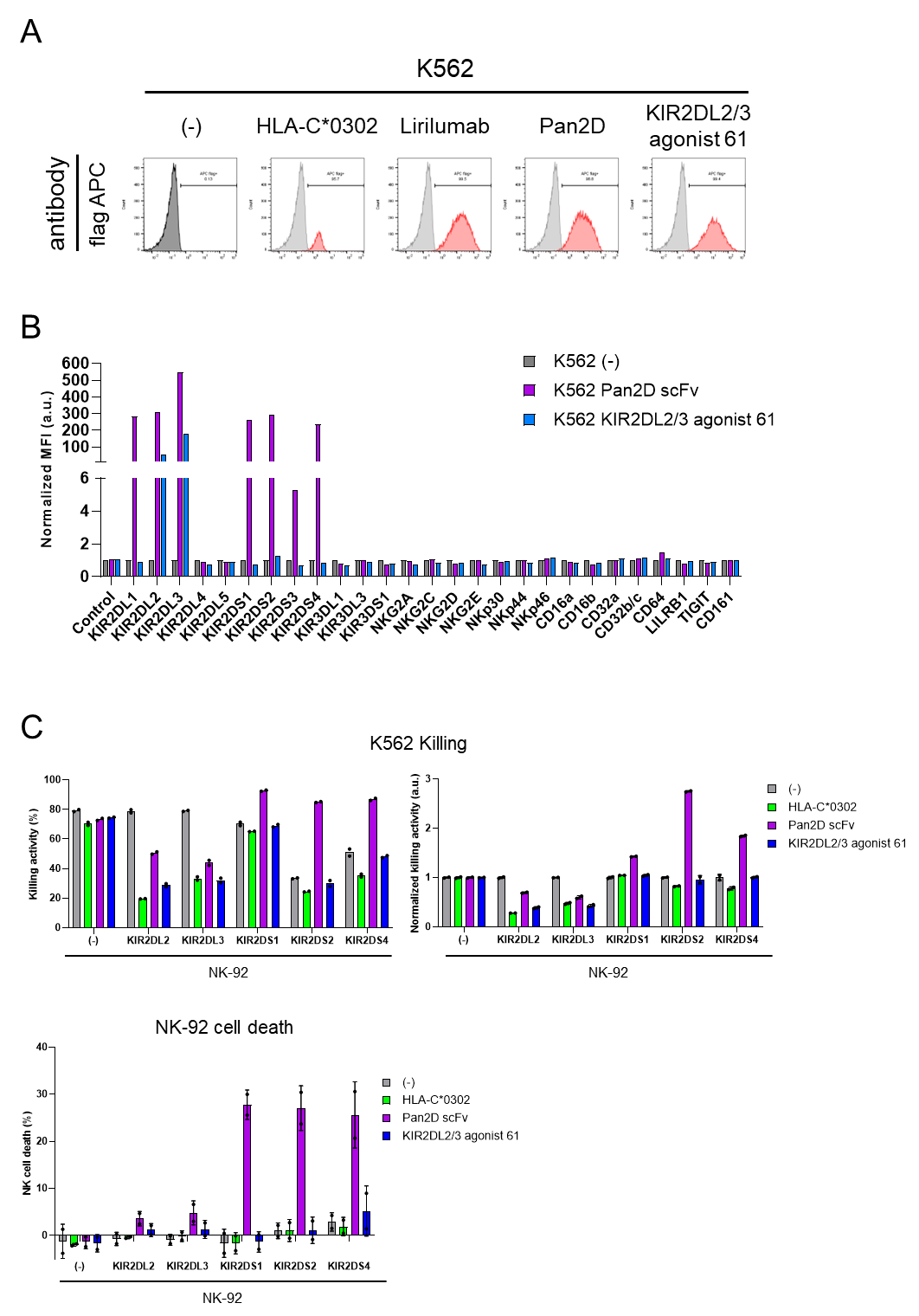


Supplementary Figure 5. The KIR2DL2/3 agonist 61 selectively bound to inhibitory KIR rather than other receptors on NK cells and did not harm NK cells expressing activating KIRs.

A) The expression of the sole HLA-C*0302 or the membrane bound anti-KIR antibody (Lirilumab, Pan2D, KIR2DL2/3 agonist 61) scFvs was detected by flow cytometry and compared to control K562 (−) cells (gray). B) The binding affinity of recombinant proteins to membrane bound anti-KIR antibody scFv expressed on K562 cells was assessed by flow cytometry (n=1). The mean fluorescent intensity of each protein was normalized against that of control K562 cells. Information on the recombinant proteins is provided in Table5. C) Chelate-labeled NK-92 cells and K562 cells were co-cultured for 3 h (E:T = 1:1). The KIR alleles KIR2DL2*001, KIR2DL3*001, KIR2DS1*002, KIR2DS2*001, and KIR2DS4*001 were used. Means and SDs of the dead K562 cell ratio (left) and the ratio normalized against the control (right) are shown (n=2). Means and SDs of the dead NK-92 cell ratio (below) was calculated using the leaked chelate concentration in the supernatant. Source data are provided as a Source Data file.


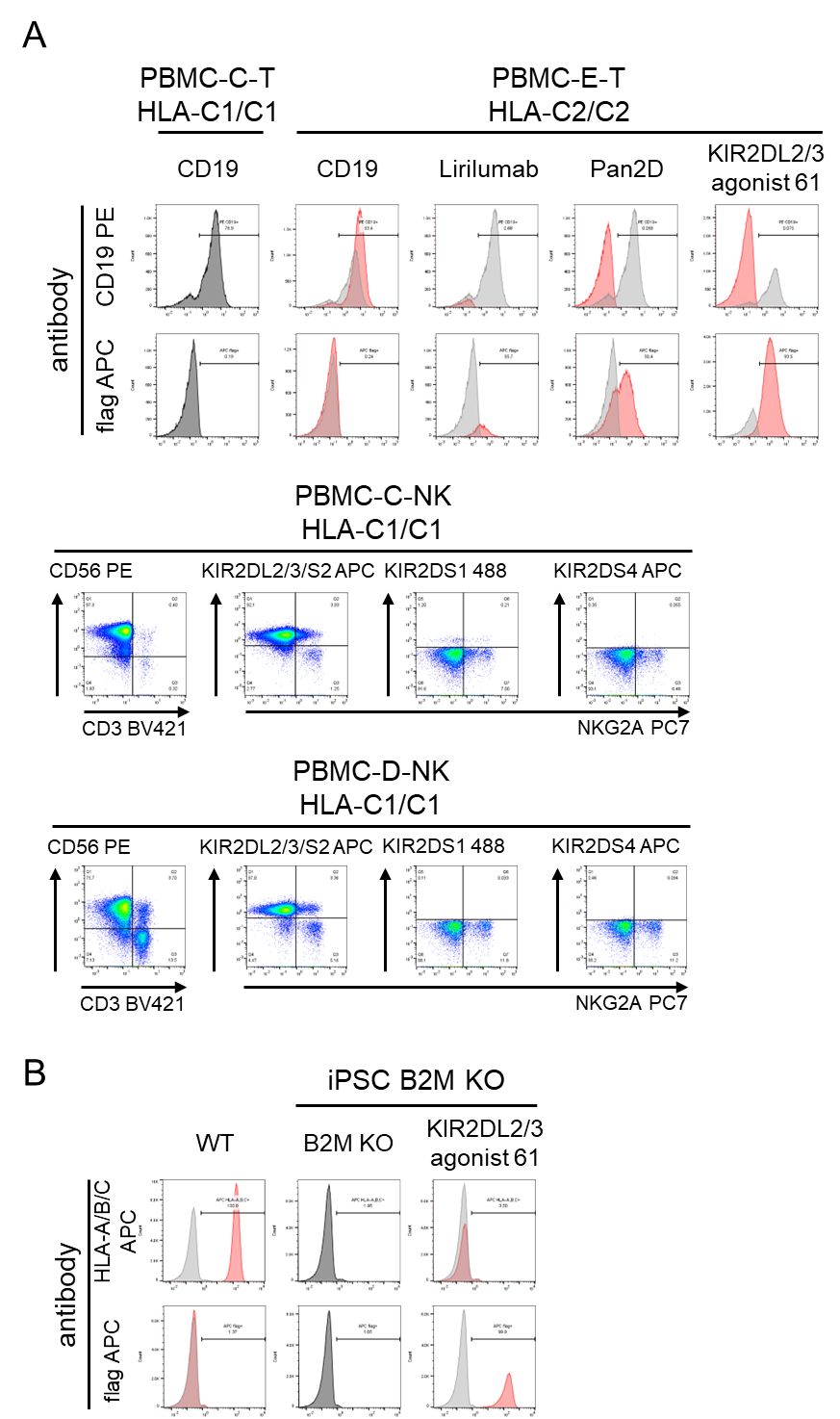


Supplementary Figure 6. Expression of KIR families on PBMC-derived NK cells and membrane bound anti-KIR antibody scFvs on PBMC-derived T cells or iPSCs.

A) The expression of control truncated CD19 or the membrane bound anti-KIR antibody (Lirilumab, Pan2D, KIR2DL2/3 agonist 61) scFvs on PBMC-derived T cells was detected by flow cytometry (top) and compared to PBMC-C-T cells (gray). KIR family members and NKG2A expression on CD3-/CD56+ PBMC-derived NK cells were detected by flow cytometry (below). PBMC-derived NK cells were expanded using anti-CD16 antibody. The PBMC-C and PBMC-D carries HLA-C1/C1 and the PBMC-E carries HLA-C2/C2. B) The expression of endogenous HLA-A/B/C or membrane bound KIR2DL2/3 agonist 61 scFv on iPSCs was detected by flow cytometry and compared to B2M KO iPSCs (gray).


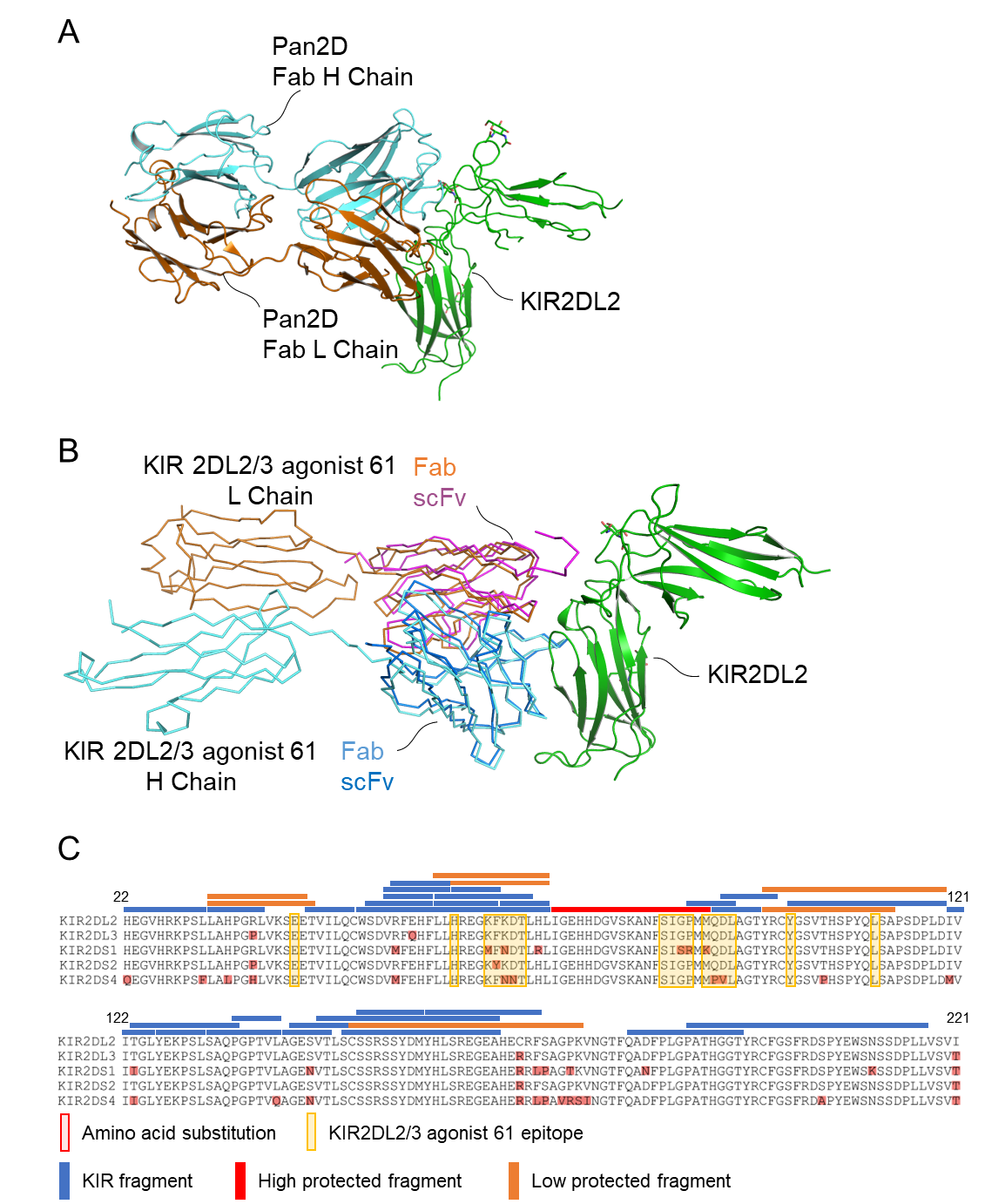


Supplementary Figure 7. The ribbon diagrams of the anti-KIR antibodies-KIR2DL2 complex and epitope prediction by HDX-MS analysis.

A) The complex of ribbon diagrams representing Pan2D Fab and KIR2DL2 determined using the X-ray crystal structure analysis is shown. The green ribbon indicates KIR2DL2. The orange and cyan ribbons indicate Pan2D Fab L chain and H chain, respectively. B) The overlay of ribbon diagrams of KIR2DL2/3 agonist 61 Fab and scFv against KIR2DL2. The green ribbon indicates KIR2DL2. The orange and purple ribbons indicate Fab L chain and scFv L chain, respectively. The cyan and blue ribbons indicate Fab H chain and scFv H chain, respectively. C) The amino acid sequences of KIR family members were aligned, and KIR fragments used in HDX-MS analysis are shown above the alignment as colored bars. The red bar indicates a fragment strongly protected from hydrogen-deuterium exchange, and the orange bars indicate weekly protected fragments. The blue bars indicate fragments not protected from hydrogen-deuterium exchange. Amino acids differing from those of KIR2DL2 in other KIR family members are highlighted in red. The epitopes of KIR2DL2/3 agonist 61 against KIR2DL2 determined by X-ray crystal structure analysis is highlighted as yellow boxes in the sequence alignment. The KIR allele KIR2DL2*001 was used for X-ray crystal structure analysis and HDX-MS analysis.


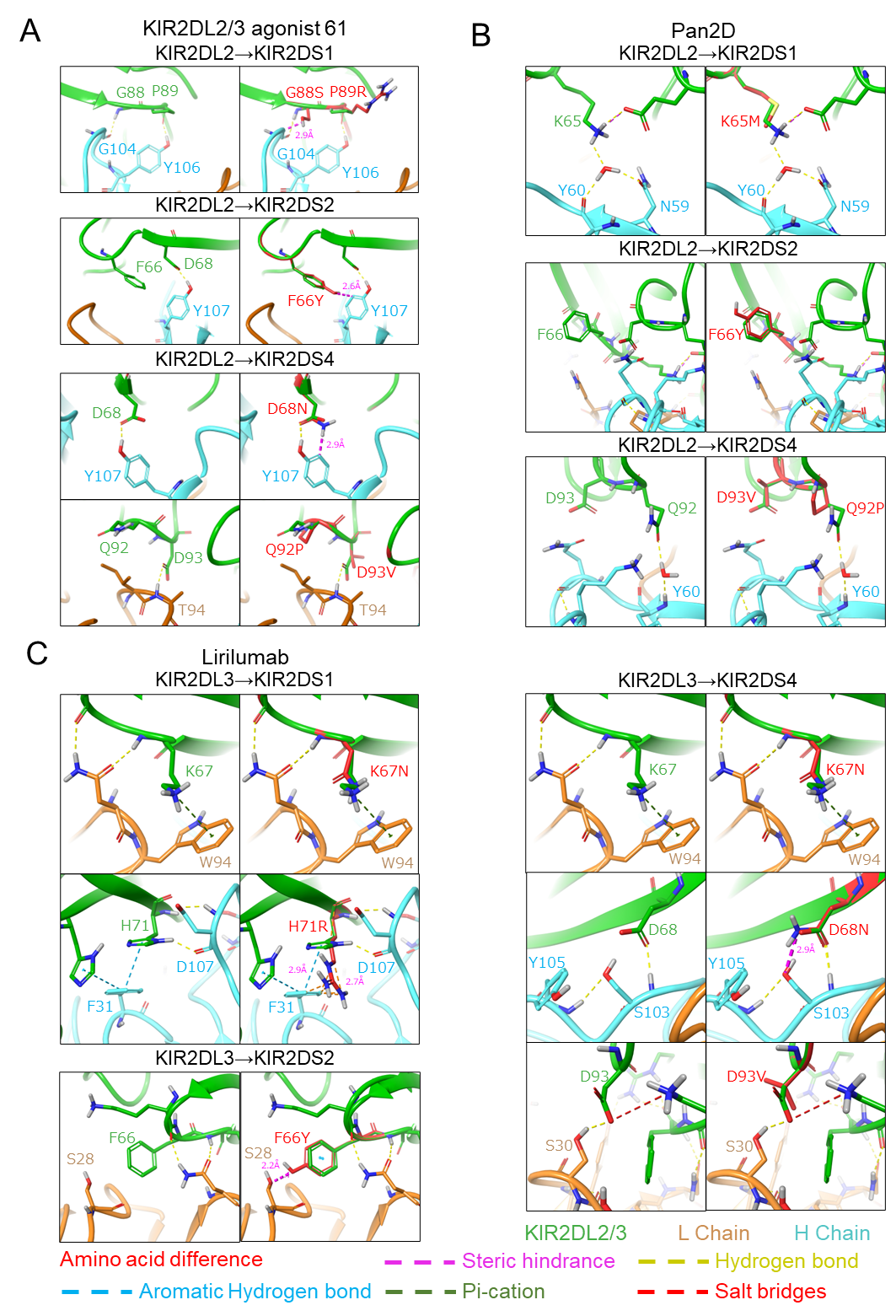


Supplementary Figure 8. Homology models of anti-KIR antibodies to KIR2DL2 complex against activating KIRs.

The left panel illustrates the interactions between anti-KIR antibodies and KIR2DL2 or KIR2DL3, and the right panel illustrates the interactions between anti-KIR antibodies and activating KIRs at the same site. A) The green ribbon indicates KIR2DL2. The orange and cyan ribbons indicate KIR2DL2/3 agonist 61 Fab L chain and H chain, respectively. B) The green ribbon indicates KIR2DL2. The orange and cyan ribbons indicate Pan2D Fab L chain and H chain, respectively. C) The green ribbon indicates KIR2DL3. The orange and cyan ribbons indicate Lirilumab Fab L chain and H chain, respectively. The amino acids of each protein are colored to match the color of protein ribbon. The amino acids on activating KIRs that differ from KIR2DL2 are presented in red. The pink dashed lines indicate the steric hindrance, and the distances between molecules are presented in pink. The yellow dashed lines indicate hydrogen bonds. The cyan dashed lines indicate aromatic hydrogen bonds. The green dashed lines indicate cation-Pi interactions. The red dashed lines indicate salt bridges. The KIR alleles KIR2DL2*001, KIR2DL3*001, KIR2DS1*002, KIR2DS2*001, and KIR2DS4*001 were used for homology modeling.


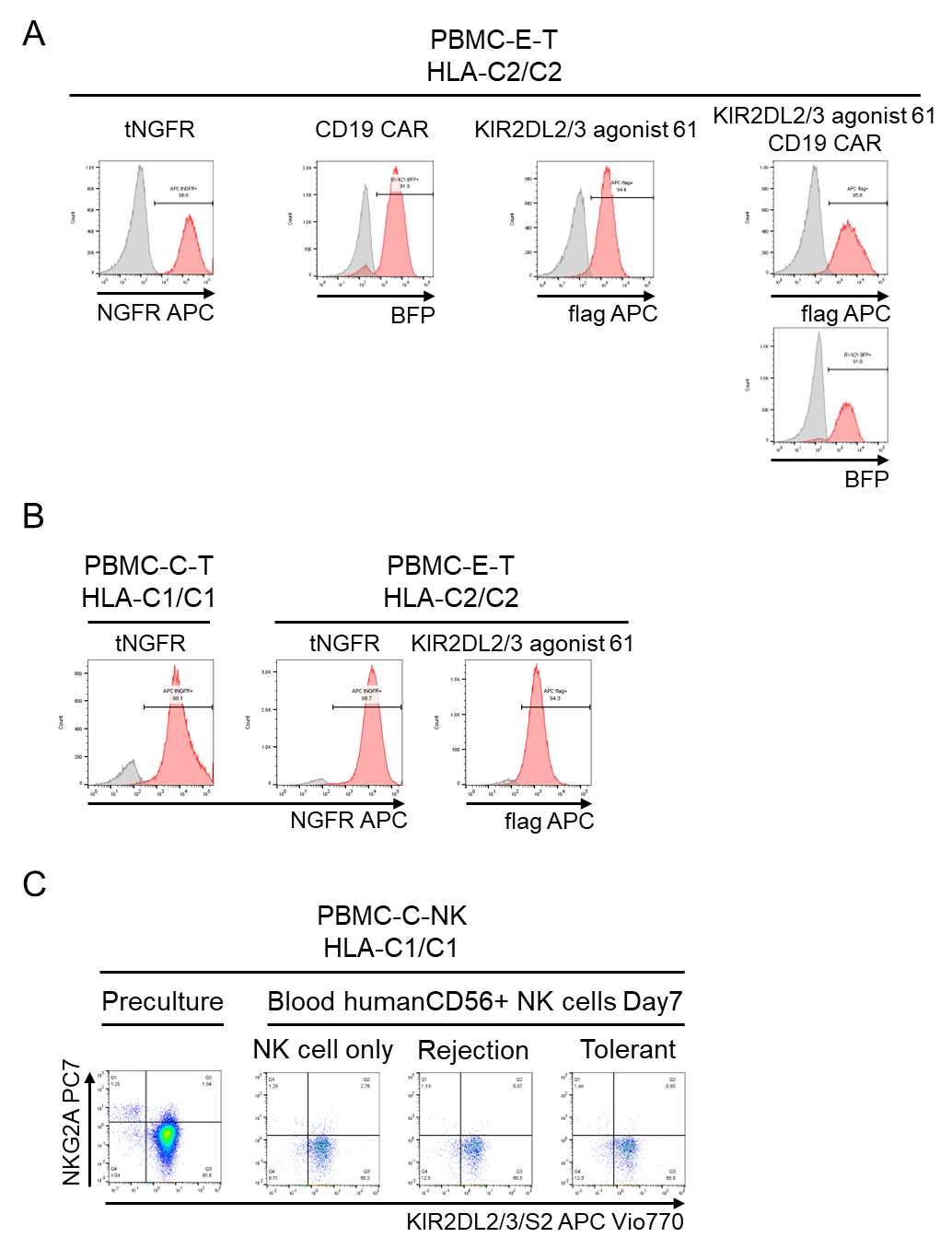


Supplementary Figure 9. The surface expression of PBMC-derived T cells and PBMC-derived NK cells used for in vitro and in vivo studies.

A) The expression of control truncated NGFR or the membrane bound KIR2DL2/3 agonist 61 scFv or anti-CD19 CAR on PBMC-E-derived T cells was detected by flow cytometry and compared to control untransduced PBMC-E-derived T cells (gray). The BFP was expressed downstream of anti-CD19 CAR using the IRES sequence to label the anti-CD19 CAR expressing cells. The PBMC-E carries HLA-C2/C2. B) The expression of control truncated NGFR or the membrane bound KIR2DL2/3 agonist 61 scFv on PBMC-derived T cells was detected by flow cytometry and compared to control untransduced PBMC-E-derived T cells (left and center panel, gray) or PBMC-C-derived T cells (right panel, gray). The PBMC-C carries HLA-C1/C1 and the PBMC-E carries HLA-C2/C2. C) NKG2A and KIR2DL2/3, 2DS2 expression on PBMC-C-derived NK cells was detected by flow cytometry. PBMC-derived NK cells were expanded using anti-CD16 antibody. The human CD56 positive cells in mouse peripheral blood ware treated as human PBMC-derived NK cells after in vivo transplantation at day 7. The rejection condition means PBMC-C derived NK cells with control PBMC-E derived T cells, and the tolerant condition means PBMC-C derived NK cells with KIR2DL2/3 agonist 61 expressing PBMC-E derived T cells.


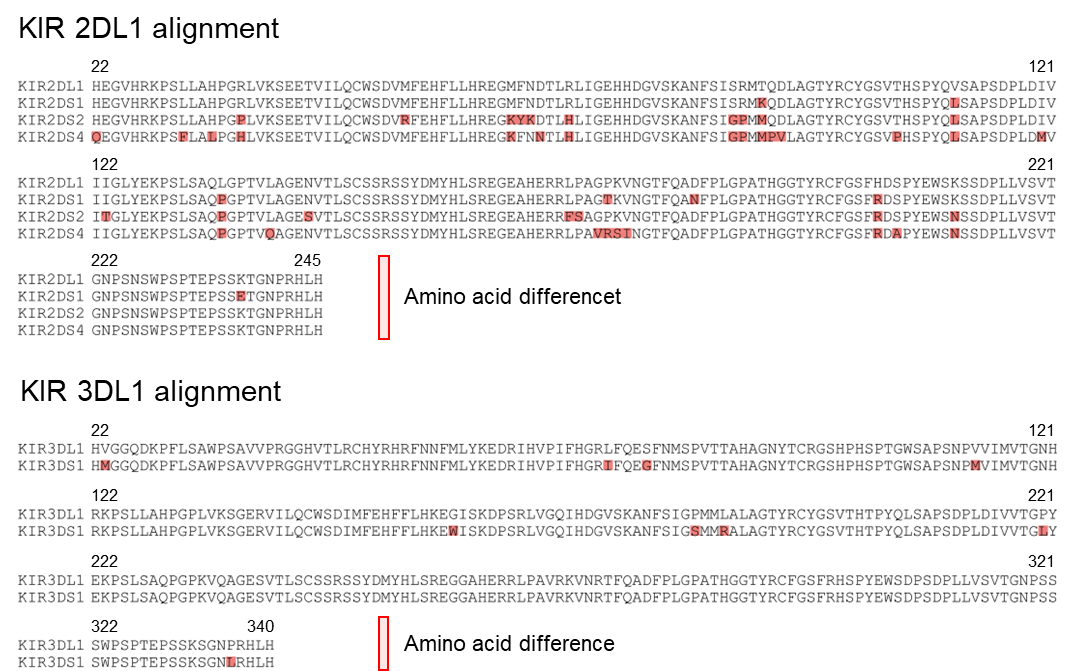


Supplementary Figure 10. Alignment of amino acid sequences of KIR family members based on KIR2DL1 and KIR3DL1.

Amino acid sequences of the KIR family members were aligned. Amino acids differing from those of KIR2DL1 or KIR3DL1 in other KIR family members are highlighted in red. The KIR alleles KIR2DL1*002, KIR2DS1*002, KIR2DS2*001, KIR2DS4*001, KIR3DL1*015, and KIR3DS1*011 were used.


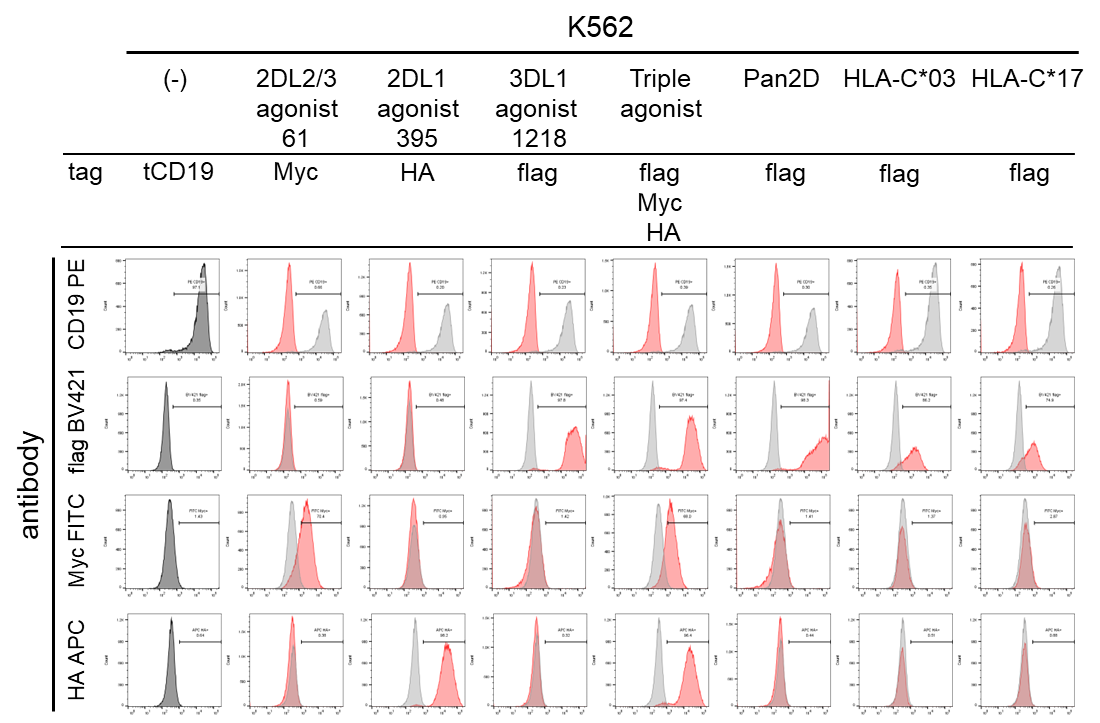


Supplementary Figure 11. Introduction of triple novel KIR agonists to K562 cells.

The expression of control truncated CD19, B2M-HLA-C*0302 chimera protein, B2M-HLA-C*1701 chimera protein and membrane bound anti-KIR antibody (Pan2D, KIR2DL2/3 agonist 61, KIR2DL1 agonist 395, KIR3DL1 agonist 1218) scFvs on K562 cells was detected by flow cytometry and compared to control tCD19 expressing K562 (−) cells (gray). The KIR2DL2/3 agonist 61 was tagged with Myc. The KIR2DL1 agonist 395 was tagged with HA. The KIR3DL1 agonist 1295, Pan2D scFv, B2M-HLA-C*0302 or C*1701 chimera proteins were tagged with flag.


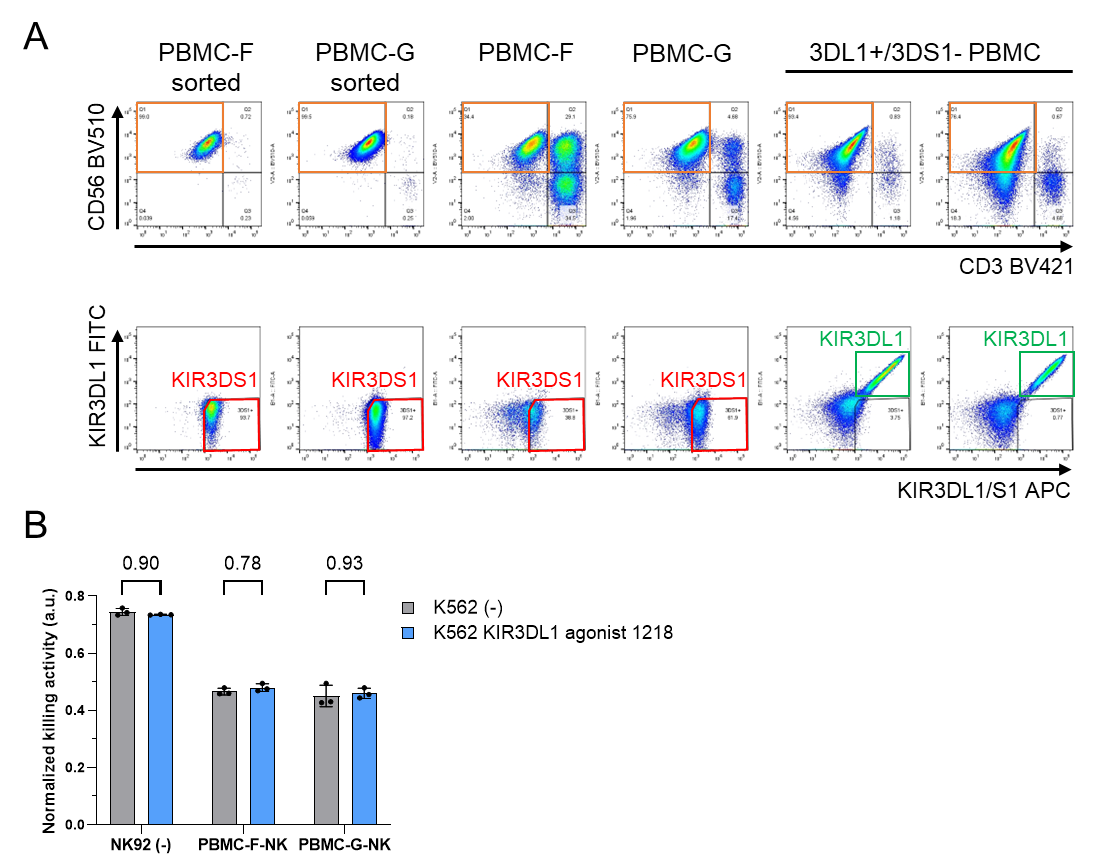


Supplementary Figure 12. The KIR3DL1 agonist 1218 did not activate KIR3DS1 expressing PBMC-derived NK cells.

A) The expression of KIR3DS1 on PBMC-derived NK cells was detected by flow cytometry using a combination of anti-KIR3DL1 and anti-KIR3DL1/3DS1 antibodies. CD3-/CD56+ cells were treated as NK cells (orange frame). The KIR3DS1+ cells are indicated in red frames, and the KIR3DL1+ cells in green frames. Sorted samples of PBMC-F and PBMC-G indicate CD3-/CD56+, KIR3DS1+ cells obtained by using MACSQuant Tyto cell sorter. The control KIR3DS1 negative PBMC-derived NK cells were obtained from PBMCs genetically lacking KIR3DS1. PBMC-derived NK cells were expanded using feeder K562 cells expressing membrane-bound IL-21 and HLA-G peptide, B2M and HLA-E chimeric protein. B) PBMC derived NK cells and K562 cells were co-cultured for 3 h (E:T = 3:1). Means and SDs of the dead K562 cell ratio are shown (n=3). p-values obtained by two-way ANOVA with Šídák’s multiple comparison test are indicated. Source data are provided as a Source Data file.


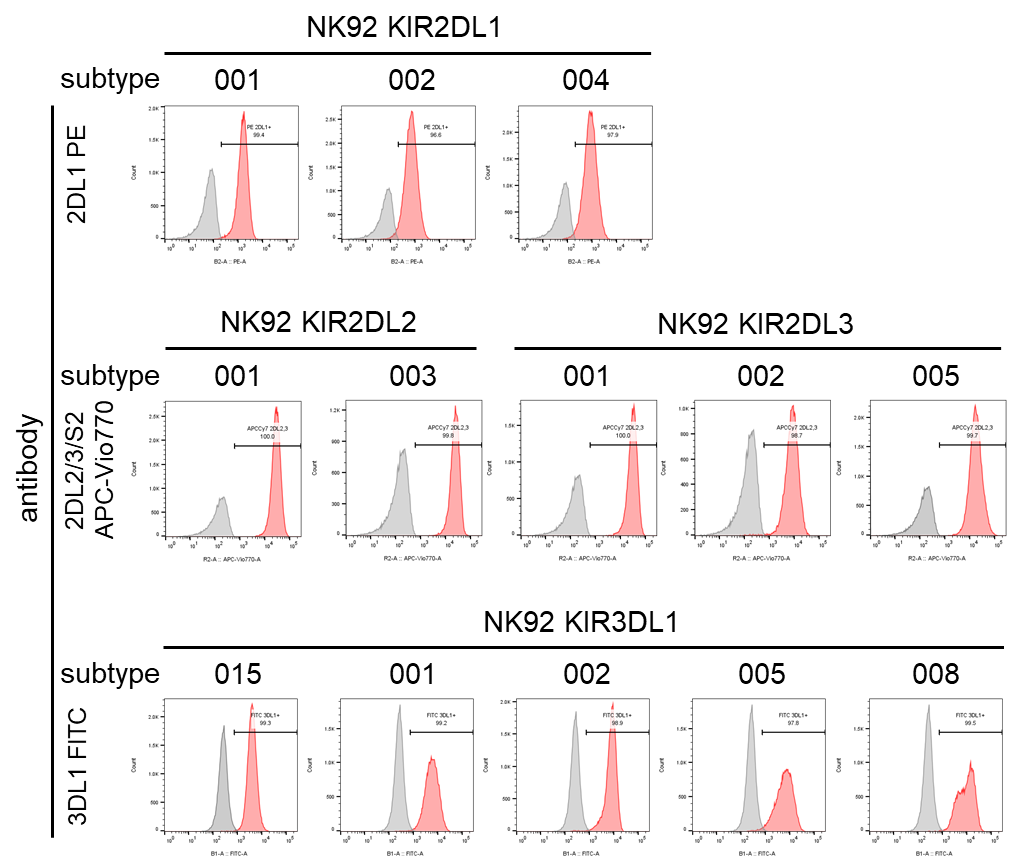


Supplementary Figure 13. Expression of subtypes of KIR family members on NK-92 cells.

The expression of subtypes of KIR family members on NK-92 cells was detected by flow cytometry and compared to control NK-92 (−) cells (gray). Each KIR allele is indicated above the samples.


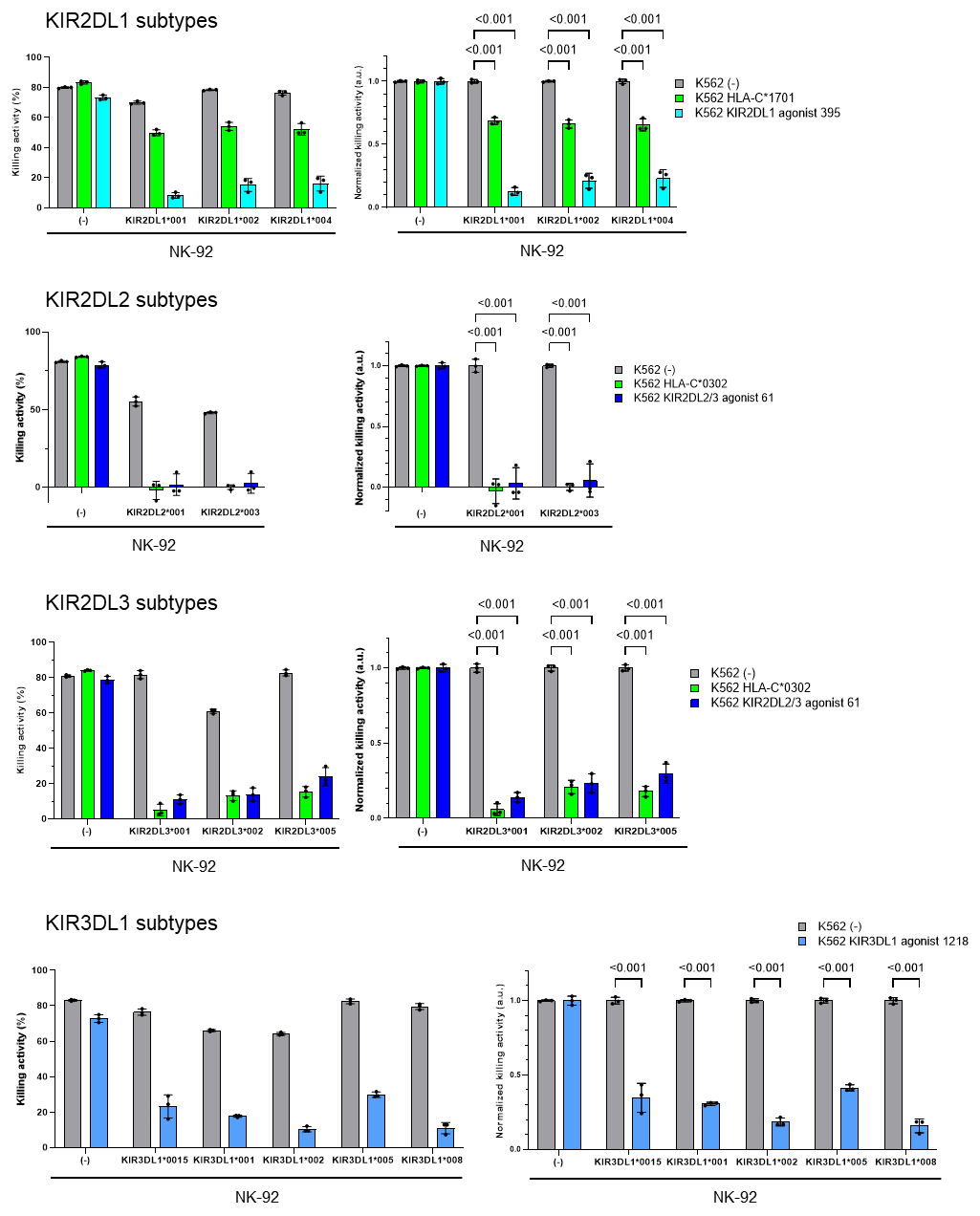


Supplementary Figure 14. Each novel KIR agonist suppressed the cytotoxicity of NK-92 cells expressing the subtypes of KIR family members.

NK-92 cells and K562 cells were co-cultured for 3 h (E:T = 1:1). Means and SDs of the dead K562 cell ratio (left) and the ratio normalized against the control (right) are shown (n=3). p-values obtained by two-way ANOVA with Tukey’s multiple testing correction (for KIR2DL1, KIR2DL2, KIR2DL3) and Šídák’s multiple comparison test (for KIR3DL1) are indicated. Source data are provided as a Source Data file.


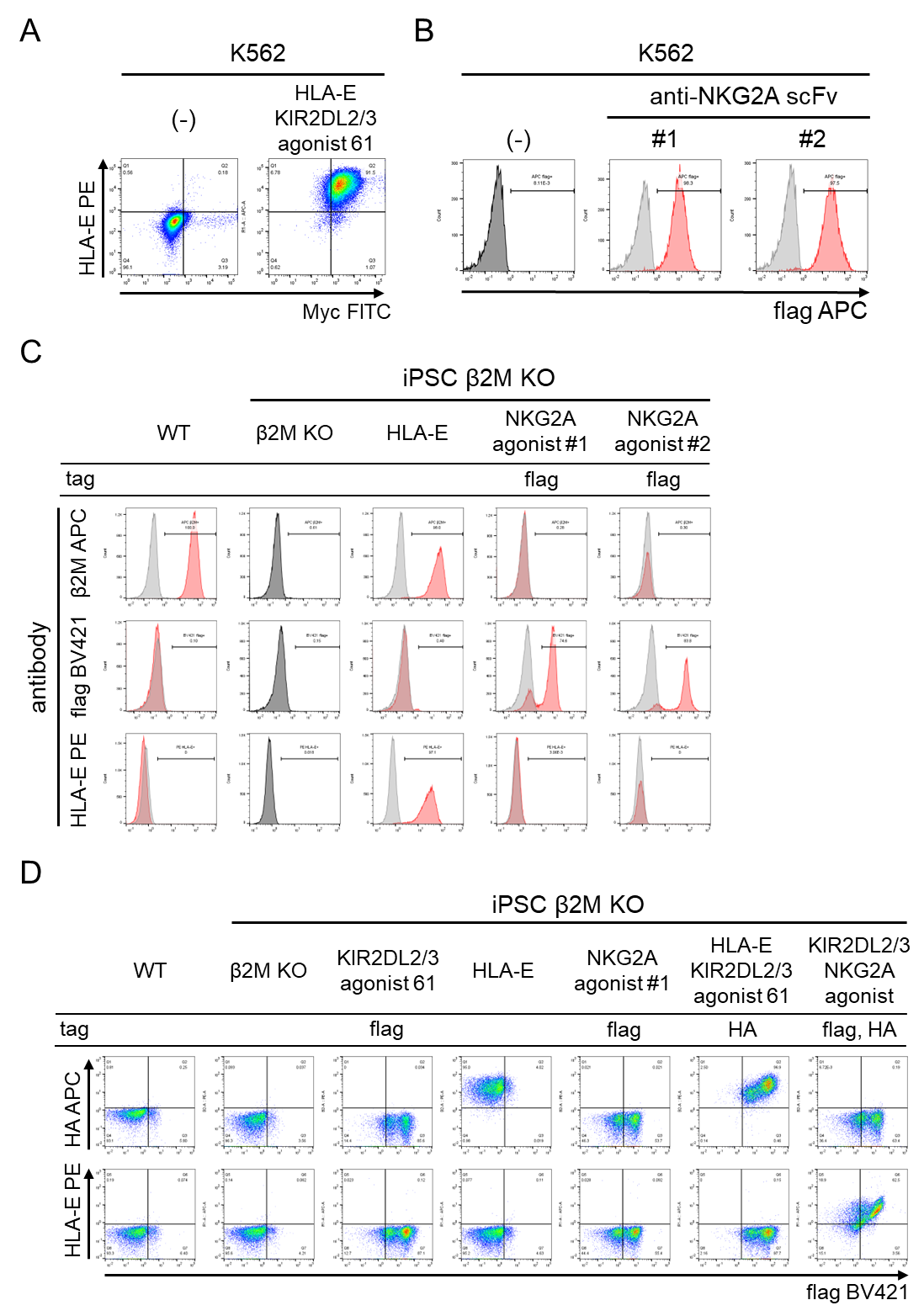


Supplementary Figure 15. The expression of HLA-E and membrane bound anti-NKG2A scFv in combination with KIR2DL2/3 agonist 61 on K562 cells and iPSCs.

A) The expression of HLA-G peptide, B2M, HLA-E chimera protein in combination with KIR2DL2/3 agonist 61 on K562 cells was detected by flow cytometry. HLA-E*0101 allele was used. B) The expression of membrane bound anti-NKG2A scFv on K562 cells was detected by flow cytometry and compared to control K562 (−) cells (gray). C) The expression of the endogenous HLA-A/B/C, HLA-G peptide, B2M, HLA-E chimera protein and membrane bound anti-NKG2A scFv on iPSCs were detected by flow cytometry and compared to B2M KO iPSCs (gray). D) The expression of the endogenous HLA-A/B/C, HLA-G peptide, B2M, HLA-E chimera protein and membrane bound anti-NKG2A scFv in combination with KIR2DL2/3 agonist 61 on iPSCs was detected by flow cytometry. The KIR2DL2/3 agonist 61 was tagged with HA for combination.


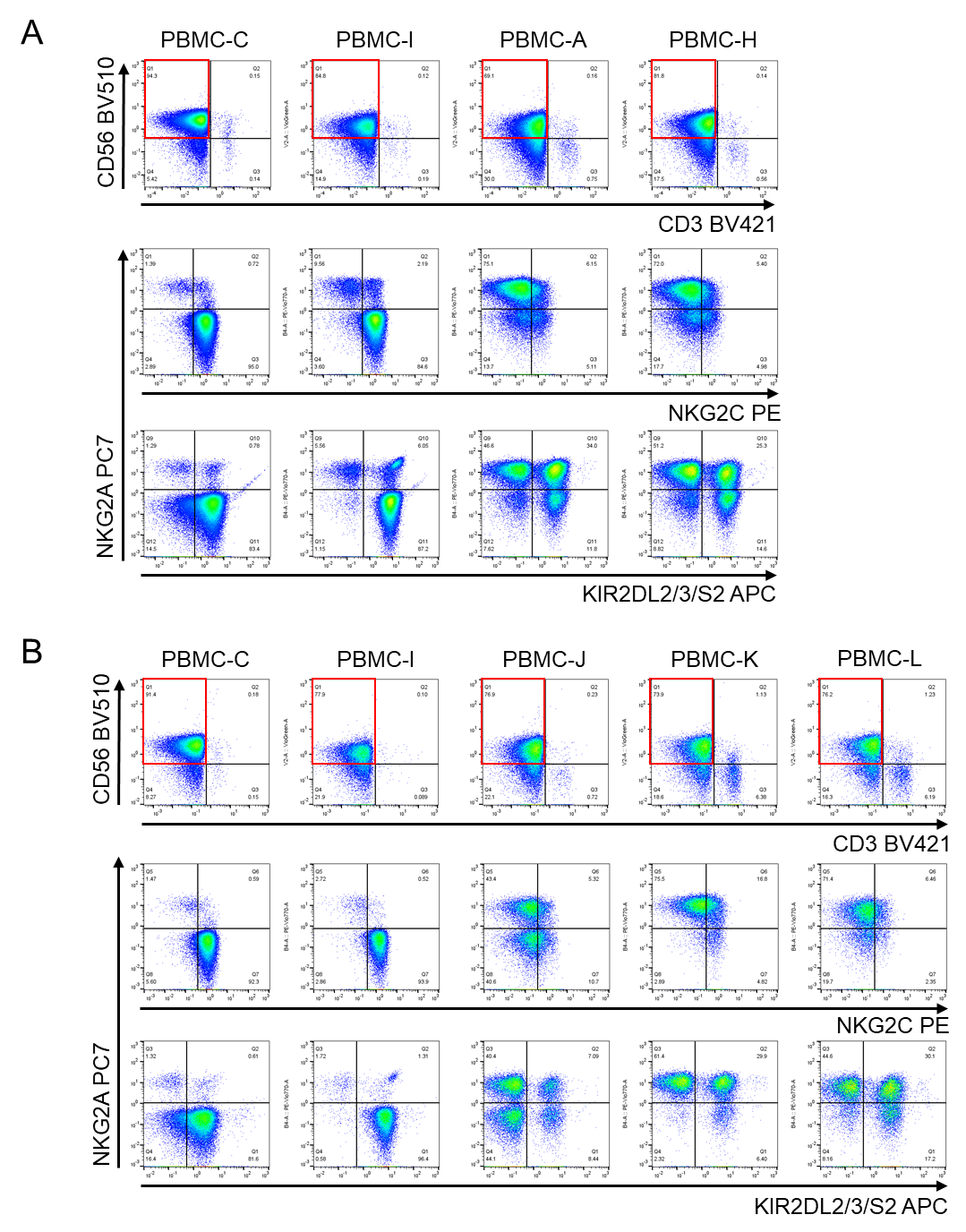


Supplementary Figure 16. Preparation of NKG2A-rich or NKG2C-rich PBMC-derived NK cells for NK cell cytotoxicity assay.

A) B) The expression of NKG2A, NKG2C and KIR2DL2/3/S2 on PBMC-derived NK cells was detected by flow cytometry. CD3-/CD56+ cells were treated as NK cells (red frame). PBMC-derived NK cells were expanded using anti-CD16 antibody. All PBMC assessed in Supplementally Figure 16 carry HLA-C1/C1.


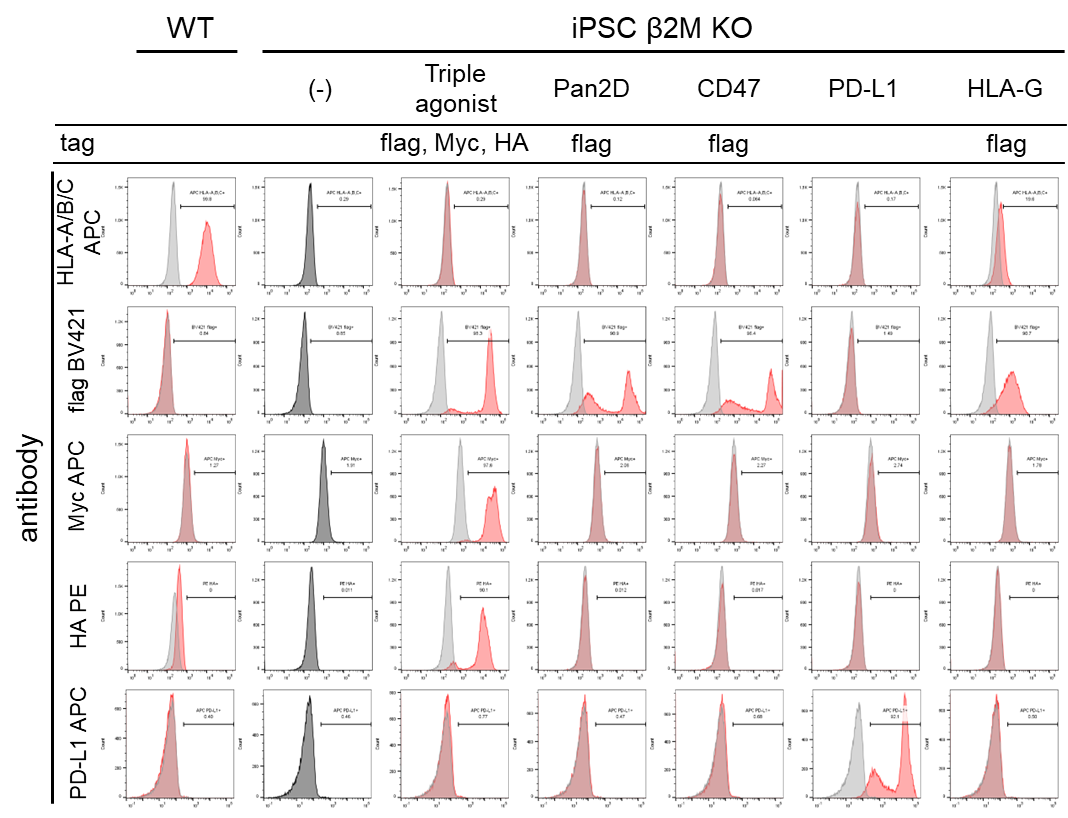


Supplementary Figure 17. Introduction of triple novel KIR agonists or ligands of other inhibitory receptors on NK cells to iPSCs.

The expression of the endogenous HLA-A/B/C, KIR agonists and other ligands on iPSCs was detected by flow cytometry and compared to B2M KO iPSCs (gray). The KIR2DL2/3 agonist 61 was tagged with Myc. The KIR2DL1 agonist 395 was tagged with HA. The KIR3DL1 agonist 1295, Pan2D scFv, CD47 and B2M with HLA-G*0101 chimera protein were tagged with flag.


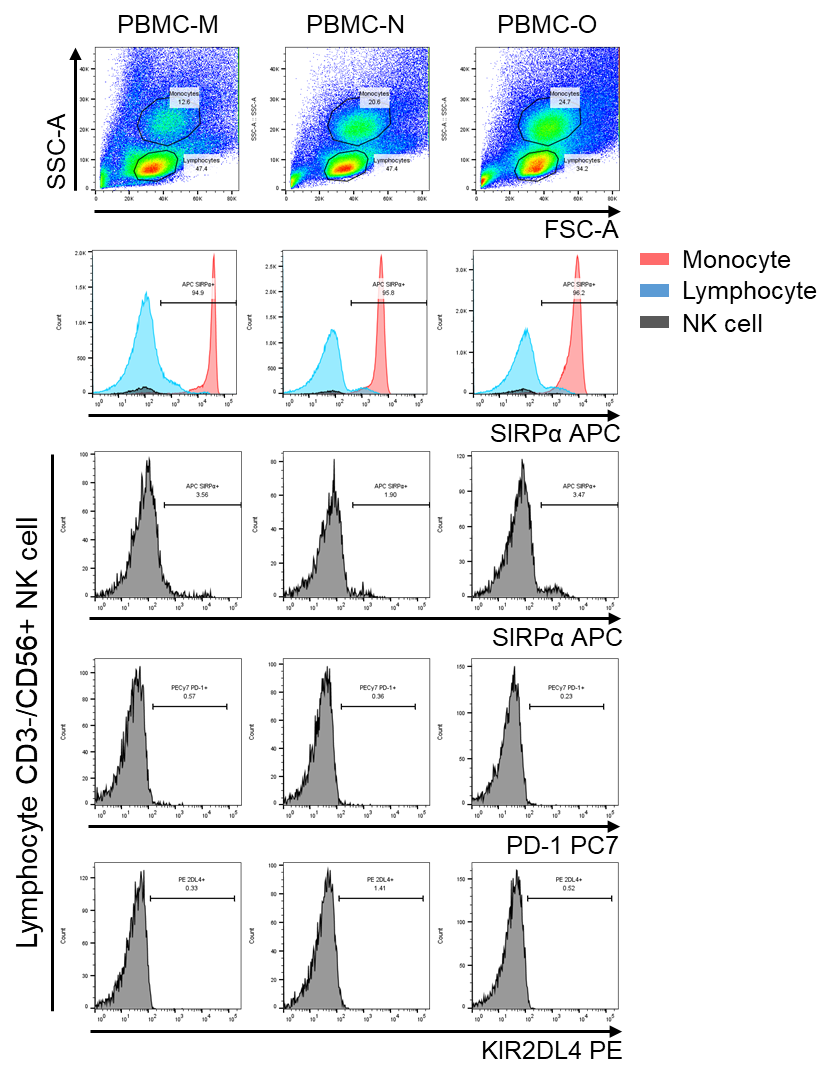


Supplementary Figure 18. SIRPα was not expressed on lymphocyte NK cells following the thawing of PBMC freeze stocks.

A) The expression of SIRPα, PD-1 and KIR2DL4 on PBMCs was detected by flow cytometry. Monocytes and lymphocytes were distinguished using SSC-FSC scattergram (top). The expression of SIRPα on monocytes (red), lymphocytes (blue) and NK cells (gray) is overlaid (middle). The expression of SIRPα, PD-1 and KIR2DL4 on PBMC-derived NK cells is shown (bottom).

Supplementary Table 1. Summary of the epitopes of KIR binders.

| **Binder** | **Epitope** |
| --- | --- |
| KIR2DL2/3 agonist 61 | Glu42, His61, Lys65-Leu70, Ser86-Pro89, Met91-Leu94, Tyr101, Leu111 |
| Lirilumab | His57, Leu59, His61, Arg62, Lys65-Iso73, Phe85, Ser86, Asp93, Pro108, Tyr109, Leu111 |
| Pan2D | Lys65-Lys67, Gln92-Asp93, Tyr126-Glu127, Arg152-Ser154, Asp156, Phe181, Phe202-Pro206 |

Supplementary Table 2. Summary of the result of homology modeling.

| **Binder** | **Combination** | **Different residue** | **Influence** |
| --- | --- | --- | --- |
| KIR2DL2/3 agonist 61 | 2DL2/2DS1 | Gly88 to Ser88 | Steric hindrance, Loss of Hydrogen bonding |
|  |  | Pro89 to Arg89 | Loss or weekness of Hydrogen bonding |
|  | 2DL2/2DS2 | Phe66 to Tyr66 | Steric hindrance, Loss of Hydrogen bonding |
|  | 2DL2/2DS4 | Asp68 to Asn68 | Steric hindrance, Loss of Hydrogen bonding |
|  |  | Asp93 to Val93 | Loss of Hydrogen bonding |
| Lirilumab | 2DL3/2DS1 | Lys67 to Asn67 | Loss of cation-pi interaction |
|  |  | His71 to Arg71 | Loss of Hydrogen bonding |
|  | 2DL3/2DS2 | Phe66 to Tyr66 | Mild steric hindrance |
|  | 2DL3/2DS4 | Lys67 to Asn67 | Loss of cation-pi interaction |
|  |  | Asp68 to Asn68 | Steric hindrance, Weakness of Hydrogen bonding |
|  |  | Asp93 to Val93 | Loss of Hydrogen bonding |
| Pan2D | 2DL2/2DS1 | Lys65 to Met65 | Loss of Hydrogen bonding via water molecule |
|  | 2DL2/2DS2 | Phe66 to Tyr66 | No influence |
|  | 2DL2/2DS4 | Gln92 to Pro92 | Loss of Hydrogen bonding via water molecule |

Supplementary Table 3. KIR subtype frequencies of each KIR family member (the % of individuals carrying KIR subtype).

| Mexico  (Allele Frequency) | 0.9 | 16.8 | 72.9 |  |  |  |  |  | 9.3 |  |  |  |  |  |  |  |  |
| --- | --- | --- | --- | --- | --- | --- | --- | --- | --- | --- | --- | --- | --- | --- | --- | --- | --- |
| India | 3.4 | 21.9 | 80.8 | 43.8 |  |  |  |  | 8.4 |  |  |  |  |  |  |  |  |
| American African | 2.0 | 13.0 | 76.0 |  | 11.0 | 1.0 | 1.0 | 1.0 | 24.0 | 1.0 | 3.0 | 7.0 | 5.0 | 2.0 | 3.0 | 1.0 |  |
| American Caucasian | 6.6 | 45.3 | 68.0 |  |  |  |  |  | 22.7 |  |  |  |  |  |  |  |  |
| Japan  (Allele Frequency) |  | 6.5 | 89.7 |  |  |  |  |  | 0.9 |  |  |  |  |  |  |  |  |
| Amino acid differences | |  |  | P16R, P124L | | | | | | P16R, P154T, D163N, H182R, K216E | | | P16R, I99V, P154T, D163N, H182R, K216E | P16R | | P16R, P124L, G179S | P16R, P124L, R221Q |
|  | | 001 | 002 | 003 | 005 | 006 | 015 | 016 | 018 | 004 | 010 | 011 | 007 | 012 | 019 | 014 | 017 |
|  | | KIR2DL1 | | | | | | | | | | | | | | | |

| Brazil Mixed | 43.2 | 23.8 |  |  |
| --- | --- | --- | --- | --- |
| Oman | 33.7 | 18.1 | 2.6 |  |
| South Africa | 30.9 | 53.5 | 4.1 |  |
| Ireland Northern | 31.2 | 18.2 |  |  |
| Japan  (Allele Frequency) | 2.2 | 3.9 |  |  |
| Amino acid differences | |  | I200T | R16P, E35Q, R41T, G167D, I200T, K216E |
|  | | 001 | 003 | 004 |
|  | | KIR2DL2 | | |

| Brazil Mixed | 80.4 |  |  | 19.5 |  | 1.0 |  |
| --- | --- | --- | --- | --- | --- | --- | --- |
| Oman | 67.0 |  |  | 24.2 |  |  | 13.1 |
| South Africa | 42.0 |  | 4.0 | 6.0 |  |  | 12.0 |
| Ireland Northern | 63.6 | 4.5 |  | 46.8 |  |  | 6.5 |
| Japan  (Allele Frequency) | 87.1 |  |  | 3.9 | 2.2 |  |  |
| Amino acid differences |  | | | P208L | | L11R, Q35E, H50R, R221I | L11R, Q35E, H50R |
|  | 001 | 003 | 006 | 002 | 007 | 004 | 005 |
|  | KIR2DL3 | | | | | | |

|  |  |  |  | Intracellular retention subtype |  |  |  |  |  |  |  |  |  |  |  |
| --- | --- | --- | --- | --- | --- | --- | --- | --- | --- | --- | --- | --- | --- | --- | --- |
| Brazil Mixed | 28.9 |  | 20.0 | 23.0 | 21.5 | 7.4 | 28.2 |  |  | 3.0 | 2.2 | 3.7 | 0.7 | 10.4 |  |
| Oman | 40.4 |  | 13.1 | 17.2 | 12.1 | 39.4 | 8.1 |  |  | 14.1 | 1.0 |  |  |  |  |
| South Africa | 17.0 | 1.0 | 3.0 | 17.0 | 11.0 | 8.0 | 52.0 | 2.0 | 10.0 | 3.0 |  | 10.0 | 2.0 | 5.0 | 6.0 |
| Ireland Northern | 33.8 | 1.4 | 31.1 | 19.0 | 25.7 | 4.1 | 10.8 |  |  | 17.6 | 1.4 |  | 2.7 | 2.7 |  |
| Japan  (Allele Frequency) | 6.0 |  |  |  | 12.8 | 12.8 | 45.7 |  |  |  |  |  |  | 6.0 |  |
| Amino acid differences |  | | D2V, I47V, I54L  G238R" | R31H, R44G, S66L, P182S, W283L | P182S, W283L | D2V, I47V, I54L | | | | D2V, R31H, I47V, I54L | I47V, S58G, V92M | I47V, I54L | Y30C, R31H, R44G, S66L, P182S, W283L | D2V, I47V, I54L, R145S | I47V, I54L, R277C |
|  | 001 | 016 | 002 | 004 | 005 | 007 | 015 | 025 | 031 | 008 | 009 | 017 | 019 | 020 | 022 |
|  | KIR3DL1 | | | | | | | | | | | | | | |

| Brazil Caucasian | 36.7 |  | 1.1 |  |
| --- | --- | --- | --- | --- |
| Oman | 32.3 |  |  |  |
| American African | 22.0 | 1.0 | 2.0 | 3.0 |
| American Caucasian | 39.0 |  |  |  |
| Japan  (Allele Frequency) | 33.3 |  |  |  |
| Amino acid differences | R70K | | R70K, L90V, E216K | R70K, L90V, N123S |
|  | 002 | 006 | 004 | 008 |
|  | KIR2DS1 | | | |

| American African | 41.0 |  |  | 3.0 |
| --- | --- | --- | --- | --- |
| American Caucasian | 44.2 | 7.8 | 1.3 |  |
| Japan  (Allele Frequency) | 11.4 |  |  |  |
| Amino acid differences |  |  |  |  |
|  | 001 | 002 | 005 | 006 |
|  | KIR2DS2 | | | |

|  | Full length | | Intracellular domaim deleted inactive subtype | | | | | | |
| --- | --- | --- | --- | --- | --- | --- | --- | --- | --- |
| Brazil Caucasian | 60.0 |  | 37.8 | 31.1 |  | 8.9 |  |  |  |
| Oman | 39.4 |  | 63.6 | 19.2 |  | 6.1 |  |  |  |
| American African | 68.0 | 2.0 | 24.0 | 20.0 | 5.0 | 8.0 | 10.0 | 1.0 | 4.0 |
| American Caucasian | 42.9 |  | 48.1 | 20.8 |  | 3.9 | 24.7 |  |  |
| Japan  (Allele Frequency) | 50.4 |  | 6.0 |  |  | 12.9 | 12.9 |  |  |
| Amino acid differences |  | |  | | | G81S, T125S | P84T | A74E | V4A |
|  | 001 | 011 | 003 | 006 | 009 | 004 | 007 | 012 | 013 |
|  | KIR2DS4 | | | | | | | | |

| Population | AFND population name |
| --- | --- |
| Japan | Japan KIR pop 3 (n=132) |
| Caucasian | USA Caucasian NMDP KIR (n=75) |
|  | Ireland Northern KIR pop2 (n=154) |
| African | USA African American KIR pop 2 (n=100) |
|  | South Africa Xhosa KIR (n=50) |
| Asia | India North Hindu KIR (n=72) |
|  | Oman KIR (n=99) |
| Latin America | Mexico Mexico City Mestizo KIR (n=86) |
|  | Brazil Rio de Janeiro Mixed KIR (n=166) |
|  | Brazil Belo Horizonte Caucasian KIR (n=90) |

The % of individuals who have the allele are indicated. Some samples indicate the % of allele frequencies instead of the % of individuals.

KIR subtypes are classified according to the amino acid sequence of extracellular domain.

Amino acid differences exist in extracellular domain of each KIR subtype are indicated.

Red letters show KIR subtypes tested in this study.

Names of cohort are given exactly as written in the Allele frequency net database (AFND).

Link to AFND: http://www.allelefrequencies.net

Supplementary Table 4. Information of protein sequences.

| **Protein** | **Sequence ID** | **Source of ID** | **Length** |
| --- | --- | --- | --- |
| EGFP | AAB02572.1 | Genbank | full length |
| mCherry | AAV52164.1 | Genbank | full length |
| Firefry luciferase | AHC94771.1 | Genbank | full length |
| Truncated CD8 | NP_001759.3 | NCBI | 1-208 |
| CD8 hinge | NP_001759.3 | NCBI | 138-208 |
| CD8 long hinge | NP_001759.3 | NCBI | 117-208 |
| Truncated CD19 | NP_001171569.1 | NCBI | 1-333 |
| Truncated NGFR | NP_002498.1 | NCBI | 29-277 |
| B2M | NP_004039.1 | NCBI | full length |
| HLA-C*0102 | HLA00401 | IPD accession No. | 25-366 |
| HLA-C*0202 | HLA00404 | IPD accession No. | 25-366 |
| HLA-C*0302 | HLA00410 | IPD accession No. | 25-366 |
| HLA-C*0303 | HLA00411 | IPD accession No. | 25-366 |
| HLA-C*0304 | HLA00413 | IPD accession No. | 25-366 |
| HLA-C*0401 | HLA00420 | IPD accession No. | 25-366 |
| HLA-C*0602 | HLA00430 | IPD accession No. | 25-366 |
| HLA-C*0701 | HLA00433 | IPD accession No. | 25-366 |
| HLA-C*0702 | HLA00434 | IPD accession No. | 25-366 |
| HLA-C*0801 | HLA00445 | IPD accession No. | 25-366 |
| HLA-C*0802 | HLA00446 | IPD accession No. | 25-366 |
| HLA-C*1202 | HLA00453 | IPD accession No. | 25-366 |
| HLA-C*1203 | HLA00455 | IPD accession No. | 25-366 |
| HLA-C*1402 | HLA00462 | IPD accession No. | 25-366 |
| HLA-C*1502 | HLA00467 | IPD accession No. | 25-366 |
| HLA-C*1601 | HLA00475 | IPD accession No. | 25-366 |
| HLA-C*1701 | HLA04311 | IPD accession No. | 25-372 |
| HLA-E*0101 | HLA00934 | IPD accession No. | 22-358 |
| HLA-E*0103 | HLA00936 | IPD accession No. | 22-358 |
| HLA-G*0101 | HLA00939 | IPD accession No. | 25-338 |
| KIR2DL1*001 | KIR00001 | IPD accession No. | full length |
| KIR2DL1*002 | KIR00002 | IPD accession No. | full length |
| KIR2DL1*004 | KIR00006 | IPD accession No. | full length |
| KIR2DL2*001 | KIR00010 | IPD accession No. | full length |
| KIR2DL2*003 | KIR00012 | IPD accession No. | full length |
| KIR2DL3*001 | KIR00014 | IPD accession No. | full length |
| KIR2DL3*002 | KIR00015 | IPD accession No. | full length |
| KIR2DL3*005 | KIR00018 | IPD accession No. | full length |
| KIR3DL1*001 | KIR00054 | IPD accession No. | full length |
| KIR3DL1*002 | KIR00056 | IPD accession No. | full length |
| KIR3DL1*005 | KIR00060 | IPD accession No. | full length |
| KIR3DL1*008 | KIR00063 | IPD accession No. | full length |
| KIR3DL1*015 | KIR00057 | IPD accession No. | full length |
| KIR2DS1*002 | KIR00034 | IPD accession No. | full length |
| KIR2DS2*001 | KIR00037 | IPD accession No. | full length |
| KIR2DS4*001 | KIR00045 | IPD accession No. | full length |
| CD47 | NP_001768.1 | NCBI | full length |
| PD-L1 | NP_054862.1 | NCBI | full length |

Supplementary Table 5. Nucleotide sequences of sgRNAs and primers.

| **Target of sgRNA** | **Sequence** |
| --- | --- |
| B2M | gctgtctataaatagtcctc |
| ACTB | tgagcagccttagagggtgg |

| **Primer** | **Sequence** |
| --- | --- |
| B2M left arm Fwd  (with additional 25bp for gibson assembly) | CTTGGGCAGAGGTGGAGGTGTAGAACCTGATATAGCTTGACACCAAGTTA |
| B2M left arm Rev  (with additional 25bp for gibson assembly) | CGACGGTCTCCGATCGATGTCTCGGTTACATGTCTCGATCTATGAAAAAGACA |
| B2M right arm Fwd  (with additional 25bp for gibson assembly) | GAAAGTGTCTCCGATCAACGCGGGGGACTATTTATAGACAGCTCTAACATGATAA |
| B2M right arm Rev  (with additional 25bp for gibson assembly) | CTGAACTTAACCATCCTGCTTCCACTTTGGTCAATGATGGACTGTATATATGA |
| ACTB left arm Fwd  (with additional 25bp for gibson assembly) | CACGTTAAGGGATTTTGGTCATGAGCCCGCTACCTCTTCTGGTGG |
| ACTB left arm Rev  (with additional 25bp for gibson assembly) | TCGTCCACCGCAAATGCTTCGGAAGTGGAGAGGGCAGAGGGAGCC |
| ACTB right arm Fwd  (with additional 25bp for gibson assembly) | ACGGTCTCCGATCGATGTCTCGGTGGGGGAGGCTCAGGGGTCA |
| ACTB right arm Rev  (with additional 25bp for gibson assembly) | TGGAGGAGCTGTCAGTCTGGAAGACGTCAGGTGGCACTTTTCGGGGA |

Supplementary Table 6. Allele frequencies of HLA-C (% of alleles).

| C*18:01 | C2 |  |  |  |  |  |  | 3.25 |  |  |  |
| --- | --- | --- | --- | --- | --- | --- | --- | --- | --- | --- | --- |
| C*17:01 | C2 |  |  | 1.21 |  |  |  | 7.15 | 1.44 |  | 4.62 |
| C*16:02 | C1 |  |  |  |  |  |  |  |  | 2.57 | 2.76 |
| C*16:01 | C1 |  | 2.2 | 8.07 |  | 6.88 | 3.38 | 9.14 | 4.73 |  | 1.33 |
| C*15:02 | C2 | 3.08 | 2.27 | 2.83 | 3.12 |  | 2.23 |  | 3.55 | 10.38 | 10.73 |
| C*14:03 | C1 | 6.69 | 0.02 |  | 0.67 |  |  |  |  |  |  |
| C*14:02 | C1 | 6.87 | 1.34 | 1.86 | 4.18 |  | 1.27 | 1.55 | 0.95 | 5.77 | 2.49 |
| C*12:03 | C1 |  | 5.73 | 6.86 | 1.39 | 7.45 | 4.86 | 1.83 | 3.93 | 3.71 | 4.28 |
| C*12:02 | C1 | 11.18 | 0.85 | 1.31 | 2.45 |  | 0.87 | 0.28 | 1.12 | 8.29 | 2.42 |
| C*08:02 | C1 |  | 2.31 | 5.55 | 0.23 | 5.16 | 3.85 | 3.68 | 5.12 |  | 2.32 |
| C*08:01 | C1 | 7.36 | 0.03 | 0.06 | 7.85 |  |  |  | 3.62 | 1.89 |  |
| C*07:04 | C1 |  | 2.05 |  |  |  |  |  |  | 2.38 |  |
| C*07:02 | C1 | 12.71 | 13.93 | 9.07 | 16.91 | 6.45 | 14.13 | 6.83 | 13.66 | 12.66 | 12.23 |
| C*07:01 | C1 | 0.07 | 14.73 | 13.01 | 0.91 | 10.89 | 16 | 13.06 | 9.17 | 8.41 | 9.66 |
| C*06:02 | C2 | 0.81 | 10.08 | 7.37 | 6.98 | 8.31 | 9.32 | 8.78 | 6.03 | 16 | 16.67 |
| C*05:01 | C2 |  | 7.02 | 10.6 | 0.51 | 7.7 | 9.39 | 2.46 | 5.75 | 0.38 | 1.32 |
| C*04:01 | C2 | 4.41 | 11.47 | 12.77 | 5.24 | 13.32 | 10.59 | 20.72 | 17.1 | 10.83 | 12.15 |
| C*03:04 | C1 | 12.39 | 8.16 | 3 | 11.75 | 4.16 | 7.49 | 4.86 | 7.26 | 2 | 1.42 |
| C*03:03 | C1 | 13.05 | 5.71 | 3.48 | 6.85 | 5.16 | 5.34 | 0.91 | 3.66 | 1.55 | 0.61 |
| C*03:02 | C1 |  |  |  | 6.11 |  |  | 1.77 | 0.37 | 3.95 | 2.82 |
| C*02:02 | C2 |  | 5.63 | 5.09 | 0.29 | 4.44 | 4.35 | 8.47 | 3.36 |  | 1.72 |
| C*01:02 | C1 | 17.6 | 3.51 | 3.78 | 20.23 | 1.44 | 3.41 | 0.48 | 5.07 | 3.57 | 1.5 |
| HLA allele | Type | Japan | German | Spain | China | Brazil Caucasian | US Caucasian | US African | USA Mexican | India | Saudi Arabia |

| Population | AFND population name |
| --- | --- |
| Japan | Japan pop 16 (n=18604) |
| German | Germany DKMS - German donors (n=3456066) |
| Spain | Spain (Catalunya, Navarra, Extremadura, Aaragón, Cantabria, (n=4335) |
| China | China Hubei Han (n=3732) |
| Brazil Caucasian | Brazil Rio de Janeiro Caucasian (n=521) |
| US Caucasian | USA NMDP European Caucasian (n=1242890) |
| US African | USA NMDP African (n=28557) |
| USA Mexican | USA NMDP Mexican or Chicano (n=261235) |
| India | India Tamil Nadu (n=2492) |
| Saudi Arabia | Saudi Arabia pop 6 (G) (n=28927) |

The % of allele frequencies rather than the % of individuals with the allele are indicated.

HLA-C alleles not tested in this study are highlighted in gray.

Names of cohort are given exactly as written in the Allele frequency net database (AFND).

Link to AFND: http://www.allelefrequencies.net

Supplementary Table 7. List of all antibodies.

| **Antibody** | **Source** | **Catalog#** |
| --- | --- | --- |
| anti-DYKDDDDK(FLAG) tag-BV421 | Biolegend | 637322 |
| anti-DYKDDDDK(FLAG) tag-PE | Biolegend | 637310 |
| anti-DYKDDDDK(FLAG) tag-APC | Biolegend | 637308 |
| anti-Myc tag-FITC | ABCAM | ab202008 |
| anti-Myc tag-Alexa Fluor 647 | MEDICAL & BIOLOGICAL LABORATORIES | M047-A64 |
| anti-HA tag-PE | Miltenyi Biotec | 130-120-786 |
| anti-HA tag-APC | Biolegend | 901523 |
| anti-NGFR-APC | Biolegend | 345108 |
| anti-NGFR-APC/Cy7 | Biolegend | 345126 |
| anti-CD3-BV421 | Biolegend | 300434 |
| anti-CD3-APC | Biolegend | 317318 |
| anti-CD19-PE | Biolegend | 363004 |
| anti-CD19-APC | Biolegend | 302212 |
| anti-CD56-BV510 | Biolegend | 362534 |
| anti-CD56-PE | Biolegend | 318306 |
| anti-KIR2DL1/2DS5-PE | R&D systems | FAB1844P |
| anti-KIR2DL1-PE | Miltenyi Biotec | 130-120-586 |
| anti-KIR2DL1/2DS1/2DS3/2DS5-PC5.5 | Biolegend | 339514 |
| anti-KIR2DL1/2DS1-APC | Miltenyi Biotec | 130-092-685 |
| anti-KIR2DL2/KIR2DL3/2DS2-PE | Miltenyi Biotec | 130-116-832 |
| anti-KIR2DL2/KIR2DL3/2DS2-APC | Miltenyi Biotec | 130-092-617 |
| anti-KIR2DL2/KIR2DL3/2DS2-APC/Cy7 | Miltenyi Biotec | 130-116-836 |
| anti-KIR2DL4-PE | R&D systems | FAB2238P |
| anti-KIR2DS1-Alexa Fluor 647 | R&D systems | FAB8887G |
| anti-KIR2DS4-APC | R&D systems | FAB1847A |
| anti-KIR2DS4-APC | Miltenyi Biotec | 130-114-773 |
| anti-KIR3DL1-BV421 | Biolegend | 312714 |
| anti-KIR3DL1-FITC | Biolegend | 312706 |
| anti-KIR3DL1/KIR3DS1-APC | Beckman Coulter | A60795 |
| anti-KIR3DL1/KIR3DL2/3DS1-APC/Cy7 | Miltenyi Biotec | 130-116-181 |
| anti-NKG2A-PC7 | Beckman Coulter | B10246 |
| anti-NKG2C-PE | Biolegend | 130-119-776 |
| anti-HLA-A,B,C-APC | Biolegend | 311410 |
| anti-HLA-E | Biolegend | 342606 |
| anti-β2-microglobulin Antibody-APC | Biolegend | 316312 |
| anti-PD-L1-APC | Biolegend | 329708 |
| anti-PD-1-PC7 | Biolegend | 329918 |
| anti-SIRPα-APC | Biolegend | 323810 |

Supplementary Table 8. Recombinant protein sequences used for phage display and X-ray crystallization.

| **Phage display** | |
| --- | --- |
| **Protein** | **sequence** |
| hKIR2DL1*002(22-245)-Fc-Avidin | MYRMQLLSCIALSLALVTNSHEGVHRKPSLLAHPGRLVKSEETVILQCWSDVMFEHFLLHREGMFNDTLRLIGEHHDGVSKANFSISRMTQDLAGTYRCYGSVTHSPYQVSAPSDPLDIVIIGLYEKPSLSAQLGPTVLAGENVTLSCSSRSSYDMYHLSREGEAHERRLPAGPKVNGTFQADFPLGPATHGGTYRCFGSFHDSPYEWSKSSDPLLVSVTGNPSNSWPSPTEPSSKTGNPRHLHGGGSDKTHTCPPCPAPELLGGPSVFLFPPKPKDTLMISRTPEVTCVVVDVSHEDPEVKFNWYVDGVEVHNAKTKPREEQYNSTYRVVSVLTVLHQDWLNGKEYKCKVSNKALPAPIEKTISKAKGQPREPQVYTLPPSREEMTKNQVSLTCLVKGFYPSDIAVEWESNGQPENNYKTTPPVLDSDGSFFLYSKLTVDKSRWQQGNVFSCSVMHEALHNHYTQKSLSLSPGKGAAGLNDIFEAQKIEWHE |
| hKIR2DL2*001(22-245)-Fc-Avidin | MYRMQLLSCIALSLALVTNSHEGVHRKPSLLAHPGRLVKSEETVILQCWSDVRFEHFLLHREGKFKDTLHLIGEHHDGVSKANFSIGPMMQDLAGTYRCYGSVTHSPYQLSAPSDPLDIVITGLYEKPSLSAQPGPTVLAGESVTLSCSSRSSYDMYHLSREGEAHECRFSAGPKVNGTFQADFPLGPATHGGTYRCFGSFRDSPYEWSNSSDPLLVSVIGNPSNSWPSPTEPSSKTGNPRHLHGGGSDKTHTCPPCPAPELLGGPSVFLFPPKPKDTLMISRTPEVTCVVVDVSHEDPEVKFNWYVDGVEVHNAKTKPREEQYNSTYRVVSVLTVLHQDWLNGKEYKCKVSNKALPAPIEKTISKAKGQPREPQVYTLPPSREEMTKNQVSLTCLVKGFYPSDIAVEWESNGQPENNYKTTPPVLDSDGSFFLYSKLTVDKSRWQQGNVFSCSVMHEALHNHYTQKSLSLSPGKGAAGLNDIFEAQKIEWHE |
| hKIR2DL3*001(22-245)-Fc-Avidin | MYRMQLLSCIALSLALVTNSHEGVHRKPSLLAHPGPLVKSEETVILQCWSDVRFQHFLLHREGKFKDTLHLIGEHHDGVSKANFSIGPMMQDLAGTYRCYGSVTHSPYQLSAPSDPLDIVITGLYEKPSLSAQPGPTVLAGESVTLSCSSRSSYDMYHLSREGEAHERRFSAGPKVNGTFQADFPLGPATHGGTYRCFGSFRDSPYEWSNSSDPLLVSVTGNPSNSWPSPTEPSSETGNPRHLHGGGSDKTHTCPPCPAPELLGGPSVFLFPPKPKDTLMISRTPEVTCVVVDVSHEDPEVKFNWYVDGVEVHNAKTKPREEQYNSTYRVVSVLTVLHQDWLNGKEYKCKVSNKALPAPIEKTISKAKGQPREPQVYTLPPSREEMTKNQVSLTCLVKGFYPSDIAVEWESNGQPENNYKTTPPVLDSDGSFFLYSKLTVDKSRWQQGNVFSCSVMHEALHNHYTQKSLSLSPGKGAAGLNDIFEAQKIEWHE |
| hKIR3DL1*015(22-340)-Fc-Avidin | MYRMQLLSCIALSLALVTNSHVGGQDKPFLSAWPSAVVPRGGHVTLRCHYRHRFNNFMLYKEDRIHVPIFHGRLFQESFNMSPVTTAHAGNYTCRGSHPHSPTGWSAPSNPVVIMVTGNHRKPSLLAHPGPLVKSGERVILQCWSDIMFEHFFLHKEGISKDPSRLVGQIHDGVSKANFSIGPMMLALAGTYRCYGSVTHTPYQLSAPSDPLDIVVTGPYEKPSLSAQPGPKVQAGESVTLSCSSRSSYDMYHLSREGGAHERRLPAVRKVNRTFQADFPLGPATHGGTYRCFGSFRHSPYEWSDPSDPLLVSVTGNPSSSWPSPTEPSSKSGNPRHLHGGGSDKTHTCPPCPAPELLGGPSVFLFPPKPKDTLMISRTPEVTCVVVDVSHEDPEVKFNWYVDGVEVHNAKTKPREEQYNSTYRVVSVLTVLHQDWLNGKEYKCKVSNKALPAPIEKTISKAKGQPREPQVYTLPPSREEMTKNQVSLTCLVKGFYPSDIAVEWESNGQPENNYKTTPPVLDSDGSFFLYSKLTVDKSRWQQGNVFSCSVMHEALHNHYTQKSLSLSPGKGAAGLNDIFEAQKIEWHE |
| hKIR2DS1*002(22-245)-Fc-Avidin | MYRMQLLSCIALSLALVTNSHEGVHRKPSLLAHPGRLVKSEETVILQCWSDVMFEHFLLHREGMFNDTLRLIGEHHDGVSKANFSISRMKQDLAGTYRCYGSVTHSPYQLSAPSDPLDIVIIGLYEKPSLSAQPGPTVLAGENVTLSCSSRSSYDMYHLSREGEAHERRLPAGTKVNGTFQANFPLGPATHGGTYRCFGSFRDSPYEWSKSSDPLLVSVTGNPSNSWPSPTEPSSETGNPRHLHGGGSDKTHTCPPCPAPELLGGPSVFLFPPKPKDTLMISRTPEVTCVVVDVSHEDPEVKFNWYVDGVEVHNAKTKPREEQYNSTYRVVSVLTVLHQDWLNGKEYKCKVSNKALPAPIEKTISKAKGQPREPQVYTLPPSREEMTKNQVSLTCLVKGFYPSDIAVEWESNGQPENNYKTTPPVLDSDGSFFLYSKLTVDKSRWQQGNVFSCSVMHEALHNHYTQKSLSLSPGKGAAGLNDIFEAQKIEWHE |
| hKIR2DS2*001(22-245)-Fc-Avidin | MYRMQLLSCIALSLALVTNSHEGVHRKPSLLAHPGPLVKSEETVILQCWSDVRFEHFLLHREGKYKDTLHLIGEHHDGVSKANFSIGPMMQDLAGTYRCYGSVTHSPYQLSAPSDPLDIVITGLYEKPSLSAQPGPTVLAGESVTLSCSSRSSYDMYHLSREGEAHERRFSAGPKVNGTFQADFPLGPATHGGTYRCFGSFRDSPYEWSNSSDPLLVSVTGNPSNSWPSPTEPSSKTGNPRHLHGGGSDKTHTCPPCPAPELLGGPSVFLFPPKPKDTLMISRTPEVTCVVVDVSHEDPEVKFNWYVDGVEVHNAKTKPREEQYNSTYRVVSVLTVLHQDWLNGKEYKCKVSNKALPAPIEKTISKAKGQPREPQVYTLPPSREEMTKNQVSLTCLVKGFYPSDIAVEWESNGQPENNYKTTPPVLDSDGSFFLYSKLTVDKSRWQQGNVFSCSVMHEALHNHYTQKSLSLSPGKGAAGLNDIFEAQKIEWHE |
| hKIR2DS4*001(22-245)-Fc-Avidin | MYRMQLLSCIALSLALVTNSQEGVHRKPSFLALPGHLVKSEETVILQCWSDVMFEHFLLHREGKFNNTLHLIGEHHDGVSKANFSIGPMMPVLAGTYRCYGSVPHSPYQLSAPSDPLDMVIIGLYEKPSLSAQPGPTVQAGENVTLSCSSRSSYDMYHLSREGEAHERRLPAVRSINGTFQADFPLGPATHGGTYRCFGSFRDAPYEWSNSSDPLLVSVTGNPSNSWPSPTEPSSKTGNPRHLHGGGSDKTHTCPPCPAPELLGGPSVFLFPPKPKDTLMISRTPEVTCVVVDVSHEDPEVKFNWYVDGVEVHNAKTKPREEQYNSTYRVVSVLTVLHQDWLNGKEYKCKVSNKALPAPIEKTISKAKGQPREPQVYTLPPSREEMTKNQVSLTCLVKGFYPSDIAVEWESNGQPENNYKTTPPVLDSDGSFFLYSKLTVDKSRWQQGNVFSCSVMHEALHNHYTQKSLSLSPGKGAAGLNDIFEAQKIEWHE |
| Fc-His-Avidin | MKHLWFFLLLVAAPRWVLSDKTHTCPPCPAPELLGGPSVFLFPPKPKDTLMISRTPEVTCVVVDVSHEDPEVKFNWYVDGVEVHNAKTKPREEQYNSTYRVVSVLTVLHQDWLNGKEYKCKVSNKALPAPIEKTISKAKGQPREPQVYTLPPSREEMTKNQVSLTCLVKGFYPSDIAVEWESNGQPENNYKTTPPVLDSDGSFFLYSKLTVDKSRWQQGNVFSCSVMHEALHNHYTQKSLSLSPGKGGGSHHHHHHGSGLNDIFEAQKIEWHE |

| **X-ray crystalization** | |
| --- | --- |
| **Protein** | **sequence** |
| hKIR2DL2*001(22-245)-His | MYRMQLLSCIALSLALVTNSHEGVHRKPSLLAHPGRLVKSEETVILQCWSDVRFEHFLLHREGKFKDTLHLIGEHHDGVSKANFSIGPMMQDLAGTYRCYGSVTHSPYQLSAPSDPLDIVITGLYEKPSLSAQPGPTVLAGESVTLSCSSRSSYDMYHLSREGEAHECRFSAGPKVNGTFQADFPLGPATHGGTYRCFGSFRDSPYEWSNSSDPLLVSVIGNPSHHHHHH |
| Pan2D Fab heavy | MKHLWFFLLLVAAPRWVLSEVQLQQSGTVLARPGASVKMSCKASGYTFTSYWMHWMKQRPGQGLEWIGTIYPGNSDTNYNQKFKGKAKLTAVTSTNTAYMELSSLTNEDSAVYYCSRPTTATRSSAMDYWGQGTSVTVSSASTKGPSVFPLAPSSKSTSGGTAALGCLVKDYFPEPVTVSWNSGALTSGVHTFPAVLQSSGLYSLSSVVTVPSSSLGTQTYICNVNHKPSNTKVDKKVEPKSCDKTH |
| Pan2D Fab light | MVLQTQVFISLLLWISGAYGQIVLTQSPASMSASLGERVTMTCTASSSVSSSYLYWYQQKPGSSPKLWIYSTSNLASGVPARFSGSGSGTSYSLTISSMEAEDAATYYCHQYHRSPPTFGGGTKLEIKRTVAAPSVFIFPPSDEQLKSGTASVVCLLNNFYPREAKVQWKVDNALQSGNSQESVTEQDSKDSTYSLSSTLTLSKADYEKHKVYACEVTHQGLSSPVTKSFNRGEC |

Supplementary Table 9. Information of recombinant proteins obtained commercially.

| **Protein** | **source** | **catalog#** |
| --- | --- | --- |
| KIR2DL1 | R&D systems | 1844-KR-050 |
| KIR2DL3 | R&D systems | 2014-KIR-050 |
| KIR2DL4 | R&D systems | 2238-KIR-050 |
| KIR2DL5 | R&D systems | 6634-KIR-050 |
| KIR3DL1 | R&D systems | 1225-KIR-050 |
| KIR3DL3 | R&D systems | 10104-KIR-050 |
| KIR3DS1 | R&D systems | 4136-KIR-050 |
| NKG2A | R&D systems | 10511-NK-050 |
| NKG2C | R&D systems | 138-NK-050 |
| NKG2D | R&D systems | 1299-NK-050 |
| NKG2E | R&D systems | 9759-NK-050 |
| NKp30 | R&D systems | 1849-NK-025 |
| NKp44 | R&D systems | 2249-NK-050 |
| NKp46 | R&D systems | 1850-NK-025 |
| CD16a | R&D systems | 4325-FC-050 |
| CD16b | R&D systems | 1597-FC-050 |
| CD32a | R&D systems | 1330-CD-050 |
| CD32b/c | R&D systems | 1875-CD-050 |
| CD64 | R&D systems | 1257-FC-050 |
| LILRB1 | R&D systems | 2017-T2-050 |
| LILRB2 | R&D systems | 8429-T4-050 |
| TIGIT | R&D systems | 9464-TG-050 |
| CD161 | R&D systems | 7448-CD-050 |
| NKG2A-CD94 | ACROBiosystems | NC4-H82E4-25ug |
| NKG2C-CD94 | ACROBiosystems | NC4-H82E3-25ug |

Supplementary Table 10. Information of recombinant proteins used for phage display.

| **Protein** | **source** | **catalog#** |
| --- | --- | --- |
| hKIR2DL1*002(22-245)-Fc-Avidin, biotinylated | Same as supplementary table 8 |  |
| hKIR2DL2*001(22-245)-Fc-Avidin, biotinylated | Same as supplementary table 8 |  |
| hKIR2DL3*001(22-245)-Fc-Avidin, biotinylated | Same as supplementary table 8 |  |
| hKIR2DS1*002(22-245)-Fc-Avidin | Same as supplementary table 8 |  |
| hKIR2DS2*001(22-245)-Fc-Avidin | Same as supplementary table 8 |  |
| hKIR2DS4*001(22-245)-Fc-Avidin | Same as supplementary table 8 |  |
| hKIR3DL1*015(22-340)-Fc-Avidin, biotinylated | Same as supplementary table 8 |  |
| hKIR3DL1*015(22-340)-Fc-Avidin | Same as supplementary table 8 |  |
| KIR3DS1 | R&D systems | 4136-KIR-050 |
| Fc-His-Avidin | Same as supplementary table 8 |  |
| Fc-His-Avidin-Biotinylated | Same as supplementary table 8 |  |

Supplementary Table 11. X-ray crystal structure analysis statistic summary.

|  | KIR2DL2 / KIR2DL2/3 agonist 61 Fab | KIR2DL2 / KIR2DL2/3 agonist 61 scFv | KIR2DL2 / Pan2D-Fab |
| --- | --- | --- | --- |
| PDB Code | 9LRF | 9LRH | 9LRA |
| **Crystallographic data** | | | |
| X-ray source | PF BL-17A | SLS PXI(X06SA) | PF BL-1A |
| Wavelength | 0.9800 | 1.0000 | 1.0450 |
| Resolution range | 38.08 - 2.5 (2.589 - 2.5)* | 44.38 - 3.4 (3.66 - 3.4) | 49.2 - 2.6 (2.693 - 2.6) |
| Space group | C 1 2 1 | I 41 2 2 | P 21 21 21 |
| Unit cell parameters | | | |
| a, b, c (Å) | 170.109, 79.244, 64.364 | 98.855, 98.855, 403.076 | 79.253, 81.515, 123.4 |
| α, β, γ (°) | 90, 92.388, 90 | 90, 90, 90 | 90, 90, 90 |
| Total reflections | 99814 (10563) | 193205 (39036) | 345589 (35823) |
| Unique reflections | 29363 (2942) | 14309 (2771) | 25263 (2496) |
| Multiplicity | 3.4 (3.6) | 13.5 (14.1) | 13.7 (14.4) |
| Completeness (%) | 98.62 (99.36) | 99.68 (99.24) | 99.95 (100.00) |
| Mean I/sigma(I) | 8.85 (0.92) | 9.23 (0.71) | 10.70 (1.42) |
| Wilson B-factor | 69.96 | 145.92 | 50.42 |
| Rmerge | 0.06794 (1.111) | 0.2038 (2.53) | 0.2168 (1.945) |
| Rmeas | 0.08071 (1.303) | 0.2118 (2.624) | 0.2252 (2.016) |
| Rpim | 0.04311 (0.6775) | 0.05694 (0.6918) | 0.0604 (0.5272) |
| CC1/2 | 0.996 (0.54) | 0.998 (0.576) | 0.996 (0.608) |
| **Refinement statistics** | | | |
| Reflections used in refinement | 29353 (2942) | 14281 (2752) | 25257 (2496) |
| Reflections used for Rfree | 1454 (137) | 705 (114) | 1169 (103) |
| Rwork | 0.2339 (0.3842) | 0.2267 (0.3809) | 0.2206 (0.3292) |
| Rfree | 0.2745 (0.3950) | 0.2527 (0.4263) | 0.2525 (0.3720) |
| Number of non-hydrogen atoms | 4674 | 3280 | 5014 |
| Macromolecules | 4632 | 3247 | 4764 |
| Ligands | 28 | 28 | 42 |
| Solvent | 14 | 5 | 208 |
| Protein residues | 623 | 438 | 630 |
| RMSD from ideal values | | | |
| Bond length (Å) | 0.002 | 0.003 | 0.003 |
| Bond angles (°) | 0.54 | 0.55 | 0.55 |
| Ramachandran favored (%) | 97.56 | 95.14 | 97.41 |
| Ramachandran allowed (%) | 2.11 | 3.7 | 2.43 |
| Ramachandran outliers (%) | 0.33 | 1.16 | 0.16 |
| Average B-factor | 83.63 | 152.76 | 57.81 |
| Macromolecules | 83.51 | 152.63 | 57.92 |
| Ligands | 105.92 | 170.52 | 72.03 |
| Solvent | 80.15 | 134.04 | 52.32 |

*Values in parentheses are for the highest-resolution shell.

Supplementary Table 12. Summary of PBMC characterization.

| Figure | Fig. 1C-D, Fig. 7D, Supplementary Fig. 16A | Fig. 1C-D | Fig. 3C-D, Fig. 5A-E, Fig. 5G-H, Fig. 7D-E, Supplementary Fig. 6A, Supplementary Fig. 9B-C, Supplementary Fig. 16A-B | Fig. 3C, Supplementary Fig. 6A | Fig. 5A-E, Fig. 5G-H, Supplementary Fig. 6A, Supplementary Fig. 9A-B | Supplementary Fig. 12A-B | Supplementary Fig. 12A-B | Fig. 7D, Supplementary Fig. 16A | Fig. 7D-E, Supplementary Fig. 16A | Fig. 7E, Supplementary Fig. 16B | Fig. 7E, Supplementary Fig. 16B | Fig. 7E, Supplementary Fig. 16B | Fig. 8A-B, Supplementary Fig. 18 | Fig. 8A-B, Supplementary Fig. 18 | Fig. 8A-B, Supplementary Fig. 18 |
| --- | --- | --- | --- | --- | --- | --- | --- | --- | --- | --- | --- | --- | --- | --- | --- |
| NKG2C | - |  | **+** |  |  |  |  | - | **+** | - | - | - |  |  |  |
| NKG2A | **+** | - | - |  |  |  |  | **+** | - | **+** | **+** | **+** |  |  |  |
| KIR3DS1 |  |  |  |  |  | **+** | **+** |  |  |  |  |  |  |  |  |
| KIR2DS4 |  |  | - | - |  |  |  |  |  |  |  |  | **+** | **+** | **+** |
| KIR2DS1 |  |  | **+** | - |  |  |  |  |  |  |  |  | **+** | **+** | **+** |
| KIR3DL1 |  |  |  |  |  | - | - |  |  |  |  |  | **+** | **+** | **+** |
| KIR2DL2/3/S2 | **+** |  | **+** | **+** |  |  |  | **+** | **+** | **+** | **+** | **+** | **+** | **+** | **+** |
| KIR2DL1 |  |  |  |  |  |  |  |  |  |  |  |  | **+** | **+** | **+** |
| HLA genotype | C2/C2/BW4+ | C2/C2//BW4+ | C1/C1/BW4- | C1/C1/BW4- | C2/C2//BW4- | C2/C2//BW4+ | C1/C1/BW4+ | C2/C2//BW4+ | C2/C2//BW4+ | C1/C1/BW4- | C1/C1/BW4- | C1/C1/BW4- | C1/C2/BW4+ | C1/C2/BW4+ | C1/C2/BW4+ |
| PBMC name | PBMC-A | PBMC-B | PBMC-C | PBMC-D | PBMC-E | PBMC-F | PBMC-G | PBMC-H | PBMC-I | PBMC-J | PBMC-K | PBMC-L | PBMC-M | PBMC-N | PBMC-O |
