# Supplementary material for: Selective agonists of KIR and NKG2A to evade missing self response of natural killer cells
Source: Sci Rep. 2025 Sep 29;15:33550. doi: 10.1038/s41598-025-18394-z (PMC12480839; doi:10.1038/s41598-025-18394-z)
Supplement: Supplementary file 2 — Supplementary Material 2 [file 41598_2025_18394_MOESM2_ESM.docx]

**Supplemental Method**

**Hydrogen-deuterium exchange mass spectrometry**

HDX-MS experiments were performed using a Synapt G1 HDMS coupled to a nanoACQUITY UPLC HDX System with Leap Technology (Waters Corporation). Briefly, hKIR2DL2(22–225)-His and KIR2DL2/3 agonist 61 scFv in D-PBS(-) were incubated for 30 s, 1 min, 10 min, 60 min, and 240 min in deuterium-labeled buffer L [D-PBS(-) in D_2_O pD 7.4] before quenching. Then, the protein was digested by pepsin and peptides were trapped in a UPLC column. Then, the peptides underwent electrospray ionization in positive ion mode using a Synapt G1 mass spectrometer (Waters Corporation). Fibrinopeptide was applied for lock mass correction and MS^E^ data were collected from 100 to 2000 m/z. Acquired reference MS^E^ data were analyzed by ProteinLynx Global Server (PLGS, ver3.0.3) (Waters Corporation) to identify the peptic peptides, and then all of the MS^E^ data were processed by DynamX ver.3.0 (Waters Corporation) to determine deuterium uptake. The mass difference of each peptide (D𝐻𝑋) at each deuterium exchange time was calculated as follows.

D𝐻𝑋 = m_hKIR2DL2_ − m_hKIR2DL2 with KIR2DL2/3 agonist 61 scFv_

Statistical analysis was performed as reported previously^supplemental1^.

**NK cell survival assay**

The viability of NK92 cells in the NK cell cytotoxicity assay was analyzed by time-resolved fluorescence using N-SPC Non-Radioactive Cellular Cytotoxicity Assay Kit (Techno Suzuta, NSPC-01). Briefly, NK92 cells were prepared for the cytotoxicity assay and resuspended in medium containing 2.5 μl/ml BM HT Reagent for 15 min. Then, the NK92 cells were washed twice with medium. The NK92 cells and target K562 cells were harvested and resuspended in fresh culture medium. A total of 5 × 10^4^ NK92 cells and K562 cells in 100 μl of culture medium were applied to a round-bottomed 96-well plate and co-cultured for 3 h at 37°C under 5% CO_2_. Twenty minutes before detection of time-resolved fluorescence and luminescence, detergent reagent was applied to control max. emission wells following the kit’s protocol. Three hours later, an appropriate volume of supernatant was collected and mixed with Eu Solution. Then, time-resolved fluorescence was detected using a FlexStation 3 multi-mode microplate reader (Molecular Devices), in accordance with the manufacturer’s instructions. The percentage of specific NK92 cell lysis was calculated as follows.

Specific lysis (%) = 100 × [(Time-resolved fluorescence of NK92 cells plus K562 cells) − (Basal time-resolved fluorescence of NK92 cells only)] / [(Max. emission time-resolved fluorescence of NK92 cells only) − (Basal time-resolved fluorescence of NK92 cells only)].

**Supplemental reference**

1. Hageman, T. S. & Weis, D. D. Reliable identification of significant differences in differential hydrogen exchange-mass spectrometry measurements using a hybrid significance testing approach. *Anal. Chem.* **91**, 8008–8016 (2019).
